# Evaluation of antibacterial, cytotoxicity, and apoptosis activity of novel chromene-sulfonamide hybrids synthesized under solvent-free conditions and 3D-QSAR modeling studies

**DOI:** 10.1038/s41598-024-63535-5

**Published:** 2024-06-05

**Authors:** Shakila Ghomashi, Reihane Ghomashi, Mohammad Sadegh Damavandi, Zeynab Fakhar, Seyedeh Yasaman Mousavi, Azhar Salari-Jazi, Sajjad Gharaghani, Ahmad Reza Massah

**Affiliations:** 1 Department of Medicinal Chemistry, Shahreza Branch, Islamic Azad University, P.O. Box 311-86145, Shahreza, Isfahan Iran; 2 Department of Medicinal Chemistry, School of Pharmacy and Pharmaceutical Sciences, Isfahan University of Medical Sciences, Isfahan, Iran; 3 Department of Microbiology and Virology, Faculty of Medicine, Mashhad University of Medical Sciences, Mashhad, Iran; 4 Antimicrobial Resistance Research Center, Mashhad University of Medical Sciences, Mashhad, Iran; 5 Laboratory of Bioinformatics and Drug Design (LBD), Institute of Biochemistry and Biophysics, University of Tehran, Tehran, Iran; 6 Department of Animal Biology, Faculty of Natural Sciences, University of Tabriz, Tabriz, Iran; 7 Faculty of Medicine and Health Technology, Tampere University, Tampere, Finland; 8 Department of Drug Development and Innovation, Behban Pharmed Lotus, Tehran, Iran; 9 Department of Chemistry, Shahreza Branch, Islamic Azad University, P.O. Box 311-86145, Shahreza, Isfahan Iran; 10 Department of Chemistry, Brock University, St. Catharines, ON Canada

**Keywords:** Chromenes, Sulfonamides, Solvent-free conditions, Synthesis, Antibacterial, Cytotoxicity activity, Apoptosis activity, Docking, ADMET, Pharmacokinetics, 3D-QSAR modeling studies, Chemical biology, Computational biology and bioinformatics, Drug discovery, Molecular medicine, Chemistry

## Abstract

In this study, eleven novel chromene sulfonamide hybrids were synthesized by a convenient method in accordance with green chemistry. At first, chromene derivatives (**1**–**9a**) were prepared through the multi-component reaction between aryl aldehydes, malononitrile, and 3-aminophenol. Then, synthesized chromenes were reacted with appropriate sulfonyl chlorides by grinding method to give the corresponding chromene sulfonamide hybrids (**1**–**11b**). Synthesized hybrids were obtained in good to high yield and characterized by IR, ^1^HNMR, ^13^CNMR, CHN and melting point techniques. In addition, the broth microdilution assay was used to determine the minimal inhibitory concentration of newly synthesized chromene-sulfonamide hybrids. The MTT test was used to determine the cytotoxicity and apoptotic activity of the newly synthesized compounds against fibroblast L929 cells. The 3D‑QSAR analysis confirmed the experimental assays, demonstrating that our predictive model is useful for developing new antibacterial inhibitors. Consequently, molecular docking studies were performed to validate the findings of the 3D-QSAR analysis, confirming the potential binding interactions of the synthesized chromene-sulfonamide hybrids with the target enzymes. Molecular docking studies were employed to support the 3D-QSAR predictions, providing insights into the binding interactions between the newly synthesized chromene-sulfonamide hybrids and their target bacterial enzymes, thereby reinforcing the potential efficacy of these compounds as antibacterial agents. Also, some of the experimental outcomes supported or conflicted with the pharmacokinetic prediction (especially about compound carcinogenicity). The performance of ADMET predictor results was assessed. The work presented here proposes a computationally driven strategy for designing and discovering a new sulfonamide scaffold for bacterial inhibition.

## Introduction

Antibiotic resistance, which was made worse by overuse and misuse of the drugs, is now a major public concern^1^. Antibacterial medications have been losing their efficiency against diseases since new reports of bacteria that are resistant to antimicrobial treatments appear every day. Frequently, these bacteria exhibit multi-drug resistance (MDR), extensive drug resistance (XDR), or pan-drug resistance (PDR), resulting in a progressively narrowed effective spectrum of common antibiotics^1–3^. Heterocycle compounds are one of the most important classes of organic compounds because of their vital role in life and biochemical processes^4^. Among the different heterocycle compounds in medicinal chemistry, sulfonamides have been important motifs since the initial discovery of sulfonamide-containing antibacterial drugs^5^. Many sulfonamide hybrids as antibacterial drugs have been synthesized by using coupling between heterocycle primary amine and aromatic sulfonyl chloride^6–10^. In addition to antibacterial activity, sulfonamides can be used as antifungal^11^, antimalarial^12^, antioxidant^13^, antiviral^14^, anti-inflammatory^15^, antitumor^16^, and many other pharmaceutically active agents.

**Scheme 1:**
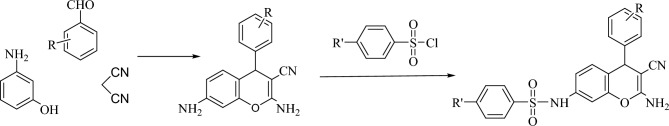
Synthesis of novel chromene-sulfonamide hybrids.

Moreover, Chromene derivatives are an essential class of oxygen-containing heterocyclic compounds that have attracted researcher's attention because of their applications in pigments, cosmetics, and agrochemicals, and their wide range of biological activities^17,18^ like anti-diabetic^19^, antibacterial^20^, antioxidant^21^, anti-cancer^22^ and antiviral activities^23^. Given that chromene sulfonamide hybrids have been shown variable biological activities^24–26^, and based on our previous works on the synthesis of sulfonamide hybrids^27–31^, accordingly, we were motivated to design and synthesize some novel derivatives of these hybrids (Scheme 1).

The Computer-aided drug design (CADD) can not only preliminarily predict the activity of inhibitors, but also save experimental costs and provide guidance for designing more effective inhibitors by exploring the structure–activity relationship of inhibitors at the molecular level^32^.

In this regard, in the first step of the present study, the 3D-QSAR model, and ADMET (absorption, distribution, metabolism, and excretion) were produced to predict the antibacterial activity impact of the considered compounds. In addition, the antibacterial of the synthesized compounds was screened against *Staphylococcus aureus* as Gram-positive and *Escherichia coli* as Gram-negative bacteria. Continuing our previously published study^2^ on the evaluation of the bioactivity impact of some novel coumarin isoxazole sulfonamide hybrid compounds, 3D-QSAR analysis was performed on the novel chromene-sulfonamide hybrid compounds. The accuracy of the 3D-QSAR model was high, as evidenced by the strong correlation between predicted and experimental activities, with high R^2^ and Q^2^ values indicating reliable predictive power. The newly synthesized compounds were also tested for cytotoxicity against fibroblast L929 cells using the MTT method. Additionally, the Annexin V binding assay was performed for detecting apoptosis. Also, the accuracy of the predicted ADMET (using the ADMET predictor) was compared with the observed mood.

## Results and discussion

### Chemistry

**Scheme 2:**
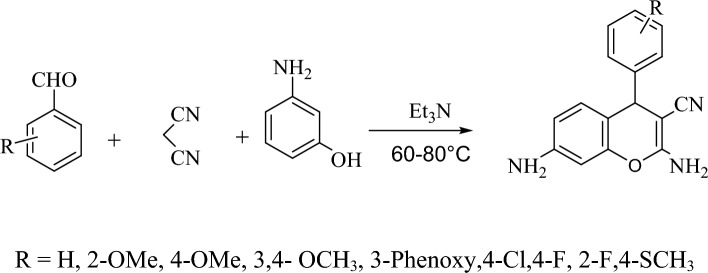
The synthetic route of the synthesized chromene derivatives.

The synthetic strategies adopted to obtain the target sulfonamide hybrids (**8a–l**) are depicted in Scheme 2. In the present study, green methods were applied to synthesize new chromene-sulfonamide hybrids. The synthesis of target sulfonamides was accomplished in two steps. The first one, chromene compounds were synthesized using a one-pot reaction between an aromatic aldehyde, malononitrile, and aminophenol^33,34^. In the presence of triethylamine as a base, the reaction was carried out in ethanol under reflux conditions (Scheme 2). Several aryl aldehydes containing electron-donating substituents such as methoxy and phenoxy group and also electron-withdrawing substituents such as halogens were used to obtain high yield and purity of the corresponding amino chromenes (Table 1).

**Table 1:** Yield/reaction time data for the preparation of chromene derivatives (**1–9a**).

Entry	Aldehyde	Product	Time (min)	Yield^a^ (%)	M.P. (°C) observed	M.P. (°C) reported [ref.]
1		**1a**	30	88	215–216	218–219^35^
2		**2a**	35	84	217–218	–
3		**3a**	60	86	194–196	–
4		**4a**	45	92	223–224	226–227^35^
5		**5a**	25	82	234–235	236–237^35^
6		**6a**	50	70	185–186	–
7		**7a**	30	88	196–197	196–197^36^
8		**8a**	35	89	247–248	–
9		**9a**	30	86	232–233	–

^a^Isolated yield.

**Scheme 3:**
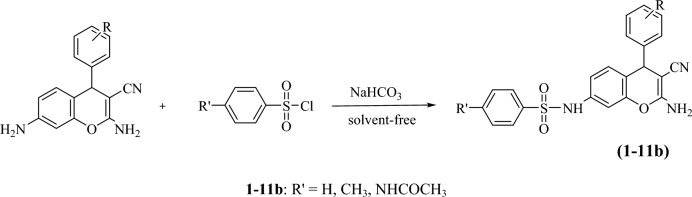
The synthetic route of the synthesized chromene-sulfonamide hybrids.

Then, to synthesize chromene sulfonamide hybrids, the synthesized amino chromenes were reacted with various aryl sulfonyl chlorides including benzenesulfonyl chloride, 4-toluenesulfonyl chloride, and 4-acetamidobenzenesulfonyl chloride under solvent-free conditions in the presence of sodium bicarbonate as a green base (Scheme 3). The chromene sulfonamide hybrids were obtained in 70–90% yield just by adding water and simple filtration in high purity (Table 2).

**Table 2:**
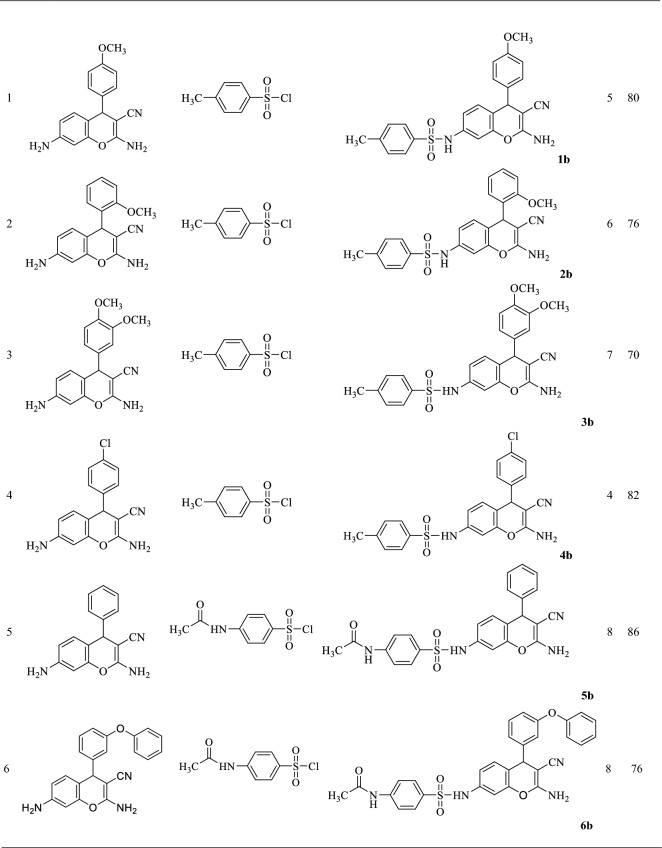

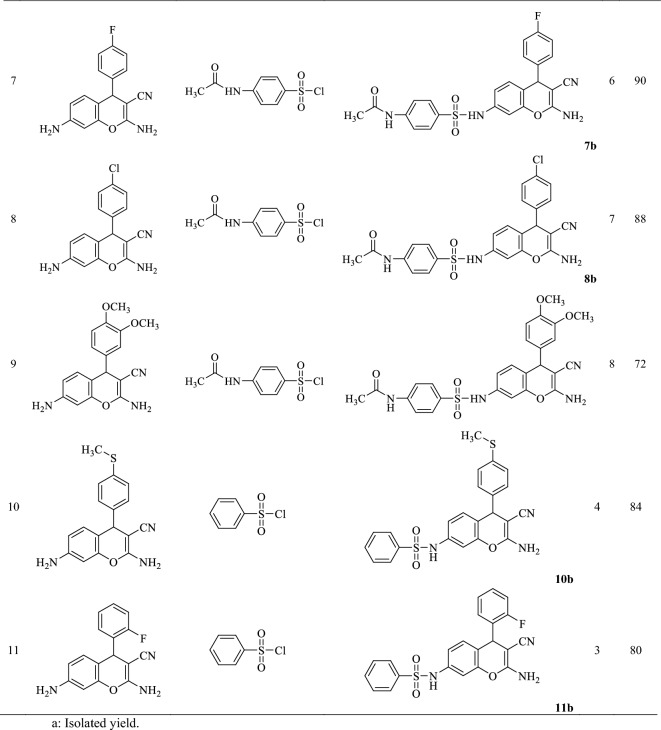
Yield/reaction time data for the preparation of chromene-sulfonamide hybrids (**1–11b**).

^a^Isolated yield.

### D-pharmacophore alignment of the compounds

The co-crystalized reference inhibitor was used for a 3D-pharmacophore analysis and seven features were produced based on the receptor-ligand pharmacophore generation protocol. The generated 3D-pharmacophore features include one H-bond acceptor, three H-bond donors, and three π–π stacking motifs (aromatic rings). The obtained 3D hypotheses were performed to align the compounds. The aligned compounds were applied for 3D-QSAR analysis.

### 3D-QSAR model development and statistical analysis

**Figure 1:**
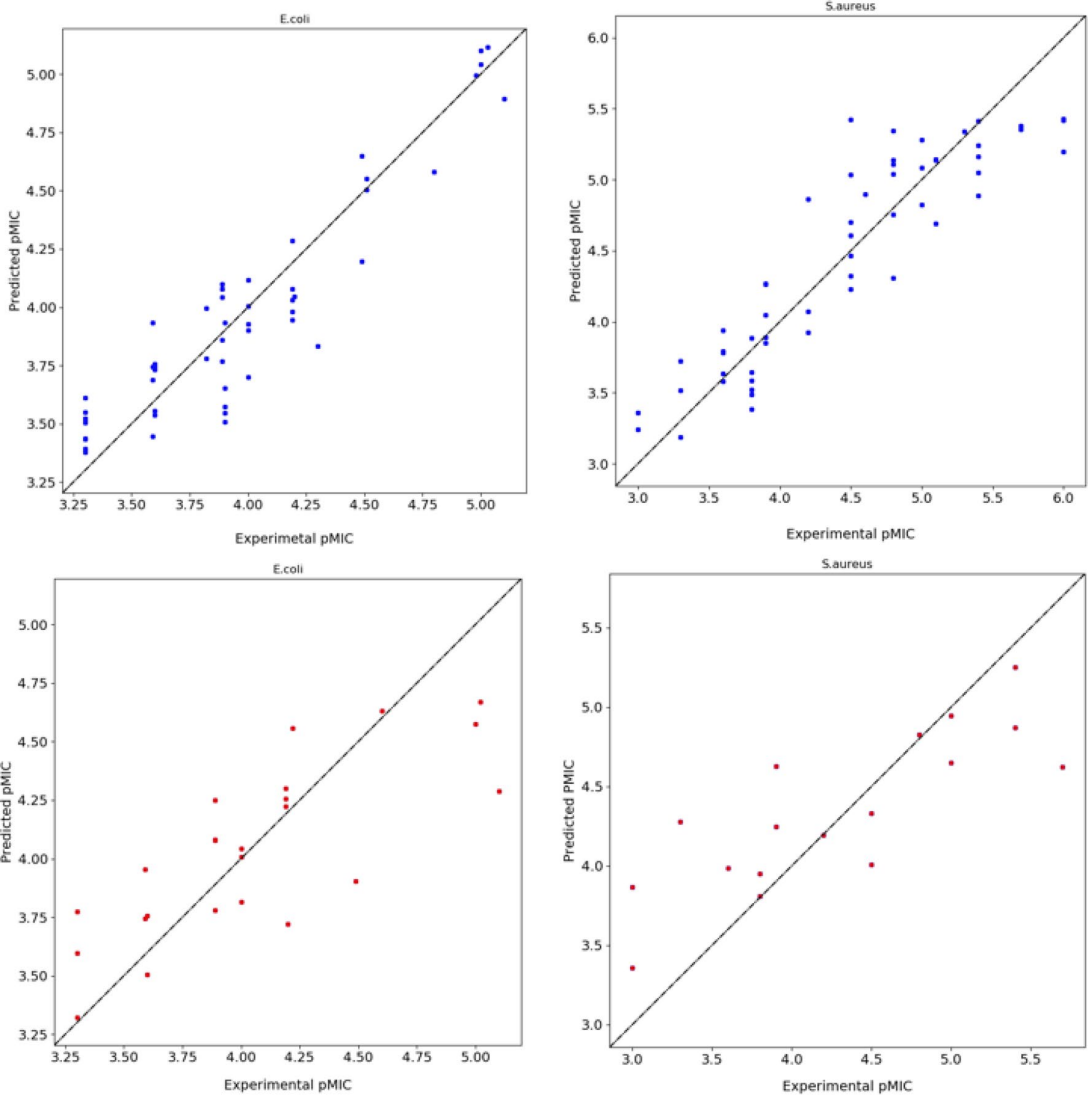
Plots of experimental activities against predicted activities using our COMSIA predictive model for both *E. coli* (left) and *S. aureus* (right). The train set is in blue and the test set is in red coded.

The Comparative Molecular Similarity Indices Analysis (CoMSIA) generated model had a strong predictive and informative ability, presenting good statistical analysis for the training set regression coefficient R^2^ (0.86, 0.82), test set regression coefficient Q^2^ (0.65, 0.67) and cross validation-based root mean square error (RMSE) values (0.32, 0.52) in both *E. coli* and *S*. aureus, respectively, Fig. 1.

The 3D-QSAR model outcomes thoroughly established its predictive capacity for that. The comparison of the predictive versus observed plot is illustrated in Fig. 1. The contributions of steric (38%, 39%), electrostatic (9%, 11%,), hydrophobic (22%, 19%), H-donor (18%, 13%), and H-acceptor (12%, 19%) fields of the compounds in both *E. coli* and *S*. aureus exhibited that the steric, H-bond donor/acceptor, and hydrophobic fields participated a crucial role in the model. Among these three fields, the steric field produced the most considerable interactions. The predicted pMIC values of the selected predictive model for both *E. coli* and *S.* aureus are placed in the Supplementary material.

### CoMSIA contour maps

**Figure 2:**
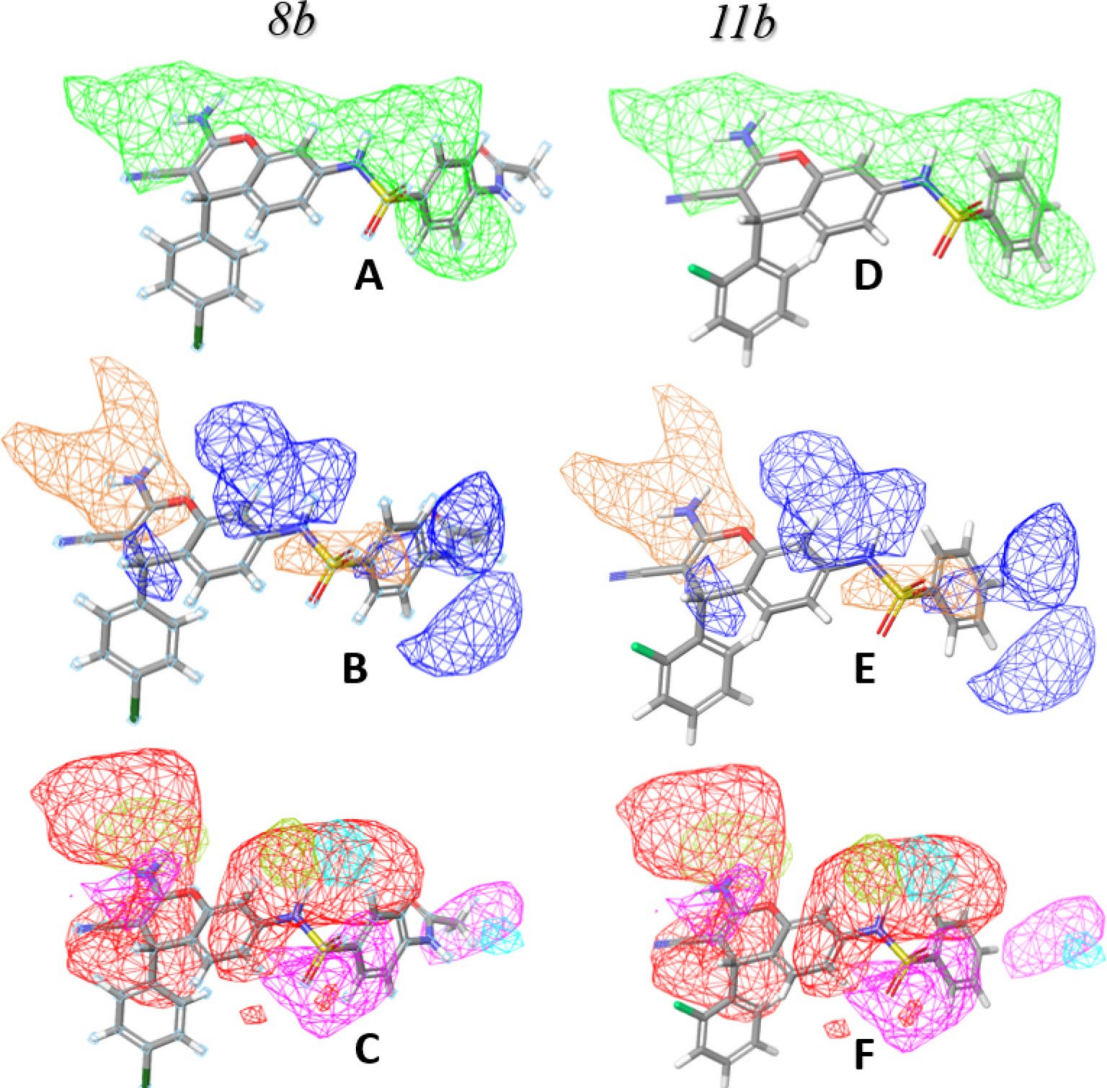
**8b** and **11b** Structures. CoMSIA contour maps. Steric contour maps: green and yellow played the favorable and unfavorable region; Hydrophobic contour maps: blue and orange indicated favorable and unfavorable region; Hydrogen bond acceptor contour maps: red and magenta indicated favorable and unfavorable region; Hydrogen bond donor contour maps: yellow-green and cyan indicated favorable and unfavorable region. (**A**, **B**, **C**) are contour maps for compound **8b** and (**D**, **E**, **F**) are represented contour maps for **11b**.

The activity contour map visualization method is a qualitative and beneficial procedure to summarize SAR data into a 3D map. In this analysis, compounds **8b** and **11b** were selected as the active compounds with the same biological activity values in both *E. coli* and *S. aureus*. Consequently, this method would probe the beneficial functional groups, impacting the potent activity. Figure 4 depicts the steric, hydrophobic, and hydrogen bond donor/acceptor fields contour maps for **8b** and **11b**. In the steric field, a green contour indicates the regions where bulky groups enhance biological activity (Fig. 2A,D). Figure 2B and E are the contour map of the hydrophobic field. The blue block means that the hydrophobic groups were beneficial for activity, while the orange block means the hydrophilic groups are not beneficial. As depicted, the 2-amino-3-cyano functional group of chromene moiety in both compounds had a negative impact on their biological activity, while, the phenyl acetoamide group in compound **8b** and benzene group in **11b** mediated positive impact.

Figure 2C and F are the contour map of the hydrogen bond donor field, indicating the favorable field with the yellow-green color and unfavorable region with cyan color whereas the hydrogen bond acceptor shows the beneficial and non-beneficial regions as red and magenta colors, respectively. As it is shown, the 2-amino-3-cyano functional group of chromene moiety in both compounds that are considered a favorable group in hydrogen bond acceptor fields; however, the methyl group linked to the carbonyl functional group of phenyl acetamide moiety acts as an unfavorable functional. In addition, the 2-amino functional group in chromene moiety is depicted as a beneficial group in both compounds.

### In silico pharmacokinetics compliance evaluation

The ADMET Predictor software assessed pharmacokinetic parameters in ADMET terms (absorption, distribution, metabolism, elimination, and toxicity) for all compounds are shown in supplementary information S1–S7. Physicochemical and biopharmaceutical properties were performed to determine the ADMET parameters for the studied compounds. The solubility properties which were identified and quantitatively reported (Table S1), including native water solubility (Table S1), fasting state intestinal fluid, fed state intestinal fluid, and fasted state stomach fluid. The values of the MLogP (Moriguchi model of octanol–water partition coefficient), octanol–water partition coefficient, octanol–water distribution coefficient, and molecular diffusion coefficient in the water logarithm of the air–water partition coefficient were established (Table S5). Table S3 contains the ionization level (pKa), which has a significant impact on solubility and permeability. A solubility and permeability optimal range was often observed in all substances. Except for **2b, 3b, 6b,** and **9b** other compounds commonly exhibited high BBB‌ penetration, accordingly **5b**, **1b**, **7b**, **8b**, **10b**, and **11b** were fit in brain infections, especially in meningitis cases. The logarithm of the brain/blood partition coefficient (LogBB) confirmed that these compounds could penetrate in BBB.

A compound's ability to dissolve in a lipophilic (nonaqueous) environment is referred to as lipophilicity. The coordination of multiple drug pharmacological properties, including absorption, metabolism, permeability, solubility, distribution, plasma protein binding, elimination, and toxicity affects ADMET—several characteristics influencing solubility and permeability presented in Tables S2 and S3, respectively.

The membrane borders that drug compounds encounter include gastrointestinal epithelial cells, hepatocyte membrane, blood capillary wall, glomerulus, restrictive organ barriers (e.g., blood–brain barrier), and other target cells. The computed permeability estimations provide support for the interpretation of cell-based bioassays and ADMET outcomes. Human skin permeability, the MDCK COS permeability, BBB permeability (logBB), jejunal effective permeability, and (Peff) rabbit corneal permeability are among the several forms of permeability that were anticipated (Table S2). The metabolism of drugs has a significant impact on both drug-drug interactions and drug bioavailability. Drug bioavailability and drug-drug interactions are significantly influenced by metabolism. Cytochrome P450 enzymes (CYPs) are the most certainly the largest noticeable of phase I metabolism enzymes reacting to the predominance of drug Phase I metabolic transformations (Table S4). All studied compounds are substrates for CYP1A2 and CYP2C8 enzymes, and also, inhibitors for CYP1A2, CYP2C9, and CYP3A4 enzymes, therefore this property must be noticed. Since some drugs metabolize in the body by CYPs, accordingly these compounds can be respected as a co-treatment to augment the mentioned drugs' half-life and blood concentration. Inhibition of the CYP enzymes (especially about CYP3A4 enzyme which is the most prominent role in the drug Phase I metabolic transformations) exhibited the potential high rate half-life of studied compounds. These properties can reduce the effective dose in the human body, and consequently, reduce the potential cost for compound usage.

Additionally, the activity of the uridine 5′-diphosphate-glucuronosyltransferase family (UGT) enzyme, which binds glucuronic acid to small molecules while modifying their structure into one that is water-soluble form, was further explored to evaluate phase II metabolism influence on the researched compounds, consequently, this phase has made it easier for drugs to be eliminated and extruded the outside of body cells.

Table S5 indicates that none of the investigated compounds performed as UGT enzyme substrates, with the exception of the UGT1A3 enzyme. This outcome confirmed that these compounds had long half-life.

Table S6 exhibited the Possibility of being a substrate and inhibitor of compounds for P-gp efflux. All compounds not only did not substrate for the P-gp efflux pump, but also, they were inhibitors for this pump.

These outcomes were corroborated with our previous data that displayed studied compounds had long half-life and also are good options for co-treatment with some other drugs that needed to obtain high concentration levels in the blood flow since our compounds can block possible mechanisms for clearance and elimination of other drug like neurodegenerative drugs (Parkinson and Alzheimer disease).

In Table S7, potential clearance methods were presented. Except for **5b**, **6b**, **7b**, **8b**, and **9b**, all of our compounds had no renal clearance, and none of them were not eliminated by hepatic metabolism.

The studied compounds were CYP enzyme inhibitors, which potentially elevate some drug plasma concentrations (drugs that CYPs were responsible for their metabolism). Accordingly, there could be a significant probability of drug-drug interaction (DDI) with simultaneous usage.

Based on the findings of the S + CL_Metab, CYP_RLM_Clint CYP_HLM_Clint, and S + CL_Mech analyses, which are included in Table S7, indicated substance clearance would most likely be concluded in the liver, however, **8b** and **9b** compounds had renal clearance possibility.

### Predicted toxicology of identified leads

Presented study compounds were subjected to estimate the mutagenicity, allergenicity, and toxicity in this stage.

All of the anticipated adverse effects can be found in Table 3. There is in-silico evidence of hERG (human ether-a-go-go-related gene) suppression only in compound **6b**, suggesting that there would not be a negative cardiac consequence, accordingly, this compound must be noted to screen subjected disease for cardiac health care. The rest of the compounds did not display cardiac adverse effects.

**Table 3:** The ADMET Predictor toxicity analyses of compounds.

Parameters	Compounds										
	**2b**	**3b**	**5b**	**1b**	**4b**	**6b**	**7b**	**8b**	**9b**	**10b**	**11b**
Cardiac											
hERG_Filter^1^	No (77%)	No (87%)	No (81%)	No (87%)	No (56%)	Yes	No	No (56%)	No	No (87%)	No (77%)
hERG pIC_50_^2^ (mol/L)	5.391	5.473	5.82	5.517	5.936	5.896	5.761	5.928	5.498	5.538	5.628
Endocrine											
ERs^3^	Nontoxic (77%)	Nontoxic (77%)	Nontoxic	Nontoxic (80%)	Nontoxic (77%)	Nontoxic	Nontoxic	Nontoxic	Nontoxic	Nontoxic (71%)	Nontoxic (73%)
Estro_RBA^4^	Nontoxic	Nontoxic	Nontoxic	Nontoxic	Nontoxic	Nontoxic	Nontoxic	Nontoxic	Nontoxic	Nontoxic	Nontoxic
ARS^5^	Nontoxic (75%)	Nontoxic (75%)	Nontoxic (61%)	Nontoxic (72%)	Nontoxic (65%)	Nontoxic (49%)	Nontoxic (69%)	Nontoxic (59%)	Nontoxic (67%)	Nontoxic (69%)	Nontoxic (79%)
Andro_RBA^6^	Nontoxic	Nontoxic	Nontoxic	Nontoxic	Nontoxic	Nontoxic	Nontoxic	Nontoxic	Nontoxic	Nontoxic	Nontoxic
Hepatotoxicity											
AlkPhos^7^	Normal (92%)	Normal (92%)	Normal (92%)	Normal (92%)	Normal (83%)	Normal (92%)	Normal (92%)	Normal (83%)	Normal (92%)	Normal (83%)	Normal (92%)
ALT^8^	Normal (94%)	Normal (94%)	Normal (94%)	Normal (94%)	Normal (94%)	Normal (86%)	Normal (94%)	Normal (94%)	Normal (86%)	Normal (81%)	Normal (94%)
AST^9^	Elevated (44%)	Normal	Normal	Normal	Normal (61%)	Normal	Normal	Normal (72%)	Normal	Normal	Elevated (53%)
LDH^10^	Normal (83%)	Normal (79%)	Normal (74%)	Normal (83%)	Elevated (46%)	Normal (68%)	Normal (79%)	Normal (56%)	Normal (70%)	Normal (83%)	Normal (94%)
GGT^11^	Normal (97%)	Normal (90%)	Normal (82%)	Normal (97%)	Normal (82%)	Normal	Normal (86%)	Normal (82%)	Normal (86%)	Normal (80%)	Normal (82%)
Reproductive											
Repro_Tox^12^	Toxic (91%)	Toxic (91%)	Toxic (81%)	Toxic (91%)	Toxic (91%)	Toxic	Toxic	Toxic (91%)	Toxic	Toxic (91%)	Toxic (91%)
Carcinogenicity											
Rat-TD50 (mg/kg/day)	11.566	10.563	10.196	11.788	14.627	8.154	12.785	11.117	8.245	9.658	23.966
Mouse-TD50 (mg/kg/day)	688.149	406.853	321.097	347.869	535.804	339.318	360.511	479.805	355.905	89.988	688.574
Chrom_Aberr^13^	Toxic (60%)	Toxic (51%)	Toxic (65%)	Toxic (56%)	Toxic (46%)	Toxic (54%)	Toxic (54%)	Toxic (58%)	Toxic (58%)	Nontoxic (48%)	Toxic (54%)
MUT	Negative	Negative	Negative	Negative	Negative	Negative	Negative	Negative	Negative	Negative	Negative
Sensitization											
Sens_Skin^14^	Nonsensit. (58%)	Nonsensi.(58%)	Sensitizer (56%)	Nonsensit. (61%)	Nonsensit. (61%)	Nonsensit. (61%)	Sensitizer (56%)	Sensitizer (56%)	Nonsensit. (58%)	Nonsensit. (58%)	Nonsensit. (58%)
Environmental											
Biodegradn^15^	No (95%)	No (95%)	No (95%)	No (95%)	No (95%)	No (95%)	No (95%)	No (95%)	No (95%)	No (95%)	No (95%)
Acute rat toxicity											
Rat_Acute^16^ (mg/kg body weight)	332.833	359.749	216.625	399.456	223.692	113.236	167.646	152.981	221.347	520.478	235.555

^1^hERG_Filter: the hERG affinity model

^2^hERG pIC_50_: Values for inhibition of hERG K^+^ channels

^3^ERs: Affinity to estrogen receptors

^4^Estro_RBA: Relative binding affinity-serum of estrogen receptors

^5^ARs: Affinity to androgen receptors

^6^Andro_RBA: Relative binding affinity-serum of androgen receptors

^7^AlkPhos: level of alkaline phosphatase enzyme

^8^ALT: level of alanine transaminase enzyme

^9^AST: level of aspartate transaminase enzyme

^10^LDH: level of LDH

^11^GGT: level of gamma-glutamyltransferase

^12^Repro_Tox: Reproductive toxicity

^13^Chrom_Aberr: chromosomal aberrations

^14^Sens_Skin: skin sensitization

^15^Biodegradn: biodegradation

^16^Rat_Acute: Acute Rat Toxicity.

The adverse clinical implications, including QT prolongation, myopathy, hepatotoxicity, nephrotoxicity, and pulmonary disorder, attributed to drug-induced phospholipidosis were not shown. ADMET predictor assessment for reproductive toxicity exhibited that all compounds were high-risk toxic, while; this outcome is incompatible with MTT and flow cytometry outcomes about **1b**, **2b**, **3b**, **5b**, **7b**, **9b**, **10b**, and **11b** that demonstrated the compounds were not toxic. Drug-induced hepatotoxicity is created through AST, ALT, ALP, and LDH, Ser_AlkPhos, or Ser_GGT enzymes augmented levels, causing acute and chronic liver disease. All compounds demonstrated liver enzymes’ standard value, accordingly, there is no anti-hepatotoxic possibility.

Androgen and estrogen receptor toxicity was not observed; therefore, compounds do not impact sperm concentration. Except for compounds **5b**, **8b,** and **9b** which exhibited skin sensitization, the rest of the compounds were safe in this case. The findings of the Ames test for mutagenicity on several strains of Salmonella typhimurium revealed that all studied compounds were safe. This finding is corroborated with the MTT and flow cytometry outcomes (special about about **1b**, **2b**, **3b**, **5b**, **7b**, **9b**, **10b** and **11b**) and also conflicted (about **1b**, **4b**, **6b**, and **8b**) with the ADMET predictor reproductive toxicity results.

### Molecular docking

The subjected compounds were docked in the DHPS enzymes of *S. aureus* and *E. coli* using Autodock Vina. All compounds exhibited favorable and negative binding energy (Table S9). The binding energies of these eleven compounds (designated as **1b**, **2b, 3b**, **4b**, **5b**, **6b**, **7b**, **8b**, **9b**, **10b**, and **11b**) with DHPS of *S. aureus* and *E. coli* DHPS were placed in the Table S9. The produced data through Ligplot illustrated the binding configuration of the compound, the hydrogen bonds and hydrophobic interactions between the ligands and the DHPS active site residues (Fig. S1, and S2 and Table S9).

Molecular docking was employed to analyze the binding affinity of these 11 compounds (**1b**, **2b**, **3b**, **4b**, **5b**, **6b**, **7b**, **8b**, **9b**, **10b**, and **11b**) with favorable docking scores. The docking results indicate that all eleven compounds exhibited favorable binding energies when docked with the DHPS enzymes of both *S. aureus* and *E. coli* using Autodock Vina (Table S1, and S2). The binding energies for these compounds ranged from − 13.4 to − 19.2 kcal/mol for *S. aureus* DHPS and from − 15 to − 18.6 kcal/mol for *E. coli* DHPS. The observed binding energies suggested that strong interactions between the compounds and the DHPS enzymes. Specifically, compounds **4b** and **5b** showed the highest binding energies for *S. aureus* DHPS, indicating potentially strong binding affinity. Similarly, compounds **4b** and **6b** exhibited the highest binding energies for *E. coli* DHPS.

These findings are significant as DHPS is a key enzyme involved in the biosynthesis of folate, which is essential for the growth and survival of bacteria. Inhibition of DHPS can therefore be a promising strategy for developing antibacterial agents. The favorable binding energies observed in this study implied that the eleven compounds identified could be potential candidates for further development as antibacterial agents targeting DHPS.

### Antibacterial activity evaluation

The broth microdilution method assessed the minimum inhibitory concentrations (MIC) of all the newly synthesized compounds. MIC results suggested that **8b** and **6b** have a robust antibacterial effect on *E. coli* and *S. aureus* and the rest of the compounds were moderately active against these bacteria**.** The compound **8b** exhibited a significant effect on the *E. coli* and *S. aureus* (Table 4). Sulfisoxazole, functioning as a sulfonamide antibiotic, and Gentamicin were employed in a comparative analysis of antibacterial activity, cytotoxicity, and apoptosis assessment.

**Table 4:** Minimum inhibitory concentrations (MIC) in µg/mL of compounds tested in microdilution method.

Compound no.	*E. coli* ATCC 25922	*S. aureus* ATCC 25923
**1b**	256	> 512
**2b**	128	> 512
**3b**	> 512	> 512
**4b**	128	256
**5b**	128	128
**6b**	64	256
**7b**	128	128
**8b**	**64**	**32**
**9b**	256	> 512
**10b**	256	> 512
**11b**	256	256
Sulfisoxazole	256	256
Gentamicin	4	4

Significant values are in bold.

Table 4 displays the IC_50_ of synthesized compounds. Also, a comparison of the mean IC_50_ values for newly synthesized compounds is placed in Fig. 1. The average cytotoxicity of the newly synthesized compounds was significant. MTT test results revealed that compounds **1b**, **4b**, **6b**, and **8b** have a higher cytotoxicity effect on the fibroblast L929 cell line compared to the rest compounds. This finding implied several points to attention. First, it is important to acknowledge that the toxicity of a compound depends on various factors, including its chemical structure, concentration, and the specific biological system it is being tested on. It would be valuable to know the specific structure and nature of the synthesized compounds and how they differ from other compounds that can produce augmented cytotoxicity levels.

### Cytotoxicity evaluation

**Figure 3:**
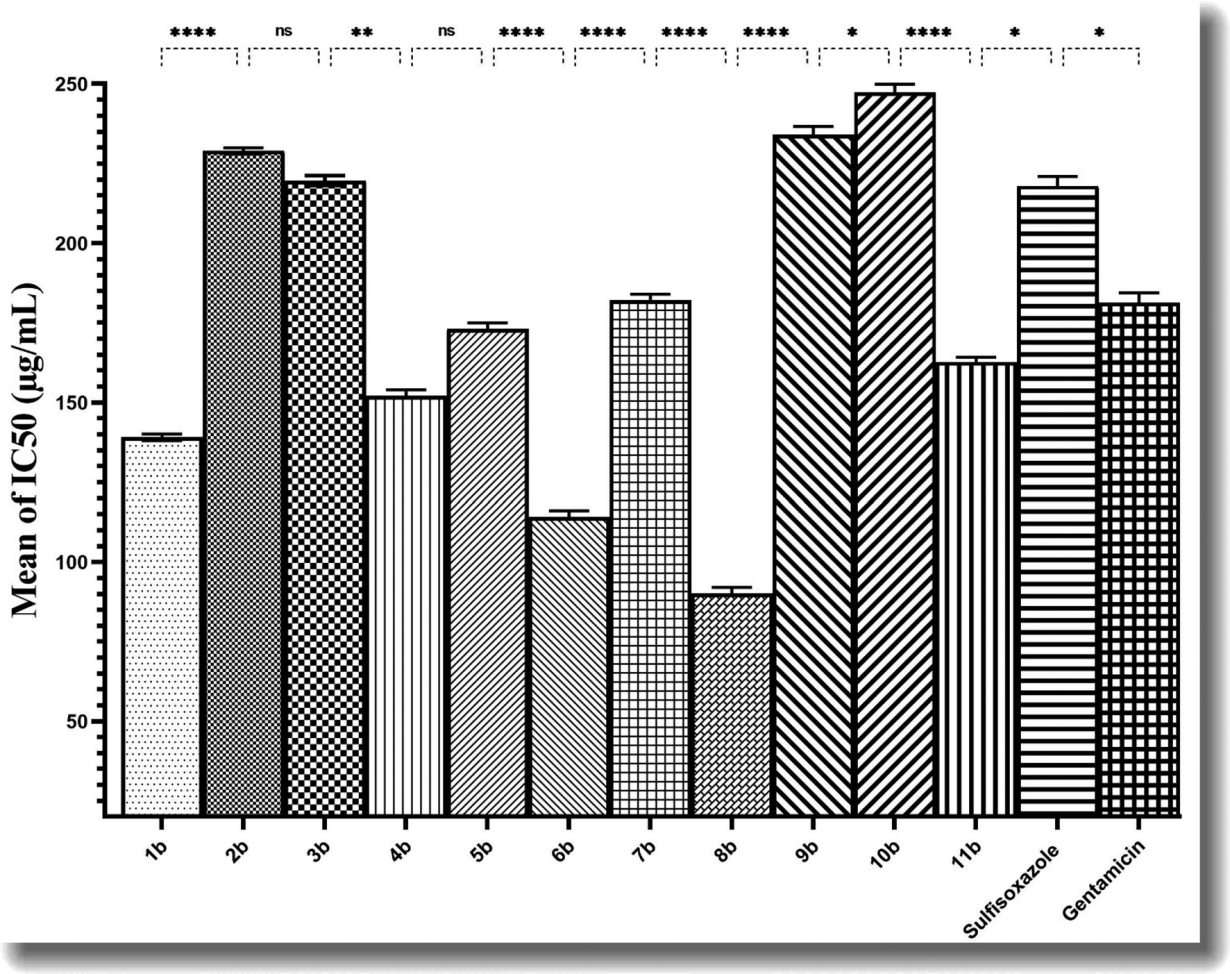
Comparison of mean IC_50_ of newly synthesized compounds. If the *p*-value is 0.05 or lower, the result is statistically significant. *ns* not significant. The results were subjected to analysis of one-way analysis of variance (ANOVA), followed by Tukey's test. The significance levels were indicated as follows: *P ≤ 0.05, **P ≤ 0.01, ***P ≤ 0.001, and ****P ≤ 0.0001. Sulfisoxazole, and Gentamicin were employed in a comparative analysis of cytotoxicity assessment.

Table 5 presents the IC_50_ values of the synthesized compounds, while Fig. 3 compares the mean IC_50_ values for these newly synthesized compounds. Notably, the average cytotoxicity of the newly synthesized compounds was found to be significant. The descriptive data and IC_50_ values for the synthesized compounds against the fibroblast L929 cell line. Compounds **1b**, **4b**, **6b**, and **8b** showed significant cytotoxicity with IC_50_ values of 139 µg/mL, 152 µg/mL, 114 µg/mL, and 90 µg/mL, respectively. These findings imply that structural elements within these compounds contribute to their enhanced cytotoxicity. This observation prompts several considerations. Firstly, it is crucial to recognize that the toxicity of a compound is influenced by various factors, including its chemical structure, concentration, and the specific biological system under investigation. Thus, understanding the specific structure and characteristics of the synthesized compounds, along with how they differ from other compounds with heightened cytotoxicity, is important. While increased cytotoxicity may raise safety concerns, there are contexts where it may be desirable, such as in the development of anticancer drugs or antimicrobial agents. Understanding the SAR and conducting in-depth analyses will aid in optimizing these compounds for desired biological activities while minimizing potential adverse effects. Evaluating the effects of these compounds on other cell lines and conducting animal studies to evaluate systemic toxicity would offer a comprehensive understanding of their safety profile.

**Table 5:** Descriptive data and IC_50_ of newly synthesized compounds against fibroblast L929 cell line.

Compounds name	Mean IC_50_ (µg/ml)	SD^a^	SD error of mean	Lower 95% CI^b^	Upper 95% CI	*p*-value
1b	139	1	0.5774	136.5	141.5	< 0.0001
2b	229	1	0.5774	226.5	231.5	
3b	219	1.528	0.8819	215.9	223.5	< 0.0057
4b	152	2	1.155	147.0	157.0	
5b	173	2	1.155	168.0	178.0	< 0.0001
6b	114	2	1.155	109.0	119.0	
7b	182	2	1.155	177.0	187.0	< 0.0001
8b	90	2	1.155	85.03	94.97	
9b	234	2.646	1.528	227.4	240.6	< 0.0464
10b	247	2.517	1.453	241.1	253.6	
11b	162	1.528	0.8819	158.9	166.5	< 0.0135
Sulfisoxazole	218	3	1.732	210.5	225.5	
Gentamicin	182	3.055	1.764	173.7	188.7	< 0.0347

^a^*SD* Standard Deviation, ^b^*CI* Confidence Interval.

### Apoptosis Annexin V binding evaluation:

**Figure 4:**
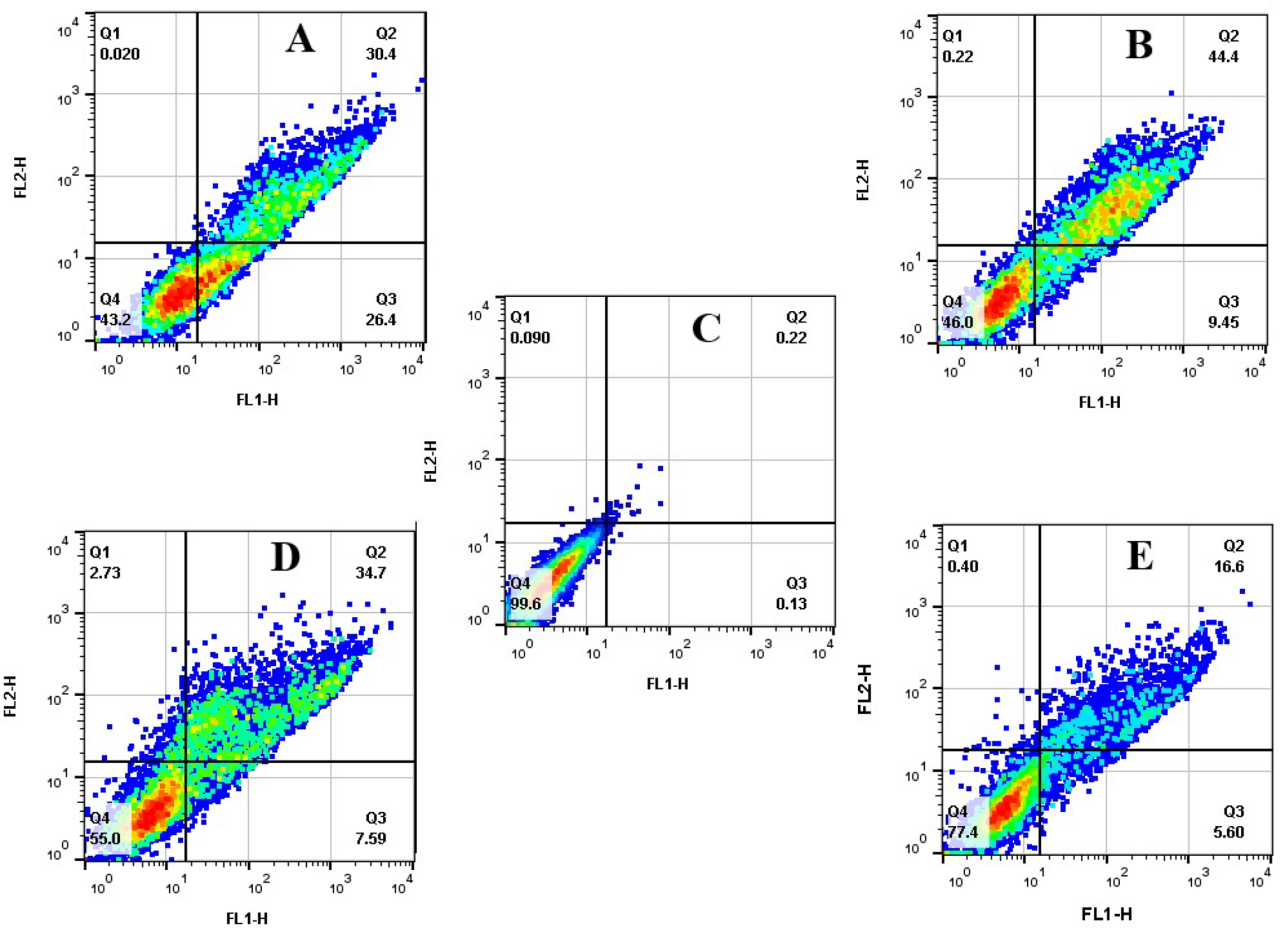
Two-dimensional plot of Annexin V-FITC against PI from flow cytometric experiments, with concentration values for the IC_50_ concentration of each compound. The FSC/SSC plot was used to determine the analysis border in relation to the untreated cells (control). By placing the most dots in the Q4 region of the control, the borders of the four quadrants (Q1–Q4) were determined. Based on cell stages, the cells were divided into four quadrants: viable (Q4), early apoptosis (Q3), late apoptosis (Q2), and necrosis (Q1). Data are presented as percentages. (**A**) IC_50_ concentration of **8b**, (**B**); IC_50_ concentration of **6b**, (**C**); Control, (**D**); IC_50_ concentration of **1b**, (**E**); IC_50_ concentration of **4b**.

The flow cytometry method was used to check apoptosis at the IC_50_ concentration of each compound. Based on this assay, four newly synthesized compounds (**1b**, **4b**, **6b**, and **8b**) were more cytotoxic for the fibroblast L929 cell line. The cells were labeled using Annexin V FITC and/or propidium iodide (PI) dyes. Figure 4 represents the occurred apoptosis event in treated and untreated fibroblast L929 cells after 24 h with each compound IC_50_ concentration. The result revealed that synthesized compounds **1b**, **4b**, **6b**, and **8b** induced cell death in with 42.29%, 22.2%, 53.85%, and 56.8%, respectively. At the IC_50_ concentration, neither Sulfisoxazole nor Gentamicin demonstrated apoptotic effects.

### SAR investigation on chromene sulfonamide derivatives

Compounds **2b**, **4b**, **5b**, **6b**, **7b**, and **8b** exhibit positive MIC effects on *E. coli*, indicating enhanced antibacterial activity. Compounds **3b**, **9b**, **10b**, and **11b** showed neutral or negative MIC effects, indicating reduced or no antibacterial activity. Steric and hydrophobic effects are generally positive for compounds with lower MIC values (e.g., **6b**, **8b**), suggesting these properties enhance antibacterial activity. Compounds with positive H-bond donor effects (e.g., **7b**, **8b**) also showed positive MIC effects, highlighting the importance of hydrogen bond interactions in antibacterial activity (Table 6).

**Table 6:** SAR effect diagram on the antibacterial activity of Chromene sulfonamides against *E. coli.*

Compound number	R	R'	MIC for *S. aureus* (µg/ml)	MIC effect on *E. coli*	MIC effect on *S. aureus*	Steric effect	Hydrophobic effect	H-bond donor effect	H-bond acceptor effect
1b	4-OCH_3_	CH_3_	256	Neutral	Negative	Neutral	Neutral	Negative	Positive
2b	2-OCH_3_	CH_3_	128	Positive	Negative	Positive	Positive	Negative	Positive
3b	3,4-OCH_3_	CH_3_	> 512	Negative	Negative	Negative	Negative	Negative	Negative
4b	4-Cl	CH_3_	128	Positive	Neutral	Positive	Positive	Positive	Neutral
5b	H	NHCOCH_3_	128	Positive	Positive	Positive	Positive	Positive	Neutral
6b	3-Phenoxy	NHCOCH_3_	64	Positive	Neutral	Positive	Positive	Positive	Neutral
7b	4-F	NHCOCH_3_	128	Positive	Positive	Positive	Positive	Positive	Positive
8b	4-Cl	NHCOCH_3_	64	Positive	Positive	Positive	Positive	Positive	Positive
9b	3,4-OCH_3_	NHCOCH_3_	256	Neutral	Negative	Neutral	Neutral	Negative	Negative
10b	4-SCH_3_	H	256	Neutral	Negative	Neutral	Neutral	Negative	Negative
11b	2-F	H	256	Neutral	Neutral	Neutral	Neutral	Neutral	Neutral

Table 7 presents the SAR effect diagram on the biological activity of compounds against *S. aureus.* Compounds **5b**, **7b**, and **8b** exhibit positive MIC effects on S. aureus, indicating enhanced antibacterial activity. Compounds **1b**, **2b**, **3b**, **9b**, and **10b** showed negative MIC effects, indicating reduced or no antibacterial activity. Similar to the *E. coli* results, steric and hydrophobic effects are generally positive for compounds with lower MIC values (e.g., **8b**), suggesting these properties enhance antibacterial activity. Compounds with positive H-bond donor effects (e.g., **7b**, **8b**) also showed positive MIC effects, highlighting the importance of hydrogen bond interactions in antibacterial activity.

**Table 7:** SAR effect diagram on the antibacterial activity of Chromene sulfonamides against S. aureus.

Compound number	R	R'	MIC for *S. aureus* (µg/ml)	MIC effect on *S. aureus*	Steric effect	Hydrophobic effect	H-bond donor effect	H-bond acceptor effect
1b	4-OCH_3_	CH_3_	> 512	Negative	Neutral	Neutral	Negative	Positive
2b	2-OCH_3_	CH_3_	> 512	Negative	Positive	Positive	Negative	Positive
3b	3,4-OCH_3_	CH_3_	> 512	Negative	Negative	Negative	Negative	Negative
4b	4-Cl	CH_3_	256	Neutral	Positive	Positive	Positive	Neutral
5b	H	NHCOCH_3_	128	Positive	Positive	Positive	Positive	Neutral
6b	3-Phenoxy	NHCOCH_3_	256	Neutral	Positive	Positive	Positive	Neutral
7b	4-F	NHCOCH_3_	128	Positive	Positive	Positive	Positive	Positive
8b	4-Cl	NHCOCH_3_	32	Positive	Positive	Positive	Positive	Positive
9b	3,4-OCH_3_	NHCOCH_3_	> 512	Negative	Neutral	Neutral	Negative	Negative
10b	4-SCH_3_	H	> 512	Negative	Neutral	Neutral	Negative	Negative
11b	2-F	H	256	Neutral	Neutral	Neutral	Neutral	Neutral

The 3D-QSAR model demonstrated a strong predictive capacity, with high R^2^ and Q^2^ values indicating a good correlation between predicted and experimental activities. The steric field was found to have the most significant impact on antibacterial activity, followed by hydrophobic and H-bond donor/acceptor fields. The contour maps generated from the CoMSIA model provided visual insights into the regions of the molecule where modifications could enhance or reduce antibacterial activity.

Compounds **8b** and **11b** were identified as the most active against both *E. coli* and *S. aureus*, with specific structural features contributing to their high activity. The steric and hydrophobic interactions, particularly around the chromene moiety and sulfonamide group, were crucial for enhancing antibacterial properties. The model's predictions were consistent with experimental findings, validating the utility of the 3D-QSAR approach in guiding the design of potent antibacterial agents.

The 3D-QSAR study provided valuable insights into the structure–activity relationships of chromene-sulfonamide hybrids. By accurately predicting the antibacterial activity based on molecular structure, the model aids in the rational design of new compounds with improved efficacy. This computational approach complements experimental assays, reducing the need for extensive trial-and-error synthesis and testing.

## Methods

### In-silico-based assay

3D‑QSAR data set. The data set was gathered against Dihydropteroate Synthase (CHEMBL4032, CHEMBL1075045) for sulfonamides from our previously published study, this study synthesized compounds and reviewed literature^37–44^. The database includes compounds that had the antibacterial effect and sulfonamides functional group. The initial dataset derived from MIC was selected for further investigation on the dataset. Compounds that did not the MIC were omitted. Then 73, and 77 Compounds were gained to assess their antibacterial impact against *S. aureus* and *E. coli*, respectively. All collected data sets are placed in the supplementary file (predicted vs experimental file).

## Conformation minimization, pharmacophore alignment, predictive model calculations

The accumulated CSV format was transformed to SDF format. All extracted compounds were created using the ChemSketch program. Preparation of compounds was accomplished using the LigPrep^45^ module of the Schrodinger Suite, which adds hydrogen atoms, realistically adjusts bond lengths and angles, and corrects any chirality issues, ionization states, tautomers, stereo chemistries, and ring conformations. Partial charges were assigned using the OPLS-2005 force field, and the resulting structures were subjected to minimized energy until their average RMSD reached 0.001 Å. The ionization state was set for pH 7.0 using the ionization tool within Epik^45^. Finally, the compounds were converted to the SDF format.

The crystallized target protein (PDB code: 3TYE) was extracted from the Protein Data Bank (PDB) and separated into protein receptor and ligand references. The structure of the enzyme was pre-processed, minimized, and refined using the Protein Preparation Wizard^46^ implemented in the Schrodinger Suite^47^. This step involved eliminating crystallographic waters, adding missing hydrogens/side chain atoms, and assigning the appropriate charge and protonation state of the receptor structure (for pH 7.0) while considering the appropriate ionization states for the acidic and basic amino acid residues. The prepared macromolecular structure was subjected to energy minimization using the OPLS-2005 force field^48–51^ with a root mean square deviation (RMSD) cut-off value of 0.30 Å to relieve steric clashes among closely spaced residues arising from the addition of hydrogen atoms. Preparation of the reference inhibitor was accomplished using LigPrep.

The quality of molecular alignment is considered as a key factor for the robustness and predictive power of 3D-QSAR models. 3D-pharmacophore alignment was performed to build 3D-QSAR models. In this regard, the bound drug was utilized as a reference ligand to produce 3D-pharmacophore features and subsequently as a bioactive reference conformation, while the target receptor was applied as a protein receptor excluding volume. The PHASE module^45,52,53^ implemented in Schrödinger suite software 2022-02^54^ was used For the structure-based pharmacophore modeling with the default set of seven chemical features: hydrogen bond acceptor (A), hydrogen bond donor (D), hydrophobic contacts (H), negative ionizable group (N), positive ionizable group (P), and aromatic ring (R) to construct the most representative features of the enzyme active sites. The 3D features were generated using Hypothesis Generation for Energy-Optimized Structure Based Pharmacophores considering the excluded volumes within 5 Å of the refined reference inhibitor for the enzyme^55,56^. According to the essential interactions with the key residues of the enzyme involving the inhibitor, pharmacophore features were selected. The resultant pharmacophore features contained the functional groups that were involved in their bioactivity against the mentioned enzyme. The excluded volumes engaged all atoms within 5 Å of the refined inhibitor.

Consequently, the reference conformer was utilized to align the compounds based on the generated 3D-pharmacophore features. The compounds observed biological activities were converted to a logarithmic scale for *E. coli* and* S*. aureus via the formula of pMIC =  − log (MIC).

### 3D-QSAR calculation

CoMSIA^52^ was used to evaluate the steric field and electrostatic field parameters by Lennard Jones and coulombic potential functions, hydrophobic field, hydrogen bond acceptor field, and hydrogen bond donor field parameters^57^. During the calculation process, the compound was placed in the spatial grids, which consisted of many grids with a side length of 2 Å. In this space sp3 hybridized carbon atom was performed as a probe particle to calculate the structural characteristics of the compound^58^. The van der Waals radius of the probe particle and the net charge were set on 1.52 Å + 1.0, respectively. The energy cut-off value was adjusted to 30 kcal mol^−1^ and the default value was adopted for other parameters. Therefore, for the probe particle, in addition to the van der Waals radius of 1.52 Å and the net charge of + 1.0, while hydrophobic parameters, hydrogen bond acceptor parameters, and hydrogen bond donor parameters also should be set to + 1 for calculating the compound characteristics. The attenuation coefficient was configured to 0.3 by default. In the CoMSIA model, Gaussian functions were used to determine the distance between molecule atoms and probe atoms, and the partial least square (PLS) method was applied to analyze CoMSIA models^59,60^.

### Validation of the 3D‑QSAR

The correlation coefficient (R^2^), the cross-validation regression coefficient Q^2^, and the root mean square error (RMSE) were considered to the 3D-QSAR predictive model.

### In silico pharmacokinetics, pharmacodynamics, and toxicity studies using a ADMET™ predictor

The compound's pharmacokinetic and some potential pharmacodynamics parameters were predicted using ADMET Predictor version 9.0 software (Simulations Plus Inc., USA). In this step, drug-like properties quantitative evaluation was assessed, such as permeability, solubility, pKa (a negative logarithmic measure of the acid dissociation constant), lipophilicity, absorption, plasma protein binding, blood–brain barrier penetration, transporters, dermal and ocular penetration, metabolism and drug-drug interaction. A quantitative evaluation of drug-like properties, including pKa, lipophilicity, permeability, solubility, plasma protein binding, absorption, blood–brain barrier permeation, transporters, dermal and ocular penetration, metabolism, and drug-drug interaction was computed. A crucial consideration in the development of effective medications is the safety of the components. A crucial consideration in the development of effective medications is the safety of the components. The compounds pharmacokinetics impact on the hepatocellular enzymes, including gamma-glutamyltransferase (GGT), lactate dehydrogenase (LDH), aspartate transaminase (AST), alanine transaminase (ALT), and alkaline phosphatase (ALP) enzymes were predicted. Furthermore, other parameters such as neurotoxicity, androgen receptor toxicity, allergenicity, mutagenicity, and developmental toxicity were also estimated. The ADMET evaluation was assessed to discover the dose range and define the potential compound's pharmacokinetics and pharmacodynamics in the human body.

### Molecular docking studies

The X-ray diffraction structures for the Dihydropteroate synthase (DHPS) enzyme of both *E. coli* and *S. aureus*, associated with PDB IDs 5AZS, and 3D5K, respectively, were obtained from the Protein Data Bank (https://www.rcsb.org/pdb). To prepare the structures for docking, all water molecules were first removed. Hydrogen atoms were then added at pH 7 using Discovery Studio version 2.5 (Accelrys Inc., San Diego)^61^. The proteins and their ligands were subsequently subjected to energy minimization using the simulation module of Discovery Studio. In the minimization, the conjugate gradient method and the CHARMM force field were employed, until the energy gradient was reduced to below 0.1 cal/Å^62^.

The Autodock Vina module within LigandScout 4.3 (Inte: Ligand GmbH, Vienna, Austria)^62^ was utilized to dock a collection of studied compounds to *E. coli* and *S. aureus* DHPS proteins. For the *E. coli’*s DHPS, a grid map was established with dimensions of 22 × 22 × 22 points and a grid spacing of 1.0 Å. The center of the grid box was positioned at coordinates 38.987 Å (x-axis), 4.272 Å (y-axis), and 8.749 Å (z-axis).

For the DHPS protein of S. aureus, a grid map was configured, maintaining the 22 × 22 × 22 point dimensions and a grid spacing of 1.0 Å. The center grid box coordinates were set at 82.803 Å along the x-axis, 124.447 Å along the y-axis, and 11.493 Å along the z-axis.

The default settings of the Lamarckian genetic algorithm were employed, and the number of runs was set at 40 for profile docking for *E. coli’*s DHPS and *S. aureus*’s DHPS. Interaction profiles between selected ligands and protein were analyzed using LigPlot (European Bioinformatics Institute, Hinxton, UK) and Discovery Studio Visualizer (Biovia, Dassault Systèmes, San Diego, CA, USA) software^62–64^.

### Experimental

#### Materials and measurements

All chemicals were purchased from Merck and Fluka chemical companies. melting points obtained with an Amstead Electro Thermal 9200 apparatus and IR spectra were recorded on a PerkinElmer VIR spectrophotometer. NMR spectra were obtained on a Bruker 400 MHz FT spectrometer (^1^HNMR at 250 Hz, ^13^CNMR at 63 Hz for 4*H*-chromenes, and, ^1^HNMR at 400 Hz, ^13^CNMR at 100 Hz for chromene-sulfonamides) in DMSO‑d_6_ using TMS as an internal standard.

#### General procedure for synthesis of 4H-chromene-3-carbonitrile derivatives

Synthesis of 4*H*-chromene-3-carbonitrile derivatives carried out as reported in the literature with minor modification^65,66^. 0.25 mmol of triethylamine were added to a mixture of substituted benzaldehyde (1 mmol), malononitrile (1 mmol), and 3-aminophenol (1 mmol) in absolute ethanol (5 ml) at room temperature. Then the reaction mixture was refluxed for 2–3 h. The progress of the reaction was monitored by TLC. After completion of the reaction, the reaction mixture was filtrated and the obtained precipitate was washed with cold ethanol. All the obtained products were pure, but if purification is needed, the recrystallization method from ethanol can be used.

#### General procedure for synthesis of chromene-sulfonamides

The synthesized chromene derivatives (1mmol) and anhydrous NaHCO_3_ (18 mmol) were ground together into fine powder in a mortar, and aryl benzene sulfonyl chlorides (3 mmol) namely benzene sulfonyl chloride, *p*-methylbenzene sulfonyl chloride, and *p-*acetamido benzene sulfonyl chloride was added and ground at room temperature. The discoloration of the reaction mixture was a sign of the beginning of product formation. Progress and completion of the reaction were monitored by TLC. After the reaction was completed, distilled water (25 ml) was added to the mixture. The precipitate was collected by filtration, washed with additional water, and finally dried at room temperature. All of the products were obtained very pure in good to high yield. The chemical structures of the products were elucidated by IR, ^1^HNMR, and ^13^CNMR spectroscopy and also the melting point.

#### Spectral data of the novel synthesized chromene derivatives

##### 2,7-Diamino-4-(2-methoxyphenyl)-4H-chromene-3-carbonitrile (2a)

M.p = 217–218 °C; R_*f*_ = 0.63 (70% ethyl acetate, 30% *n*-hexane); IR (KBr, cm^−1^) = 3445, 3365, 3312, 3189 (NH_2_), 2188 (CN), 1633, 1574 (C=C); ^1^H NMR (250 MHz, DMSO) δ 3.68 (d, *J* = 7.1 Hz, 3H), 4.47 (s, 1H), 5.20 (s, 2H), 6.07–6.37 (m, 2H), 6.45–6.94 (m, 5H), 7.06 (d, *J* = 8.0 Hz, 2H); ^13^C NMR (63 MHz, DMSO) δ 36.8, 52.5, 54.3, 97.5, 108.0, 108.6, 111.4, 115.0, 118.5, 125.9, 126.9, 129.4, 136.4, 146.2, 146.4, 155.4, 157.8.

##### 2,7-Diamino-4-(3,4-dimethoxyphenyl)-4H-chromene-3-carbonitrile (3a)

M.p = 194–196 °C; R_*f*_ = 0.50 (70% ethyl acetate, 30% *n*-hexane); IR (KBr, cm^−1^) = 3443, 3416, 3361, 3189 (NH_2_), 2201(CN), 1652, 1577 (C=C); ^1^H NMR (250 MHz, DMSO) δ 3.69 (s, 6H), 4.48 (s, 1H), 5.20 (s, 2H), 6.16–6.34 (m, 2H), 6.60–6.92 (m, 6H); ^13^C NMR (63 MHz, DMSO) δ 37.0, 53.0, 53.1, 54.2, 97.5, 107.9, 108.6, 108.8, 109.5, 111.2, 116.9, 118.5, 126.9, 136.8, 145.0, 146.1, 146.4, 157.9.

##### 2,7-Diamino-4-(3-phenoxyphenyl)-4H-chromene-3-carbonitrile (***6a***)

M.p = 185–186 °C C; R_*f*_ = 0.78 (70% ethyl acetate, 30% *n*-hexane); IR (KBr, cm^−1^) = 3450, 3372, 3317, 3190 (NH_2_), 2190 (CN), 1634, 1574 (C=C); ^1^H NMR (400 MHz, DMSO) δ 4.56 (s, 1H), 5.28 (s, 2H), 6.22 (d, J = 2.2 Hz, 1H), 6.31 (dd, J = 8.3, 2.2 Hz, 1H), 6.65–6.70 (m, 1H), 6.78–6.87 (m, 4H), 6.94 (dt, J = 7.8, 1.2 Hz, 1H), 6.98– 7.04 (m, 2H), 7.15 (tt, J = 7.3, 1.1 Hz, 1H), 7.31 (t, J = 7.9 Hz, 1H), 7.35–7.43 (m, 2H); ^13^C NMR (101 MHz, DMSO) δ 39.5, 56.0, 99.8, 109.6, 111.1, 116.2, 117.6, 118.5, 120.8, 122.4, 123.4, 129.3, 130.0, 130.1, 148.9, 148.9, 149.0, 156.4, 156.6, 160.4.

##### 2,7-Diamino-4-(4-(methylthio) phenyl)-4H-chromene-3-carbonitrile (***8a***)

M.p = 247–248 °C; R_*f*_ = 0.52 (70% ethyl acetate, 30% *n*-hexane); IR (KBr, cm^−1^) = 3446, 3364, 3313, 3188 (NH_2_), 2188 (CN), 1632, 1573 (C=C); ^1^H NMR (250 MHz, DMSO) δ 2.42 (s, 3H), 4.48 (s, 1H), 5.21 (s, 2H), 6.12–6.33 (m, 2H), 6.59 (d, J = 8.1 Hz, 1H), 6.75 (s, 2H), 7.03–7.23 (m, 4H); ^13^C NMR (63 MHz, DMSO) δ 12.3, 37.0, 53.8, 97.4, 107.4, 108.6, 118.4, 123.8, 125.5, 126.9, 133.5, 141.1, 146.3, 146.4, 157.9.

##### 2,7-Diamino-4-(2-fluorophenyl)-4H-chromene-3-carbonitrile (***9a***)

M.p = 232–233 °C; R_*f*_ = 0.70 (70% ethyl acetate, 30% *n*-hexane); IR (KBr, cm^−1^) = 3427, 3350, 3310, 3156 (NH_2_), 2195 (CN), 1647, 1589 (C=C); ^1^H NMR (250 MHz, DMSO) δ 4.82 (s, 1H), 5.25 (s, 2H), 6.10–6.41 (m, 2H), 6.62 (d, J = 8.1 Hz, 1H), 6.82 (s, 2H), 7.15 (dq, J = 14.2, 8.1 Hz, 4H); ^13^C NMR (63 MHz, DMSO) δ 31.7, 52.3, 97.5, 106.4, 108.7, 112.9, 113.3, 118.2, 122.2, 126.0, 126.2, 126.5, 127.3, 130.4, 130.6, 146.5, 146.7, 155.4, 158.4, 159.3.

#### Spectral data of the synthesized chromene sulfonamide hybrids

##### N-[2-amino-3-cyano-4-(4-methoxy-phenyl)-4H-chromene-7-yl]-4-methyl-benzene sulfonamide (**1b**)

Yellow solid; m.p = 206–209 °C; R_*f*_ = 0.68 (70% EtOAc, 30% *n*-hexane); FTIR (KBr)/ν_max_ (cm^−1^) = 3478, 3402 (NH_2_), 3149 (N–H sulfonamide), 2196 (CN), 1649, 1579 (C = C), 1336, 1159 (SO_2_); ^1^HNMR (400 MHz, DMSO) δ (ppm) = 2.18 (s, 3H_CH3_); 3.82 (s, 3H_OCH3_), 4.68 (s, 1H_CH(4)_), 6.64–7.13 (m, 7H_Ar,_ 2H_NH2_), 7.79 (s, 4H_Ar_), 10.42 (s, 1H_NH-SO2_); ^13^CNMR (100 MHZ, DMSO) δ (ppm) = 21.0, 39.4, 55.0, 55.8, 106.4, 114.0, 115.8, 119.1, 120.4, 126.6, 128.4, 129.8, 130.0, 136.4, 137.5, 137.8, 143.5, 148.3, 158.0, 159.9. Anal. Calcd. for C_24_H_21_N_3_O_4_S, %: C, 64.42; H, 4.73; N, 9.39. Found, %: C, 64.56; H, 4.62; N, 9.51.

##### N-[2-amino-3-cyano-4-(2-methoxy-phenyl)-4H-chromene-7-yl]-4-methyl-benzene sulfonamide (**2b**)

Yellow solid; m.p = 180–183 °C; R_*f*_ = 0.68 (70% EtOAc, 30% *n*-hexane); FTIR (KBr)/ν_max_ (cm^−1^) = 3470, 3403 (NH_2_), 3155 (N–H sulfonamide), 2195 (CN), 1649, 1581 (C=C), 1335, 1159 (SO_2_); ^1^HNMR (400 MHz, DMSO) δ (ppm) = 2.45 (s, 3H_CH3_), 3.82 (s, 3H_OCH3_), 4.69 (s, 1H_CH(4)_), 6.88 (d, J = 7.3 Hz, 2H_Ar_), 6.96 (d, J = 7.5 Hz, 3H_Ar_), 7.03 (s, 2H_NH2_), 7.15 (d, J = 8.0 Hz, 2H_Ar_), 7.47 (d, J = 8.1 Hz, 2H_Ar_), 7.75 (d, J = 7.9 Hz, 2H_Ar_), 10.54 (s, 1H_NH-SO2_); ^13^CNMR (100 MHZ, DMSO) δ (ppm) = 21.4, 40.6, 55.5, 56.6, 106.9, 114.5, 116.3, 119.6, 120.9, 126.0, 127.1, 128.6, 128.9, 130.3, 130.5, 136.9, 138.0, 138.3, 144.0, 148.8, 158.5, 160.4. Anal. Calcd. for C_24_H_21_N_3_O_4_S, %: C, 64.42; H, 4.73; N, 9.39. Found, %: C, 64.52; H,4.67; N, 9.54.

##### N-[2-amino-3-cyano-4-(3,4-dimethoxy-phenyl)-4H-chromene-7-yl]-4-methyl-benzene sulfonamide (**3b**)

Yellow solid; m.p = 127–130 °C; R_*f*_ = 0.51 (70% EtOAc, 30% *n*-hexane); FTIR (KBr)/ν_max_ = 3449, 3346 (NH_2_), 3246 (N–H sulfonamide), 2190 (CN), 1650, 1605 (C=C), 1332, 1157 (SO_2_); ^1^HNMR (400 MHz, DMSO) δ (ppm) = 2.47 (s, 3H_CH3_), 3.80 (s, 3H_OCH3_), 3.83 (s, 3H_OCH3_), 4.80 (s, 1H_CH(4)_), 6.74–7.05 (m, 6H_Ar_, 2H_NH2_), 7.48 (d, *J* = 7.9 Hz, 2H_Ar_), 7.76 (d, *J* = 7.8 Hz, 2H_Ar_), 10.56 (s, 1H_NH-SO2_); ^13^CNMR (100 MHZ, DMSO) δ (ppm) = 20.9, 39.2, 55.3, 55.4, 56.0, 106.4, 111.1, 111.9, 115.8, 118.9, 119.3, 120.4, 126.6, 129.8, 130.0, 136.3, 137.5, 138.2, 143.5, 147.6, 148.2, 148.6, 159.9. Anal. Calcd. for C_25_H_23_N_3_O_5_S, %: C, 62.88; H, 4.85; N, 8.80. Found, %: C, 63.01; H,4.93; N, 8.68.

##### N-[2-amino-3-cyano-4-(4-chloro-phenyl)-4H-chromene-7-yl]-4-methyl-benzene sulfonamide (**4b**)

Orange solid; m.p = 179–182 °C; R_*f*_ = 0.75 (70% EtOAc, 30% *n*-hexane; FTIR (KBr)/ν_max_ (cm^–1^) = 3418, 3331 (NH_2_), 3210 (N–H sulfonamide), 2195 (CN), 1652, 1585 (C=C), 1329, 1161 (SO_2_); ^1^HNMR (400 MHz, DMSO) δ (ppm) = 2.34 (s, 3H_CH3_), 4.69 (s, 1H_CH(4)_), 6.75–6.81 (m, 2H_Ar_), 6.87 (d, *J* = 8.3 Hz, 1H_Ar_), 7.03 (s, 2H_NH2_), 7.16 (d, *J* = 8.1 Hz, 2H_Ar_), 7.36 (d, *J* = 7.9 Hz, 4H_Ar_), 7.64 (d, *J* = 8.0 Hz, 2H_Ar_), 10.48 (s, 1H_NH-SO2_); ^13^CNMR (100 MHZ, DMSO) δ (ppm) = 21.4, 39.6, 56.0, 106.9, 116.3, 118.7, 120.7, 127.1, 129.1, 129.8, 130.3, 130.5, 131.9, 136.9, 138.3, 144.0, 145.2, 148.8, 160.5. Anal. Calcd. for C_23_H_18_ClN_3_O_3_S, %: C, 61.13; H, 4.01; N, 9.30. Found, %: C, 61.35; H, 3.97; N, 9.43.

##### N-[4-(2-amino-3-cyano-4-phenyl-4H-chromen-7-yl sulfamoyl)-phenyl]-acetamide (**5b**)

Yellow solid; m.p = 240–243 °C; R_*f*_ = 0.42 (90% EtOAc, 10% *n*-hexane); FTIR (KBr)/ν_max_ = 3426, 3343(NH_2_), 3130 (N–H sulfonamide), 3065 (N–H amide), 2189 (CN), 1674 (C=O), 1655, 1590 (C=C), 1336, 1159 (SO_2_); ^1^HNMR (400 MHz, DMSO) δ (ppm) = 2.18 (s, 3H_CH3_), 4.75 (s, 1H_CH (4)_), 6.88 (d, *J* = 8.3 Hz, 2H_Ar_), 6.99 (d, *J* = 8.2 Hz, 1H_Ar_), 7.07 (s, 2H_NH2_), 7.23 (d, *J* = 7.6 Hz, 2H_Ar_), 7.31 (t, *J* = 7.2 Hz, 1H_Ar_), 7.41 (t, *J* = 7.5 Hz, 2H_Ar_), 7.80 (q, *J* = 8.7 Hz, 4H_Ar_), 10.45 (s, 1H_NH-SO2_), 10.49 (s, 1H_NH amide_); ^13^CNMR (100 MHZ, DMSO) δ (ppm) = 24.6, 40.1, 56.8, 107.1, 116.4, 116.5, 119.1, 120.8, 120.9, 127.3, 127.9, 128.4, 129.1, 130.5, 133.3, 143.7, 146.3, 148.9, 160.5, 169.5. Anal. Calcd. for C_24_H_20_N_4_O_4_S, %: C, 62.60; H, 4.38; N, 12.17. Found, %: C, 62.72; H, 4.26; N, 12.27.

##### N-[4-[2-amino-3-cyano-4-(3-phenoxy-phenyl)-4H-chromene-7-yl sulfamoyl]-phenyl}-acetamide (**6b**)

Yellow solid; m.p = 141–144 °C; R_*f*_ = 0.48 (90% EtOAc, 10% *n*-hexane); FTIR (KBr)/ν_max_ = 3447, 3357 (NH_2_), 3213 (N–H sulfonamide), 3063 (N–H amide), 2190 (CN), 1691 (C = O), 1649, 1590 (C = C), 1322, 1156 (SO_2_); ^1^HNMR (400 MHz, DMSO) δ (ppm) = 2. 27 (s, 3H_CH3_), 4. 87 (s, 1H_CH (4)_), 6. 95 -7.21 (m, 7H_Ar_, 2H_NH2_), 7. 29–7. 40 (m, 2H_Ar_), 7. 47–7.64 (m, 3H_Ar_), 7. 91 (q, *J* = 8. 7 Hz, 4H_Ar_), 10. 55 (s, 1H_NH-SO2_), 10. 61 (s, 1H_NH-amide_); ^13^CNMR (100 MHZ, DMSO) δ (ppm) = 24.1, 39.6, 64.4, 106.5, 115.9, 116.4, 117.5, 118.3, 118.6, 119.3, 120.1, 122.3, 123.5, 124.6, 127.8, 130.0, 130.3, 132.7, 137.7, 143.3, 148.0, 148.2, 156.0, 156.7, 160.1, 169.0. Anal. Calcd. for C_30_H_24_N_4_O_5_S, %: C, 65.21; H, 4.38; N, 10.14. Found, %: C, 65.43; H, 4.3; N, 10.02.

##### N-[4-[2-amino-3-cyano-4-(4-fluoro-phenyl)-4H-chromene-7-yl sulfamoyl]-phenyl}-acetamide (**7b**)

Yellow solid; m.p = 230–233 °C; R_*f*_ = 0.45 (90% EtOAc, 10% *n*-hexane); FTIR (KBr)/ν_max_ = 3433, 3326 (NH_2_), 3271 (N–H sulfonamide), 3210 (N–H amide), 2192 (CN), 1674 (C=O), 1651, 1594 (C=C), 1326, 1157 (SO_2_); ^1^HNMR (400 MHz, DMSO) δ (ppm) = 2.27 (s, 3H_CH3_), 4.88 (s, 1H_CH(4)_), 6.97 (d, *J* = 7.8 Hz, 2H_Ar_), 7.06 (d, *J* = 8.2 Hz, 1H_Ar_), 7.18 (s, 2H_NH2_), 7.30–7.38 (m, 4H_Ar_), 7.89 (q, *J* = 8.9 Hz, 4H_Ar_), 10.54 (s, 1H_NH-SO2_), 10.59 (s, 1H_NH-amide_); ^13^CNMR (100 MHZ, DMSO) δ (ppm) = 24.1, 39.0, 55.8, 106.6, 115.3, 115.5, 116.0, 118.5, 118.6, 120.3, 127.9, 129.2, 129.3, 130.0, 132.6, 137.7, 141.9, 142.0, 143.3, 148.3, 159.8, 159.9, 162.2, 169.1. Anal. Calcd. for C_24_H_19_FN_4_O_4_S, %: C, 60.24; H, 4.00; N, 11.71. Found, %: C, 60.29; H, 4.11; N, 11.59.

##### N-[4-[2-amino-3-cyano-4-(4-chloro-phenyl)-4H-chromene-7-yl sulfamoyl]-phenyl}-acetamide (**8b**)

Yellow solid; m.p = 227–230 °C; R_*f*_ = 0.45 (90% EtOAc, 10% *n*-hexane); FTIR (KBr)/ν_max_ = 3440, 3367 (NH_2_), 3320 (N–H sulfonamide), 3275 (N–H amide), 2170 (CN), 1674 (C=O), 1648, 1593 (C=C), 1326, 1157 (SO_2_); ^1^HNMR (400 MHz, DMSO) δ (ppm) = 2.19 (s, 3H_CH3_), 4.81 (s, 1H_CH(4)_), 6.89 (d, *J* = 7.3 Hz, 2H_Ar_), 6.98 (d, *J* = 7.4 Hz, 1H_Ar_), 7.13 (s, 2H_NH2_), 7.26 (d, *J* = 8.2 Hz, 2H_Ar_), 7.48 (d, *J* = 7.7 Hz, 2H_Ar_), 7.81 (q, *J* = 8.7 Hz, 4H_Ar_), 10.46 (s, 1H_NH-SO2_), 10.53 (s, 1H_NH-amide_); ^13^CNMR (100 MHZ, DMSO) δ (ppm) = 24.1, 39.1, 55.9, 106.6, 116.0, 118.1, 118.6, 120.2, 127.9, 128.6, 129.3, 130.0, 131.4, 132.6, 137.8, 143.3, 144.7, 148.3, 160.0, 169.1. Anal. Calcd. for C_24_H_19_ClN_4_O_4_S %: C, 58.24; H, 3.87; N, 11.32. Found, %: C, 58.34; H, 3.98; N, 11.43.

##### N-[4-[2-amino-3-cyano-4-(3,4-dimethoxy-phenyl)-4H-chromene-7-yl sulfamoyl]-phenyl}-acetamide (**9b**)

Yellow solid; m.p = 235–238 °C; R_*f*_ = 0.27 (90% EtOAc, 10% n-hexane); FTIR (KBr)/ν_max_ = 3412, 3322 (NH_2_), 3265 (N–H sulfonamide), 3210 (N–H amide), 2193 (CN), 1695 (C=O), 1651, 1592(C=C), 1314, 1161 (SO_2_); ^1^HNMR (400 MHz, DMSO) δ (ppm) = 2.24 (s, 3H_CH3_), 3.72–3.91 (m, 6H(_2OCH3_)), 4.73 (s, 1H_CH(4)_), 6.78–7.06 (m, 6H_Ar_, 2H_NH2_), 7.85 (s, 4H_Ar_), 10.46 (s, 2H_NHamide, NHSO2_); ^13^CNMR (100 MHZ, DMSO) δ (ppm) = 24.1, 39.4, 55.3, 55.4, 56.1, 106.4, 111.1, 111.9, 116.2, 118.0, 118.5, 119.3, 120.5, 127.7, 129.7, 133.8, 138.3, 138.9, 142.8, 147.5, 148.2, 148.6, 159.9, 169.0. Anal. Calcd. for C_26_H_24_N_4_O_6_S%: C, 59.99; H, 4.65; N, 10.76. Found, %: C, 60.08; H, 4.82; N, 10.87.

##### N-(2-amino-3-cyano-4-(4-(methylthio)phenyl)-4H-chromen-7-yl)benzenesulfonamide (**10b**)

Orange solid; m.p = 135–138 °C; R_*f*_ = 0.45 (50% EtOAc, 50% n-hexane); FTIR (KBr)/ν_max_ = 3472, 3360 (NH_2_), 3185 (N–H sulfonamide), 2182 (CN), 1643, 1582 (C=C), 1330, 1155 (SO2); ^1^HNMR (400 MHz, DMSO) δ (ppm) = 2.46 (s, 3H_CH3_), 4.38 (s, 1H_CH(4)_), 6.84–6.92 (m, 3H_Ar_), 7.08 (s, 2H_NH2_), 7.20 (s, 2H_Ar_), 7.56–7.66 (m, 4H_Ar_), 7.78 (s, 3H_Ar_), 10.55 (s, 1H_NH-SO2_); ^13^CNMR (100 MHZ, DMSO) δ (ppm) = 14.7, 39.1, 55.9, 106.7, 116.0, 118.8, 120.3, 126.2, 126.6, 128.0, 129.4, 130.0, 133.2, 136.5, 137.5, 139.3, 142.4, 148.3, 160.0. Anal. Calcd. for C_23_H_19_N_3_O_3_S_2_%: C, 61.45; H, 4.26; N, 9.35. Found, %: C, 61.57; H, 4.13; N, 9.44.

##### N-(2-amino-3-cyano-4-(2-fluorophenyl)-4H-chromen-7-yl) benzenesulfonamide (**11b**)

Yellow solid; m.p = 122–125 °C; R_*f*_ = 0.54 (50% EtOAc, 50% n-hexane); FTIR (KBr)/ν_max_ = 3468, 3354 (NH_2_), 3154 (N–H sulfonamide), 2194 (CN), 1643, 1585 (C=C), 1329, 1156 (SO_2_); ^1^HNMR (400 MHz, DMSO) δ (ppm) = 4.99 (s, 1H_CH(4)_), 6.84–6.99 (m, 3H_Ar_), 7.08 (s, 2H_NH2_), 7.23 (s, 3H_Ar_), 7.37 (s, 1H_Ar_), 7.62–7.73 (m, 3H_Ar_), 7.85 (d, *J* = 7.3 Hz, 2H_Ar_), 10.62 (s, 1H_NH-SO2_); ^13^CNMR (100 MHZ, DMSO) δ (ppm) = ^13^CNMR (100 MHZ, DMSO) δ (ppm) = 34.4, 54.3, 106.6, 115.7, 115.9, 116.0, 117.6, 120.1, 124.7, 124.8, 126.5, 129.0, 129.1, 129.4, 129.6, 129.8, 129.9, 131.9, 132.0, 133.1, 137.7, 139.2, 148.6, 158.6, 160.3, 161.1. Anal. Calcd. for C_22_H_16_FN_3_O_3_S%: C, 62.70; H, 3.83; N, 9.97. Found, %: C, 62.84; H, 3.76; N, 10.08.

All of the spectral data were placed in the supplementary file (Figs. S3–S54).

### Antibacterial activity evaluation

A broth microdilution method was utilized to explore the minimum inhibitory concentrations (MIC) of all the newly synthesized compounds against *E. coli* ATCC 25922 and *S. aureus* ATCC 25923. Test compounds were prepared using dimethyl sulfoxide (DMSO) as a stock solution, followed by serial dilutions in Mueller Hinton broth containing up to one percent DMSO. At the end, a diluted bacterial suspension from a 0.5 McFarland tube was added to all the wells (the final volume of each well was 1 × 10^5^ CFU/ml). In the growth control, a culture medium with bacteria was used as a positive control, and a culture medium without bacteria was used as a negative control (sterility control). The MIC was the lowest concentration of the synthesized compounds that produced no growth on the plate^2^. Sulfisoxazole, functioning as a sulfonamide antibiotic, and gentamicin were employed in a comparative analysis of antibacterial activity, cytotoxicity, and apoptosis assessment.

### Cytotoxicity assay by MTT

MTT was applied to evaluate the cytotoxicity of the newly synthesized compounds against fibroblast L929 cells (Pasteur Institute, Tehran, Iran). Briefly, 100 μl of cell suspension (1 × 10^4^ cells/well) in RPMI 1640 medium, as seeded in 96-well microplates and incubated for 24 h (37 °C, 5% CO_2_ air humidified). After removing the old media, 100 µl of each of the newly synthesized compounds was added to each well at different concentrations. The plates were incubated for another 24 h in the same condition. To determine the positive and negative controls, doxorubicin and DMSO were used. Cell survival was determined by adding 10 µL of an MTT solution (5 mg/ml in PBS) to each well and incubating at 37 °C for 3 h. The previous MTT-containing media was then removed, and 100 µL DMSO was pipetted into each well to dissolve the produced formazan crystals. Using an ELISA plate reader (Startfix-2100, Awareness, USA), absorbance was measured at 570 nm. By plotting the percentage of cytotoxicity versus concentration of the produced compounds, the concentrations that inhibit half of the cell population (IC_50_) were determined. A mean and standard deviation (SD) are used to represent the results^67^. The percentage cell viability was calculated using the formula:

$$\text{Viability}=\frac{\text{Absorbance \, of \, treated \, cells}-\text{background \, absorbance }(\text{b})}{\text{Absorbance \, of \, untreated \, cells }(\text{c})-\text{background \, absorbance }(\text{b})}\times 100$$

where (b) = blank, and (c) = control.

### Apoptosis evaluation by an Annexin V binding assay

To detect apoptosis, the Annexin V binding assay was used. The assay was performed according to the manufacturer's instructions using a FITC Annexin V Apoptosis Detection Kit with PI from BioLegend® (San Diego, California). To conduct this test, the fibroblast L929 cell line (Pasteur Institute, Tehran, Iran) was treated with various concentrations of the newly synthesized compounds and then incubated for 24 h at 37 °C with 5% CO_2_. Subsequently, each well's supernatant was removed and the cells were washed with PBS. Each well was then filled with 500 μl of Annexin-binding buffer (10 mM HEPES, 140 mM NaCl, and 2.5 mM CaCl_2_, pH 7.4) and 10 μl of Annexin V and PI. For 15 min, the plates were incubated at room temperature and kept away from light. After incubation, using a BD FACSCalibur flow cytometer (Biosciences, San Jose, CA, USA), the cells were examined after incubation. FlowJo software (version 10.5.3, TreeStar Incorporated, Ashland, OR) was used to analyze flow cytometry data.

## Conclusion

The 3D-QSAR analysis showed that the predictive model had good prediction ability and statistically is reliable. The contour maps also indicated the relationship between the structure and activity of small molecules. Our study would provide informative experimental and theoretical guidance for the future design of chromene-sulfonamide hybrid inhibitors.

Interestingly our experimental results supported and conflicted with ADMET prediction about compounds carcinogenicity of our compounds. ADMET predictor assessments are set up on the QSAR of its databases, hence it sounded like this program needed to improve the quality of these types of evaluations for its software.

In conclusion, several chromene-sulfonamide hybrids were designed and synthesized as antibacterial agents under solvent-free conditions. The used method for the synthesis of these compounds had advantages such as high yield and purity, no need to use toxic solvents and base, simple work-up just by adding water to the reaction medium, and filtration to remove the base and room temperature. Also, according to investigations and antibacterial results obtained on chromene-sulfonamide compounds, among all the compounds, two compounds *N*-{4-[2-amino-3-cyano-4-(3-phenoxy-phenyl)-4*H*-chromene-7-yl sulfamoyl]-phenyl}z-acetamide (compound **6b**) and N-[4-[2-amino-3-cyano-4-(4-chloro-phenyl) -4*H*-chromene-7-yl sulfamoyl]-phenyl}-acetamide (compound **8b**) showed more antibacterial activity than other chromene-sulfonamide derivatives against both Gram-positive and negative bacteria, so it can be concluded that chromene compounds that had phenoxy and chlorine groups showed better antibacterial activities against Gram-positive and Gram-negative bacteria. In addition, MTT results demonstrated that compounds **1b**, **4b**, **6b**, and **8b** have a greater cytotoxicity effect on the fibroblast L929 cell line than other compounds. Several variables must be considered when evaluating a substance's toxicity, including its chemical structure, concentration, and the experimental system. Despite safety concerns, greater cytotoxicity may be desirable in some circumstances, such as when developing anticancer drugs or antimicrobial products. Further investigation into the compounds' safety profile would involve assessing their effects on additional cell lines and doing animal tests to evaluate their systemic toxicity.

### Supplementary Information

Supplementary Information.

## Data Availability

All data generated or analyzed during this study are included in this published article [and its supplementary information files].
