# Supplementary material for: Evaluation of antibacterial, cytotoxicity, and apoptosis activity of novel chromene-sulfonamide hybrids synthesized under solvent-free conditions and 3D-QSAR modeling studies
Source: Sci Rep. 2024 Jun 5;14:12878. doi: 10.1038/s41598-024-63535-5 (PMC11150261; doi:10.1038/s41598-024-63535-5)
Supplement: Supplementary file 1 — Supplementary Information. [file 41598_2024_63535_MOESM1_ESM.docx]

**SUPPLEMENTARY INFORMATION**

**Evaluation of Antibacterial, Cytotoxicity, and Apoptosis Activity of Novel Chromene-Sulfonamide Hybrids Synthesized Under Solvent-Free Conditions and 3D-QSAR Modeling Studies**

Shakila Ghomashi ^a^, Reihane Ghomashi ^b^, Mohammad Sadegh Damavandi ^c d^, Zeynab Fakhar ^e^, Seyedeh Yasaman Mousavi ^f^, Azhar Salari-Jazi ^g h*^, Sajjad Gharaghani ^e*^, Ahmad Reza Massah ^i, j^*

^a^ Department of Medicinal Chemistry, Shahreza Branch, Islamic Azad University, P.O. Box 311-86145, Shahreza, Isfahan, Iran

^b^ Department of Medicinal Chemistry, School of Pharmacy and Pharmaceutical Sciences, Isfahan University of Medical Sciences, Isfahan, 8174673461, Iran

^c^ Department of Microbiology and Virology, Faculty of Medicine, Mashhad University of Medical Sciences, Mashhad, Iran

^d^ Antimicrobial Resistance Research Center, Mashhad University of Medical Sciences, Mashhad, Iran

^e^ Laboratory of Bioinformatics and Drug Design (LBD), Institute of Biochemistry and Biophysics, University of Tehran, Tehran, Iran

^f^ Department of Animal Biology, Faculty of Natural Sciences, University of Tabriz, Tabriz, Iran

^g^ Faculty of Medicine and Health Technology, Tampere University, Tampere, Finland

^h^ Department of Drug Development and Innovation, Behban Pharmed Lotus, Tehran, Iran

^i^ Department of Chemistry, Shahreza Branch, Islamic Azad University, P.O. Box 311-86145, Shahreza, Isfahan, Iran

^j^ Department of Chemistry, Brock University, St. Catharines, Ontario, Canada

Corresponding author email: azhar_sallari@yahoo.com (A. Salari-jazi), ORCID ID:0000-0001-6591-1815

Corresponding author email: [s.gharaghani@ut.ac.ir](mailto:s.gharaghani@ut.ac.ir) (S. Gharaghani), ORCID ID: 0000-0001-5468-4258

Corresponding author email: [massah@iaush.ac.ir](mailto:massah@iaush.ac.ir); vf_arezamassah@brocku.ca (A.R. Massah), ORCID ID: 0000-0001-8843-3190

**Table S1**. The ADMET Predictor solubility analyses of compounds

| Solubility Characteristics | Compound | | | | | | | | | | | |
| --- | --- | --- | --- | --- | --- | --- | --- | --- | --- | --- | --- | --- |
|  |  | **2b** | **3b** | **5b** | **1b** | **4b** | **6b** | **7b** | **8b** | **9b** | **10b** | **11b** |
|  | **S+Sw ^1^** | 0.00029 | 0.00043 | 0.00019 | 0.00031 | 0.00010 | 0.000013 | 0.00011 | 0.00008 | 0.00036 | 0.00049 | 0.00025 |
|  | **S+pH_Satd ^2^** | 6.872 | 6.852 | 6.927 | 6.883 | 6.943 | 6.997 | 6.945 | 6.962 | 6.892 | 6.849 | 6.832 |
|  | **S+S_Intrins ^3^** | 0.00026 | 0.00040 | 0.00017 | 0.00028 | 0.000091 | 0.000012 | 0.000098 | 0.000075 | 0.00033 | 0.00046 | 0.00021 |
|  | **SupSat ^4^** | SupSat (96%) | SupSat (96%) | SupSat (89%) | SupSat (96%) | SupSat (96%) | SupSat (84%) | SupSat (89%) | SupSat (89%) | SupSat (89%) | SupSat (96%) | SupSat (96%) |
|  | **SolFactor ^5^** | 9788.007 | 8040.201 | 12647.557 | 9381.421 | 18507.113 | 66699.958 | 18266.708 | 21731.553 | 9388.731 | 7157.537 | 10685.645 |
|  | **S+S_pH ^6^** | 0.00035 | 0.00051 | 0.00022 | 0.00036 | 0.00012 | 0.000014 | 0.00013 | 0.000098 | 0.00041 | 0.00057 | 0.00034 |
|  | **FaSSIF ^7^**  **(mg/ml)** | 0.013 | 0.016 | 0.011 | 0.013 | 0.010 | 0.003 | 0.008 | 0.006 | 0.010 | 0.020 | 0.024 |
|  | **FeSSIF ^8^**  **(mg/ml)** | 0.143 | 0.119 | 0.110 | 0.133 | 0.156 | 0.074 | 0.099 | 0.090 | 0.081 | 0.161 | 0.229 |
|  | **FaSSGF ^9^**  **(mg/ml)** | 0.009 | 0.008 | 0.011 | 0.010 | 0.007 | 0.001 | 0.008 | 0.006 | 0.007 | 0.019 | 0.014 |

1. S+Sw: Native solubility
2. S+pH_Satd: Native pH at saturation in pure water
3. S+S_Intrins: Intrinsic solubility in pure water
4. SupSat: Supersaturation ratio
5. SolFactor: Salt solubility factor
6. S+S_pH: Water solubility at user-specified pH
7. FaSSIF: fasted state simulated intestinal fluid
8. FeSSIF: fed state simulated intestinal fluid
9. FaSSGF: fasted state simulated gastric fluid

**Table S2**. The ADMET Predicter permeability analyses of compounds

|  | Permeability Characteristics | | | | | | |
| --- | --- | --- | --- | --- | --- | --- | --- |
| Compounds |  | **S+Peff ^1^**  **(cm/s×10^-4^)** | **S+MDCK ^2^** | **Perm_Cornea ^3^**  **(cm/s×10^7^)** | **BBB_Filter ^4^** | **LogBB ^5^** | **Perm_Skin ^6^**  **(cm/s×107)** |
|  | **2b** | 0.652 | 32.279 | 130.925 | Low (33%) | -0.957 | 1.817 |
|  | **3b** | 0.508 | 26.735 | 103.209 | Low (63%) | -1.21 | 1.658 |
|  | **5b** | 0.348 | 25.484 | 156.282 | High (78%) | -0.98 | 2.565 |
|  | **1b** | 0.613 | 26.2 | 134.522 | High (81%) | -1.02 | 1.913 |
|  | **4b** | 0.804 | 47.633 | 187.346 | High (93%) | -0.92 | 3.23 |
|  | **6b** | 0.301 | 24.009 | 185.159 | Low (67%) | -1.025 | 4.79 |
|  | **7b** | 0.331 | 25.433 | 166.896 | High (80%) | -0.86 | 3.424 |
|  | **8b** | 0.32 | 35.491 | 165.817 | High (86%) | -0.915 | 5.834 |
|  | **9b** | 0.221 | 22.726 | 97.203 | Low (67%) | -1.191 | 2.981 |
|  | **10b** | 0.719 | 24.326 | 125.437 | High (99%) | -1.176 | 1.317 |
|  | **11b** | 0.907 | 37.831 | 174.217 | High (78%) | -0.904 | 1.312 |

1. S+Peff : Human effective permeability- jejunal Peff
2. S+MDCK: MDCK apparent permeability
3. Perm_Cornea: Corneal permeability
4. BBB_Filter: Blood-brain barrier permeation (classification model)
5. LogBB: Blood-brain barrier permeation (regressionmodel)
6. Perm_Skin: Skin permeability

**Table S3**. The ADMET Predicter analyses of several characteristics influencing the solubility and permeability

| Compound | MW  (g/mol) | pka ^1^ | MlogP ^2^ | S+logP ^3^ | S+logD ^4^ | logHLC ^5^  (atm*m^3^/mol) | DiffCoef ^6^  (cm^2^/s×10^-5^) |
| --- | --- | --- | --- | --- | --- | --- | --- |
| 2b |  | 10.89 | 2.242 | 4.352 | 4.228 | -11.997 | 0.604 |
| 3b |  | 10.87 | 1.701 | 4.256 | 4.145 | -11.841 | 0.583 |
| 5b |  | 10.95 | 2.381 | 4.393 | 4.293 | -12.535 | 0.588 |
| 1b |  | 10.91 | 2.242 | 4.284 | 4.178 | -12.057 | 0.604 |
| 4b |  | 10.87 | 3.001 | 4.780 | 4.644 | -12.36 | 0.612 |
| 6b |  | 10.90 | 2.614 | 5.785 | 5.724 | -13.08 | 0.53 |
| 7b |  | 10.89 | 2.483 | 4.682 | 4.553 | -12.231 | 0.585 |
| 8b |  | 10.88 | 2.585 | 4.855 | 4.736 | -12.574 | 0.576 |
| 9b |  | 10.89 | 1.299 | 4.353 | 4.256 | -12.183 | 0.551 |
| 10b |  | 10.96 | 2.522 | 3.955 | 3.861 | -13.068 | 0.609 |
| 11b |  | 10.80 | 2.683 | 4.203 | 4.009 | -12.287 | 0.643 |

1. pka: Multiprotic pKa model
2. MlogP: Moriguchi octanol to water partition coefficient
3. S+logP: octanol-water partition coefficient
4. S+logD: octanol-water distribution coefficient
5. logHLC: air-water partition
6. DiffCoef: diffusion coefficient

**Table S4**. Possible metabolism of compounds by CYP

| CYP types | Effect | Compound |
| --- | --- | --- |
| CYP1A2 | substrate | - |
|  | site of metabolism | - |
|  | inhibitor | 2b, 3b, 5b, 1b, 4b, 6b, 7b, 8b, 9b, 10b, and 11b |
| CYP2A6 | substrate | - |
|  | site of metabolism | - |
| CYP2B6 | substrate | - |
|  | site of metabolism | - |
| CYP2C8 | substrate | 2b, 3b, 5b, 1b, 4b, 6b, 7b, 8b, 9b, 10b, and 11b |
|  | site of metabolism | 2b, 3b, 5b, 1b, 4b, 6b, 7b, 8b, 9b, 10b, and 11b |
| CYP2C9 | substrate | 2b, 3b, 1b, 4b, and 11b |
|  | site of metabolism | 2b, 3b, 1b, 4b, and 11b |
|  | inhibitor | 2b, 3b, 5b, 1b, 4b, 6b, 7b, 8b, 9b, 10b, and 11b |
| CYP2C19 | substrate | - |
|  | site of metabolism | - |
|  | inhibitor | 5b, 7b, and 10b |
| CYP2D6 | substrate | - |
|  | site of metabolism | - |
| CYP2E1 | substrate | - |
|  | site of metabolism | - |
| CYP3A4 | substrate | 2b, 3b, 5b, 1b, 4b, 6b, 7b, 8b, 9b, 10b, and 11b |
|  | site of metabolism | 2b, 3b, 5b, 1b, 4b, 6b, 7b, 8b, 9b, 10b, and 11b |
|  | inhibitor | 2b, 3b, 5b, 1b, 4b, 6b, 7b, 8b, 9b, 10b, and 11b |

**Table S5**. Elimination mechanism of xenobiotic by UGT enzymes

| Compound | | | | | | | | | | | |
| --- | --- | --- | --- | --- | --- | --- | --- | --- | --- | --- | --- |
| UGT type | **2b** | **3b** | **5b** | **1b** | **4b** | **6b** | **7b** | **8b** | **9b** | **10b** | **11b** |
| UGT1A1 | No (99%) | No (98%) | No (99%) | No (98%) | No (97%) | No (98%) | No (98%) | No (97%) | No (98%) | No (99%) | No (99%) |
| UGT1A3 | Yes (58%) | Yes (55%) | Yes (64%) | Yes (58%) | Yes (68%) | Yes (64%) | Yes (64%) | Yes (64%) | Yes (53%) | Yes (58%) | Yes (64%) |
| UGT1A4 | No (75%) | No (75%) | No | No | No (72%) | No | No | No (69%) | No | No | No (75%) |
| UGT1A6 | No (78%) | No (88%) | No (70%) | No (88%) | No (76%) | No (73%) | No (76%) | No (75%) | No (85%) | No (68%) | No (70%) |
| UGT1A8 | No | No (83%) | No (76%) | No (86%) | No (80%) | No | No | No (66%) | No | No (86%) | No (80%) |
| UGT1A9 | No (86%) | No (86%) | No (96%) | No (86%) | No (81%) | No (96%) | No (96%) | No (81%) | No (96%) | No (96%) | No (86%) |
| UGT1A10 | No (53%) | No (59%) | No (53%) | Yes (50%) | Yes (58%) | No (63%) | No (53%) | No (56%) | No (75%) | No (61%) | Yes (47%) |
| UGT2B7 | No | No (96%) | No (96%) | No (96%) | No (96%) | No (96%) | No (96%) | No (96%) | No (96%) | No (96%) | No (96%) |
| UGT2B15 | No (98%) | No (92%) | No (84%) | No (88%) | No (98%) | No | No | No (98%) | No | No (84%) | No (98%) |

**Table S6**. Possibility of being substrate and inhibitor of compounds for P-gp efflux and OATP1B1

| Compound | P-gp substrate | Pgp_Inh | OATP1B1_Inh |
| --- | --- | --- | --- |
| 2b | No (93%) | Yes (78%) | No (91%) |
| 3b | No (93%) | Yes (97%) | No (91%) |
| 5b | No (93%) | Yes (70%) | No (91%) |
| 1b | No (93%) | Yes (84%) | No (98%) |
| 4b | No (93%) | Yes (53%) | No (91%) |
| 6b | No (93%) | Yes (97%) | No (64%) |
| 7b | No (93%) | Yes (78%) | No (91%) |
| 8b | No (93%) | Yes (55%) | No (91%) |
| 9b | No (93%) | Yes (84%) | No (91%) |
| 10b | No (93%) | Yes (67%) | No (98%) |
| 11b | No (93%) | Yes (70%) | No (87%) |

1. P-gp substrate: likelihood of P-glycoprotein efflux substrate
2. Pgp_Inh: likelihood of P-glycoprotein inhibition
3. OATP1B1_Inh: inhibition of the hepatic OATP1B1 transporter

**Table S7**. The ADMET Predictor pharmacokinetics parameters analyses of compounds

|  | Compounds | | | | | | | | | | | |
| --- | --- | --- | --- | --- | --- | --- | --- | --- | --- | --- | --- | --- |
| Pharmacokinetics Parameters |  | **2b** | **3b** | **5b** | **1b** | **4b** | **6b** | **7b** | **8b** | **9b** | **10b** | **11b** |
|  | hum_fup% ^1^ | 3.666 | 4.27 | 3.758 | 4.293 | 2.96 | 2.65 | 3.945 | 3.0106 | 4.262 | 3.833 | 3.319 |
|  | rat_fup% ^2^ | 6.038 | 6.479 | 5.809 | 6.439 | 5.241 | 5.279 | 5.683 | 5.038 | 6.052 | 5.538 | 5.91 |
|  | Vd ^3^ | 0.533 | 0.533 | 0.7 | 0.556 | 0.561 | 0.789 | 0.668 | 0.699 | 0.627 | 0.53 | 0.477 |
|  | fu_mic_ ^4^ | 0.198 | 0.216 | 0.184 | 0.209 | 0.119 | 0.011 | 0.134 | 0.104 | 0.192 | 0.286 | 0.245 |
|  | RBP ^5^ | 0.575 | 0.549 | 0.586 | 0.56 | 0.572 | 0.578 | 0.579 | 0.589 | 0.564 | 0.56 | 0.57 |
|  | RBP_rat ^6^ | 0.979 | 0.972 | 1.068 | 0.981 | 1.018 | 1.114 | 1.096 | 1.08 | 1.045 | 0.974 | 1.029 |
|  | ECCS_Class ^7^ | Class_2 | Class_2 | Class_2 | Class_2 | Class_2 | Class_2 | Class_2 | Class_4 | Class_4 | Class_2 | Class_2 |
|  | S+CL_Renal ^8^ | No (73%) | No (73%) | Yes (74%) | No (62%) | No (56%) | Yes (55%) | Yes (69%) | Yes (74%) | Yes (74%) | No (58%) | No (77%) |
|  | S+CL_Uptake ^9^ | No (99%) | No (99%) | No (99%) | No (99%) | No (99%) | No (99%) | No (99%) | No (99%) | No (99%) | No (99%) | No (99%) |
|  | S+CL_Metab ^10^ | No (65%) | No (94%) | No (94%) | No (94%) | No (74%) | No (94%) | No (94%) | No (94%) | No (94%) | No (94%) | No (94%) |

1. hum_fup%: Human plasma protein binding as percent unbound
2. rat_fup%: Rat plasma protein binding as percent unbound
3. Vd: Human volume of distribution
4. S+fu_mic_: Fraction unbound in human liver microsomes
5. RBP: Human blood-to-plasma concentration ratio
6. RBP_rat: Rat blood-to-plasma concentration ratio
7. ECCS_Class: Extended clearance classification system
8. S+CL_Renal: predicts whether or not renal elimination will be critical to clearance.
9. S+CL_Uptake: predicts whether or not hepatic uptake will be critical to clearance.
10. S+CL_Metab: predicts whether or not metabolism will be critical to clearance

**Table S8.** The docking binding energy of DHPS of *S. aureus* and *E. coli* and eleven SMs using Autodock Vina

| Complex | Binding Energy (kcal/mol) | |
| --- | --- | --- |
|  | **S. aureus** | **E. coli** |
| 1b | -15.20 | -17.70 |
| 2b | -14.10 | -17.10 |
| 3b | -16.40 | -17.20 |
| 4b | -19.20 | -17.80 |
| 5b | -17.40 | -17.50 |
| 6b | -14.80 | -18.60 |
| 7b | -16.70 | -16.70 |
| 8b | -15.50 | -15.40 |
| 9b | -16.50 | -17 |
| 10b | -16.60 | -17.60 |
| 11b | -13.40 | -15 |

| ***Staphylococcus aureus*** | |
| --- | --- |
| 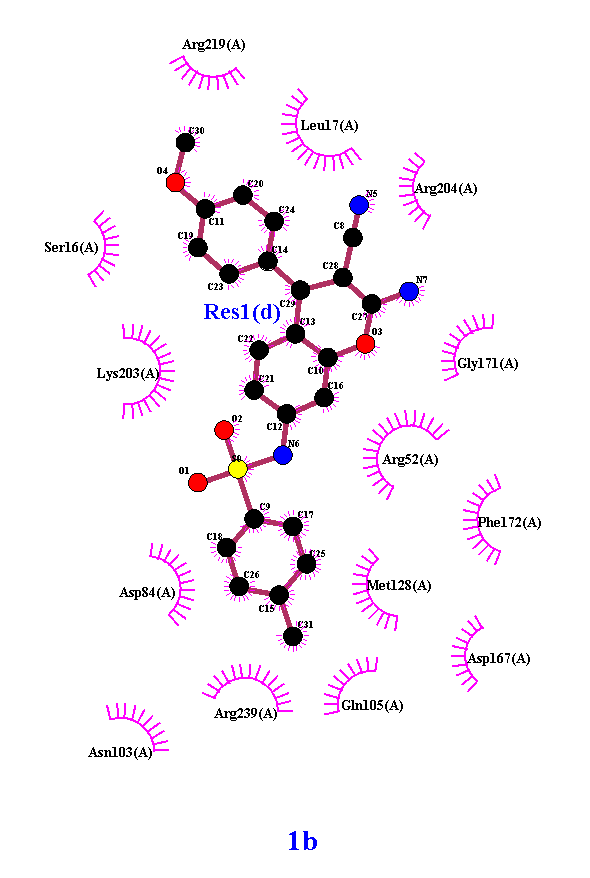 | 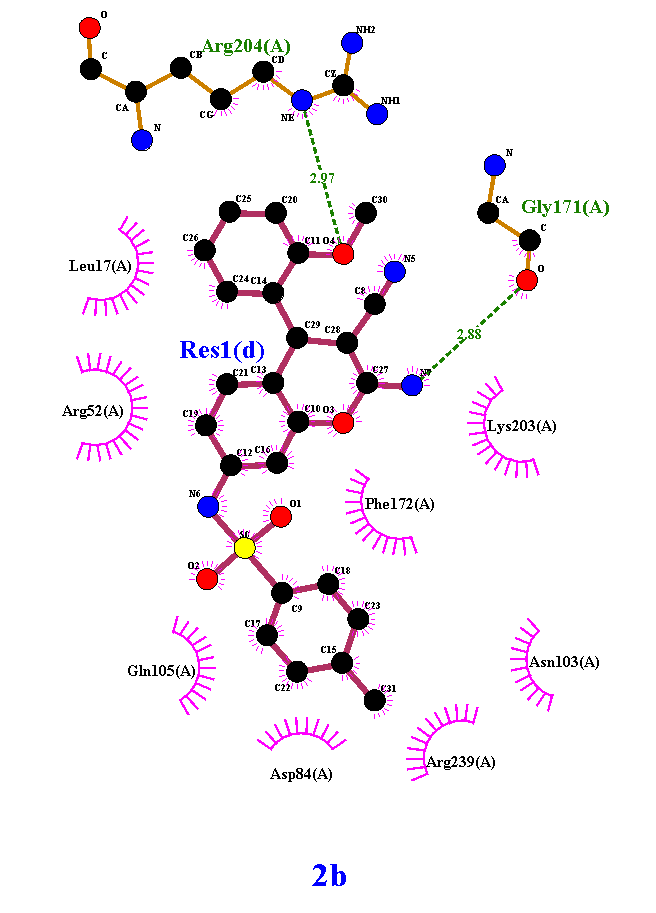 |
| 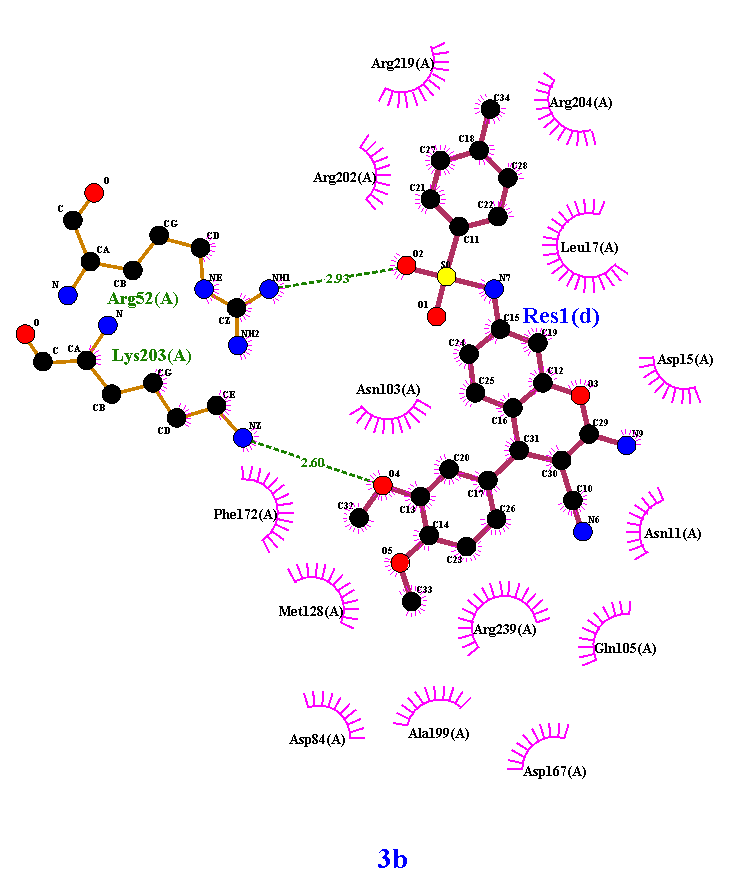 | 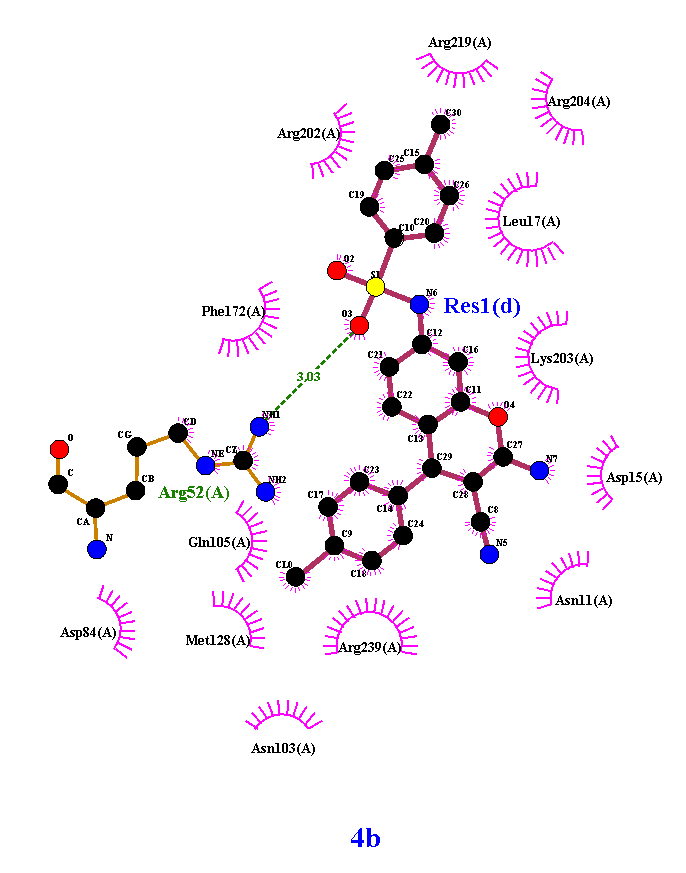 |
| 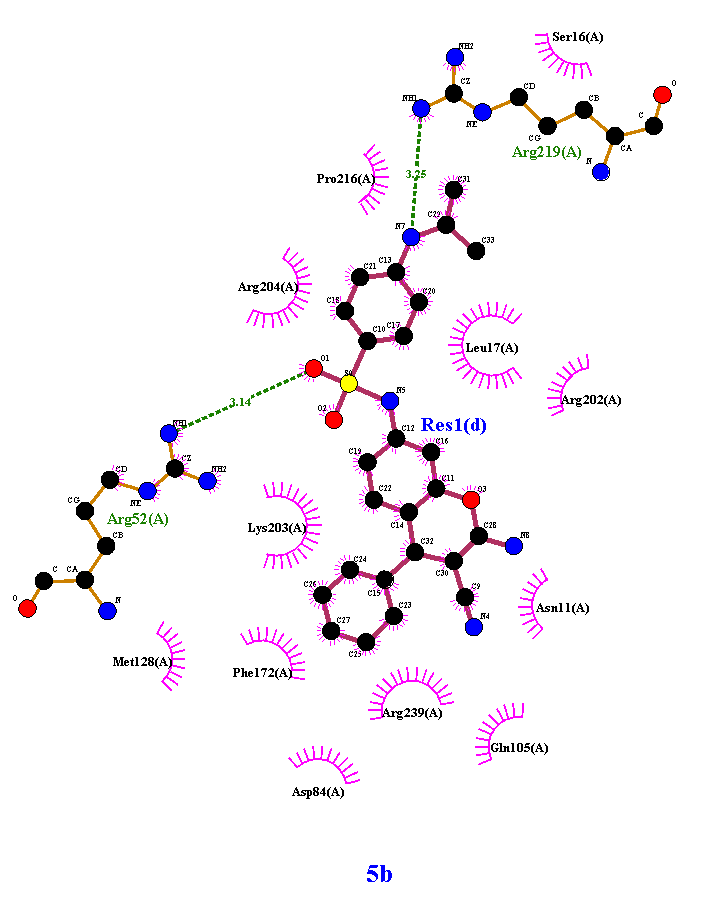 | 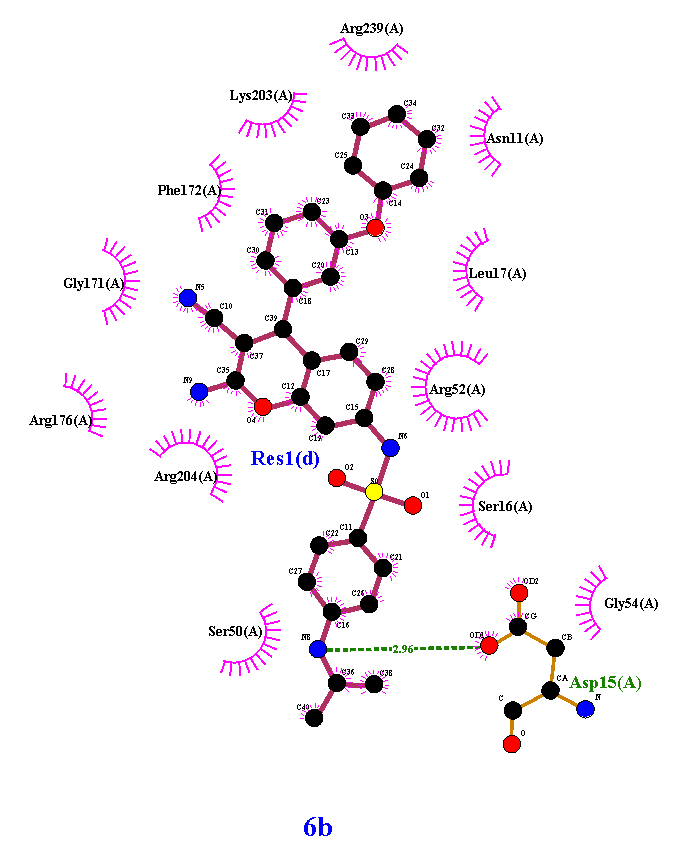 |
| 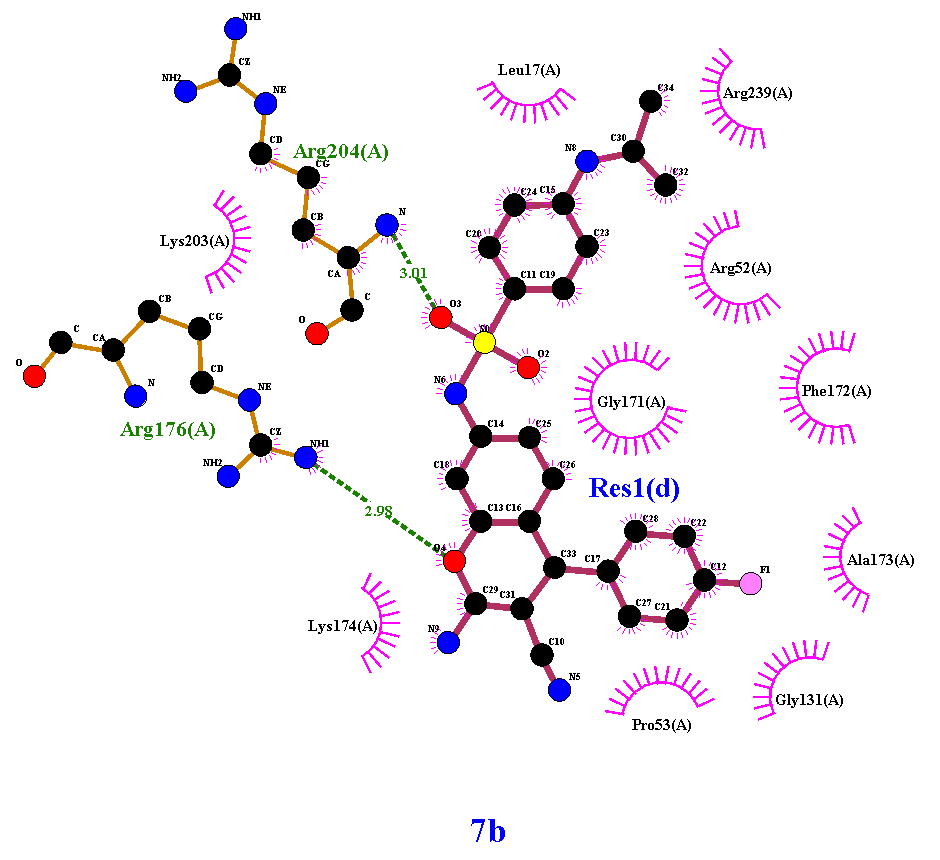 | 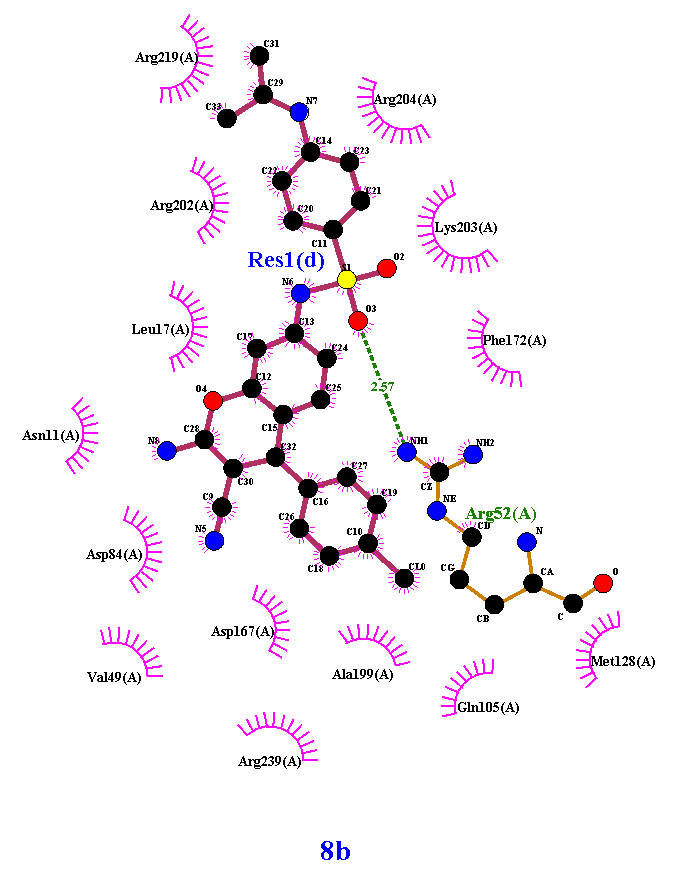 |
| 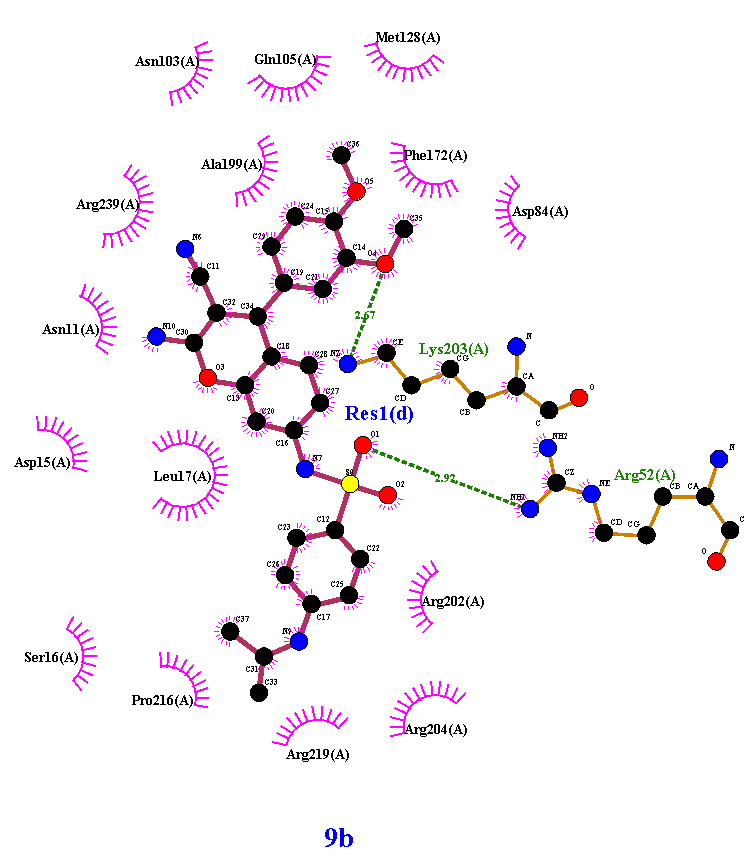 | 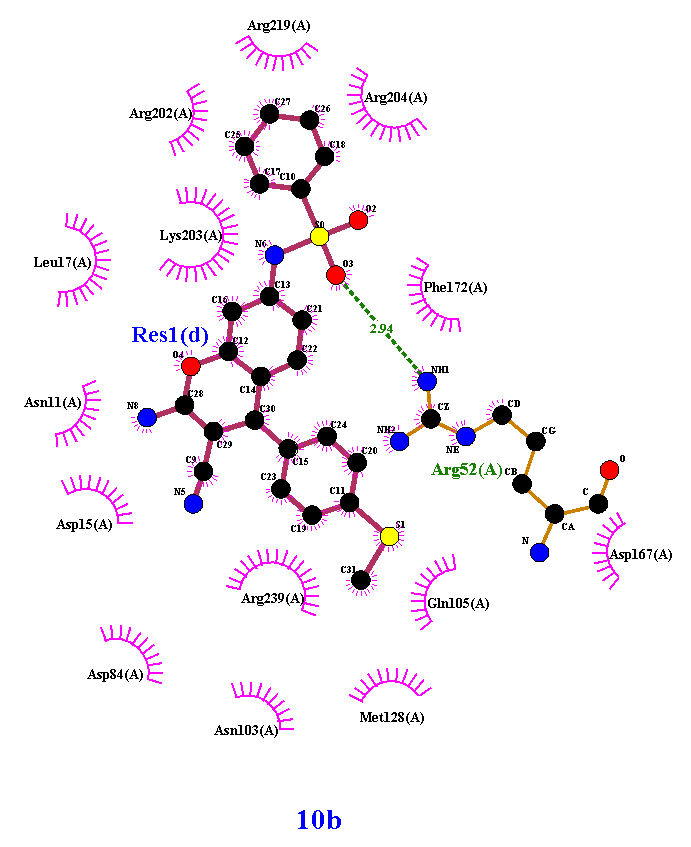 |
| 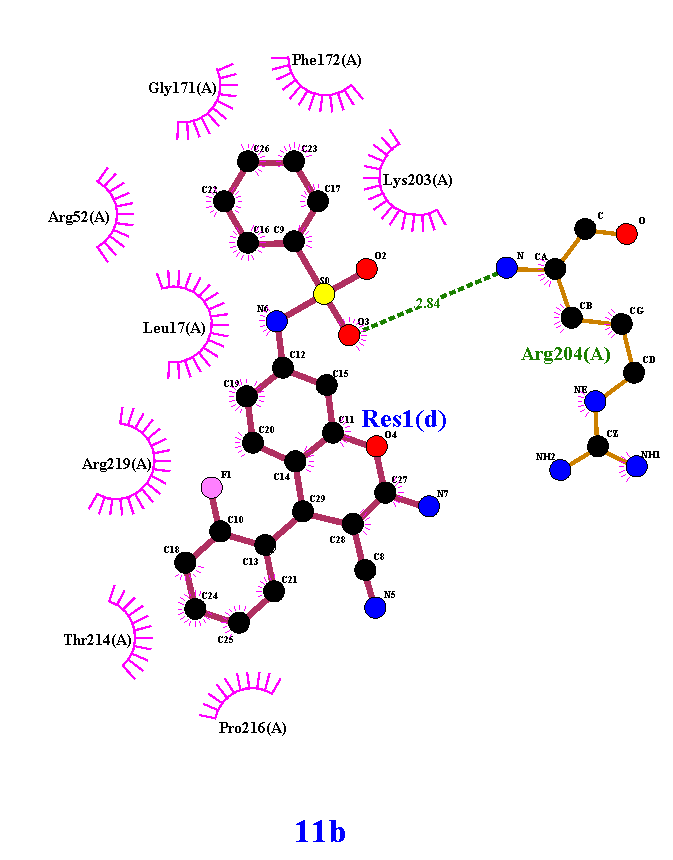 | |

**Figure S1.** *S. aureus*, binding interactions with eleven different compounds were analyzed using Ligplot. This analysis highlighted the hydrogen bonds as green lines and the hydrophobic interactions as red dotted lines, providing a comprehensive visualization of the binding dynamics within the active site.

| ***Escherichia coli*** | |
| --- | --- |
| 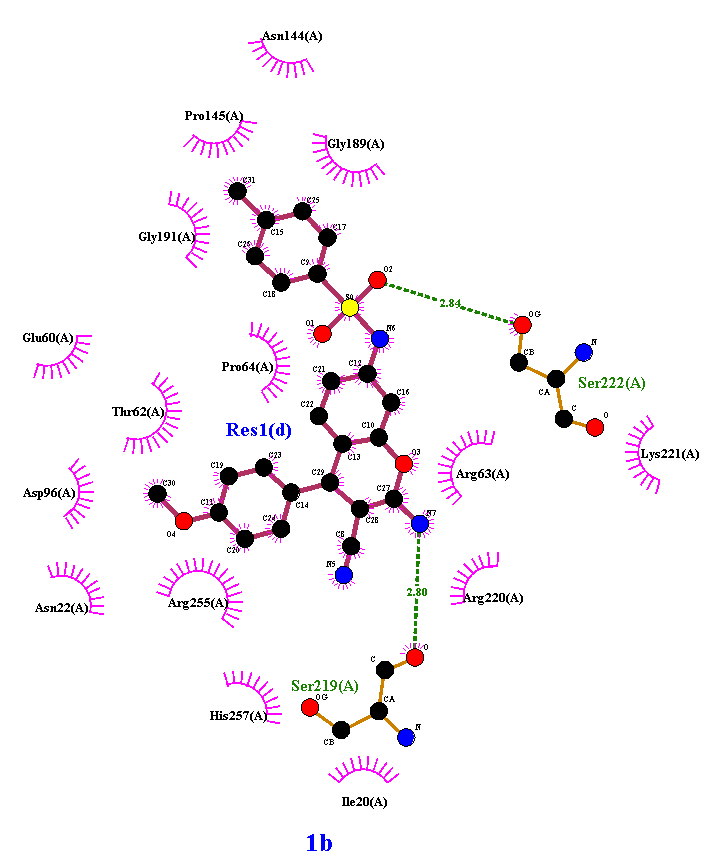 | 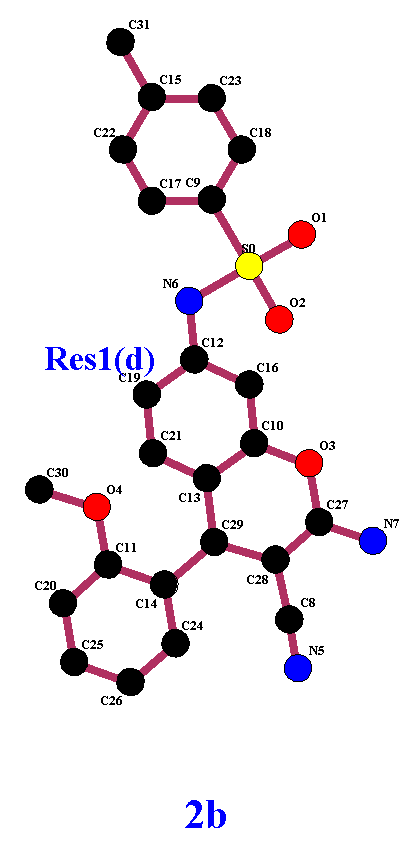 |
| 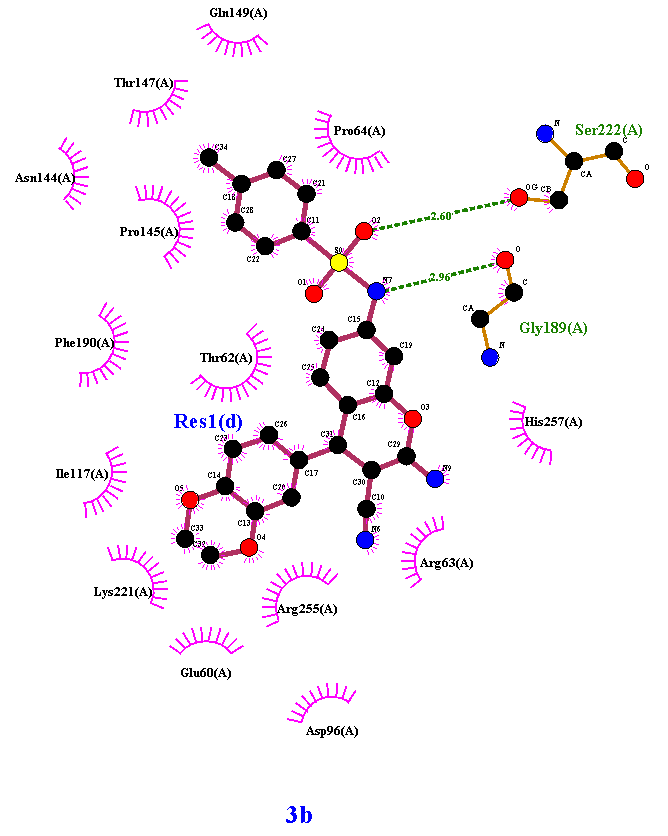 | 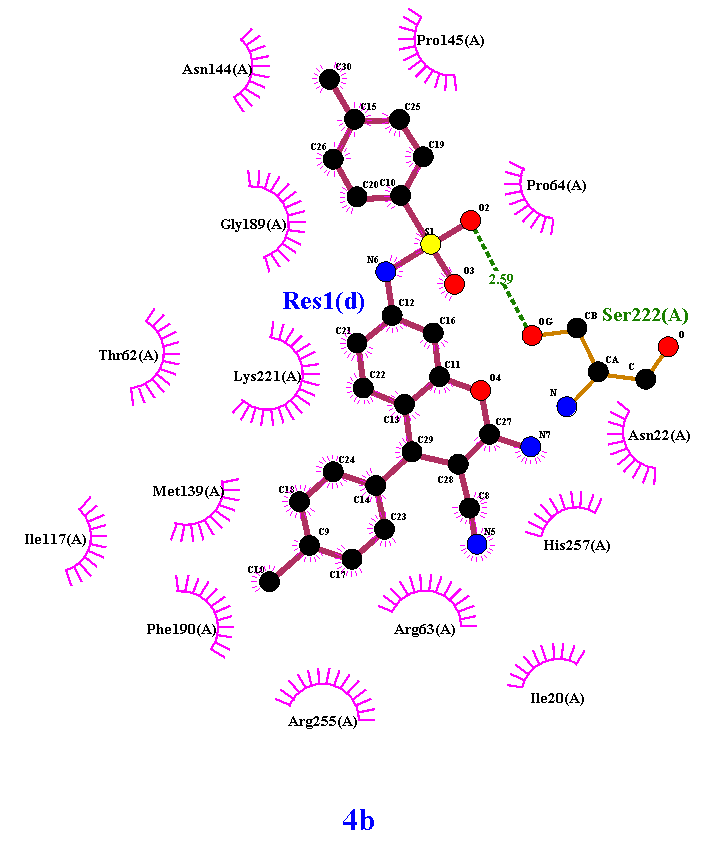 |
| 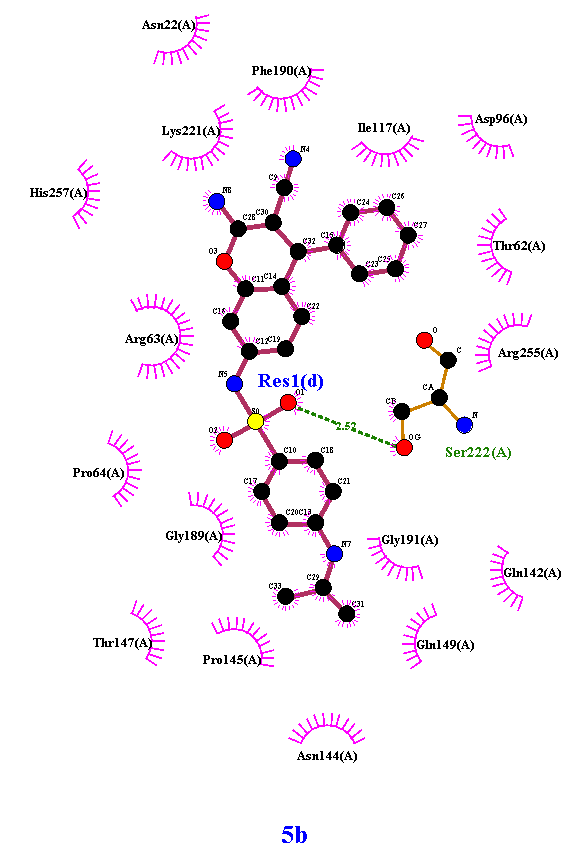 | 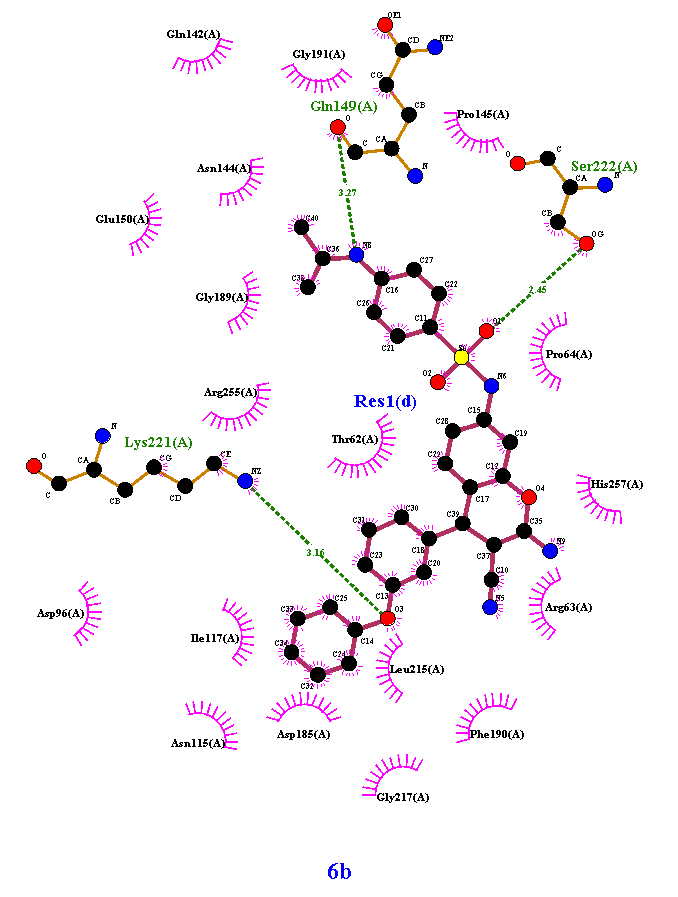 |
| 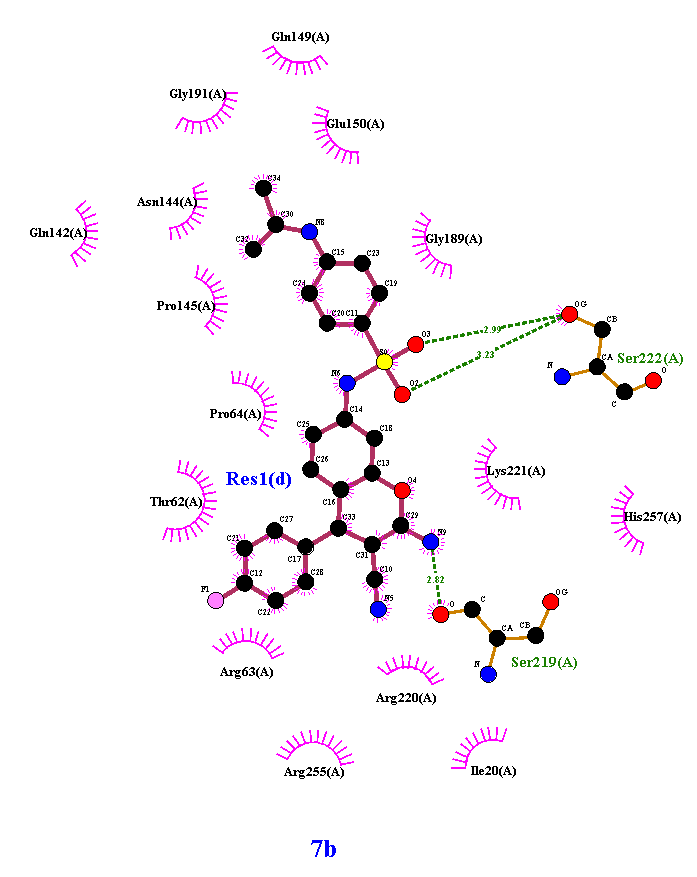 | 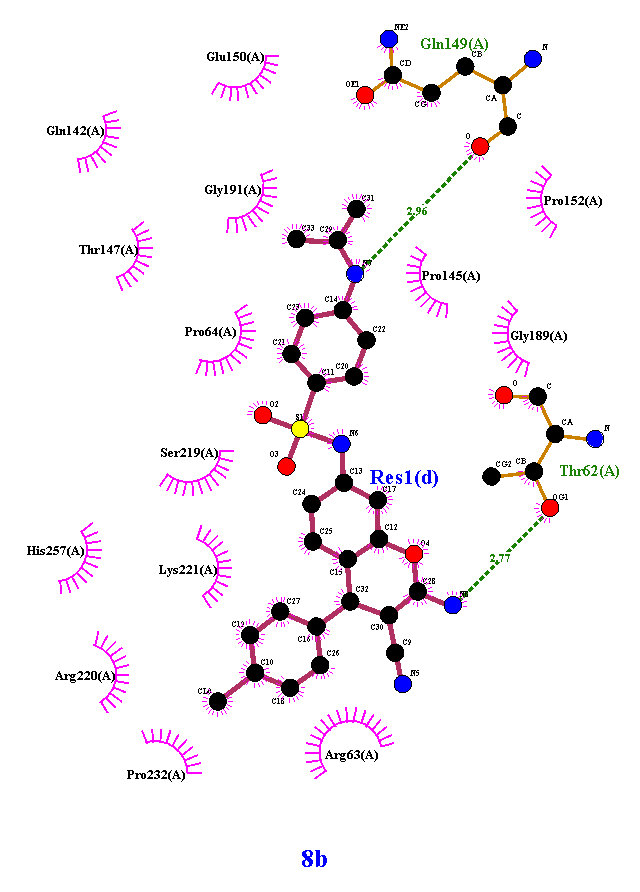 |
| 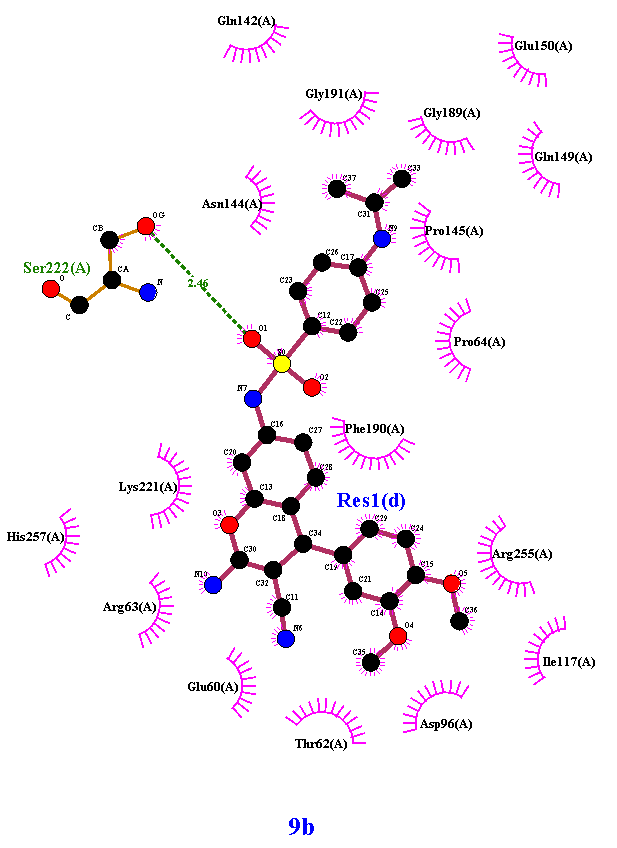 | 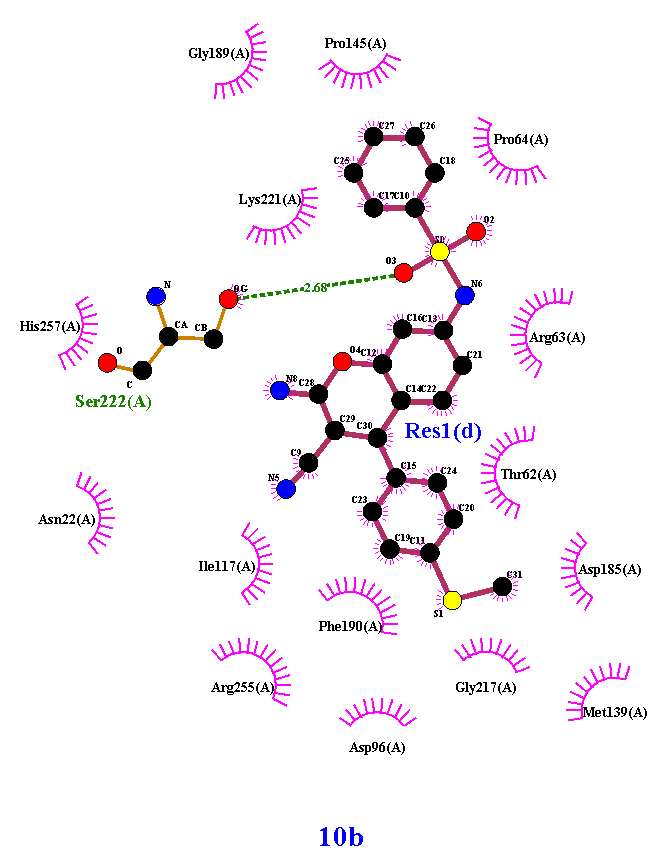 |
| 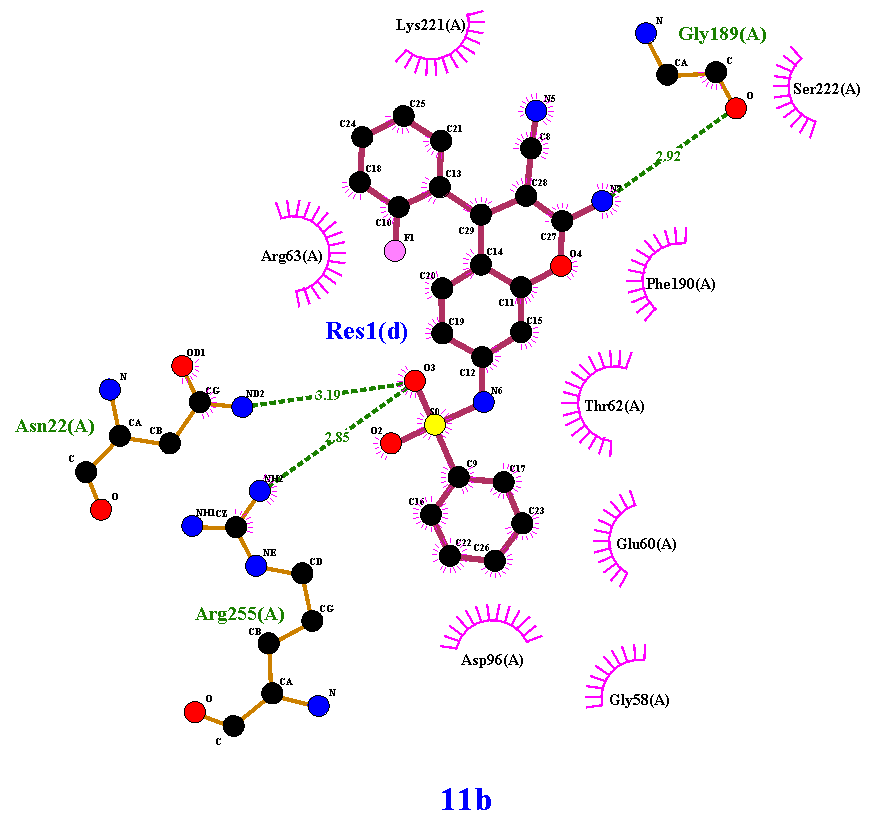 | |

**Figure S2.** *E. coli* DHPS complexes with studied compounds. Hydrogen bonds were depicted using green lines, and hydrophobic interactions were illustrated with red dotted lines, offering a detailed view of the molecular interactions involved.

**Table S9. The hydrogen bonds and hydrophobic interactions between the DHPS od S. aureus and of E. coli with compounds, along with the corresponding distance**

| Complex | Microorganism | Hydrogen Bonds | | Hydrophobic Interaction | |
| --- | --- | --- | --- | --- | --- |
|  |  | Residue | Distance (Å) | Amino acids | Distance (Å) |
| 1b | S. aureus | - | - | Ser16, Leu17, Asp84, Asn103, Lys203, Arg219, Arg204, Gly171, Arg52, Phe172, Met128, Asp167, Gln105, Arg239 | 3.30-4.02 |
|  | E. coli | Ser222 | 2.84 | Asn144, Pro145, Gly189, Gly191, Pro64, Glu60, Thr62, Asp96, Asn22, Arg255, His 257, Ile20, Gly189, Lys221, Arg63, Arg220 | 3.20-4.31 |
| 2b | S. aureus | Gly171, Arg204 | 2.88-2.97 | Leu17, Arg52, Gln105, Asp84, Lys203, Phe172, Asn103, Arg239 | 3.40-4.30 |
|  | E. coli | - | - | - | - |
| 3b | S. aureus | Arg52, Lys203, | 2.60-2.93 | Arg219, Arg202, Arg204, Leu17, Asp15, Asn11, Gln105, Arg239, Asp167, Ala199, Asp84, Met128, Phe172, Asn103 | 3.46-4.40 |
|  | E. coli | Ser222, Gly189 | 2.6-2.96 | Gln149, Pro64, Thr147, Asn144, Pro145, Phe190, Thr62, Ile117, Lys221, Glu60, Arg255, Arg63, His257, Asp96 | 3.23-3.94 |
| 4b | S. aureus | Arg52 | 3.03 | Phe172, Arg202, Arg219, Arg204, Leu17, Lys203, Asp15, Asn11, Arg239, Met128, Asn103, Asp84, Gln105 | 3.08-4.69 |
|  | E. coli | Ser222 | 2.59 | Pro145, Asn144, Pro64, Asn22, His257, Arg63, Ile20, Arg255, Phe190, Met139, Ile117, Thr62, Lys221, Gly189 | 3.02-4.5 |
| 5b | S. aureus | Arg52, Arg219 | 3.14-3.25 | Arg204, Pro216, Ser16, Leu17, Arg202, Asn11, Arg239, Gln105, Asp84, Phe172, Met128, Lys203 | 3.36-4.4 |
|  | E. coli | Ser222 | 2.52 | Asn22, Lys221, Phe190, Ile117, Asp96, Thr62, Arg255, Gly191, Gln142, Gln149, Asn144, Pro145, Thr147, Gly189, Pro64, Arg63, His257 | 3.01-4.54 |
| 6b | S. aureus | Asp15 | 2.96 | Arg204, Arg176, Gly171, Phe172, Lys203, Arg239, Asn11, Leu17, Arg52, Ser16, Gly54, Ser50 | 3.0-4.30 |
|  | E. coli | Gln149, Lys221, Ser222 | 2.45-3.27 | Gln142, Gly191, Asn144, Glu150, Gly189, Arg255, Thr62, Asp96, Ile117, Asn115, Asp185, Leu215, Gly217, Phe190, Arg63, His257, Pro64, Pro145 | 3.04-4.10 |
| 7b | S. aureus | Arg176, Arg204 | 2.08-3.01 | Leu17, Arg239, Arg52, Gly171, Phe172, Ala173, Gly131, Pro53, Lys174, Lys203 | 3.1-4.02 |
|  | E. coli | Ser219, Ser222 | 2.82-3.23 | Gly191, Gln149, Glu150, Gly189, Lys221, His257, Ile20, Arg220, Arg255, Arg63, Thr62, Pro64, Pro145, Asn144, Gln142 | 3.32-4.23 |
| 8b | S. aureus | Arg52 | 2.57 | Arg219, Arg202, Leu17, Asn11, Asp84, Val49, Asp167, Arg239, Ala199, Gln105, Met128, Phe172, Lys203, Arg204 | 3.0-4.69 |
|  | E. coli | Thr62, Gln149 | 2.77-2.96 | Glu150, Gln142, Gly191, Thr147, Pro64, Ser219, His 257, Lys221, Arg220, Pro232, Arg63, Gly189, Pro145, Pro152 | 3.51-4.10 |
| 9b | S. aureus | Arg52, Lys203 | 2.67-2.92 | Asn103, Gln105, Met128, Peh172, Asp84, Arg202, Arg204, Arg219, Pro216, Ser16, Leu17, Asp15, Asn11, Arg239, Leu17, Ala199 | 3.1-3.9 |
|  | E. coli | Ser222 | 2.46 | Gln142, Gly191, Gly189, Glu150, Gln149, Pro145, Pro64, Phe190, Arg255, Ile117, Asp96, Thr62, Glu60, Arg63, Lys221, His257, Asn144 | 3.20-3.80 |
| 10b | S. aureus | Arg52 | 2.94 | Arg219, Arg202, Arg204, Phe172, Asp167, Gln105, Arg239, Met128, Asn103, Asp84, Asp15, Asn11, Leu17, Lys203, Arg239, | 3.40-4.30 |
|  | E. coli | Asn22, Gly189, Arg255 | 2.68 | Gly189, Pro145, Pro64, Arg63, Thr62, Asp185, Gly217, Met139, Asp96, Phe190, Arg255, Ile117, Asn22, His257, Lys221 | 3.52-3.98 |
| 11b | S. aureus | Arg204 | 2.84 | Gly171, Phe172, Lys203, Pro216, Thr214, Arg219, Leu17, Arg52 | 3.10-4.01 |
|  | E. coli | Ser222 | 2.85-3.19 | Lys221, Ser222, Phe190, Thr62, Glu60, Gly58, Asp96, Arg63 | 3.24-3.93 |

**Spectral data of the synthesized products:**

| 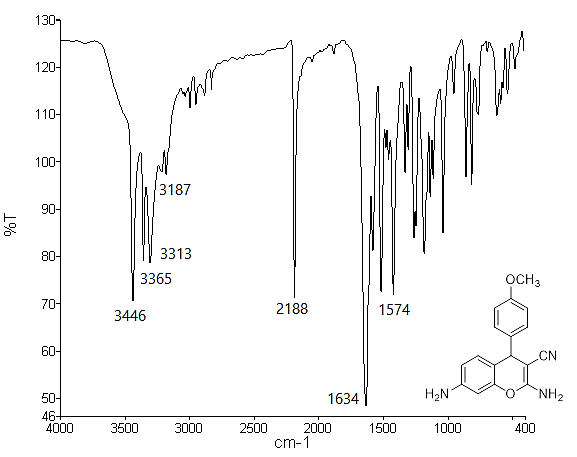 |
| --- |
| **Figure S3. IR spectra of 2,7-diamino-4-(4-methoxyphenyl)-4H-chromene-3-carbonitrile** |

| 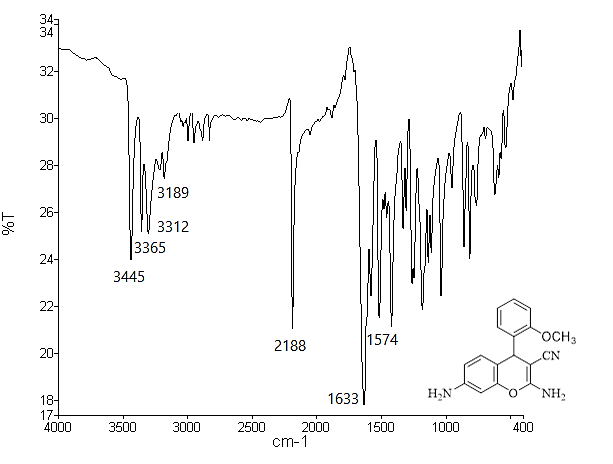 |
| --- |
| **Figure S4. IR spectra of 2,7-diamino-4-(2-methoxyphenyl)-4H-chromene-3-carbonitrile** |

| **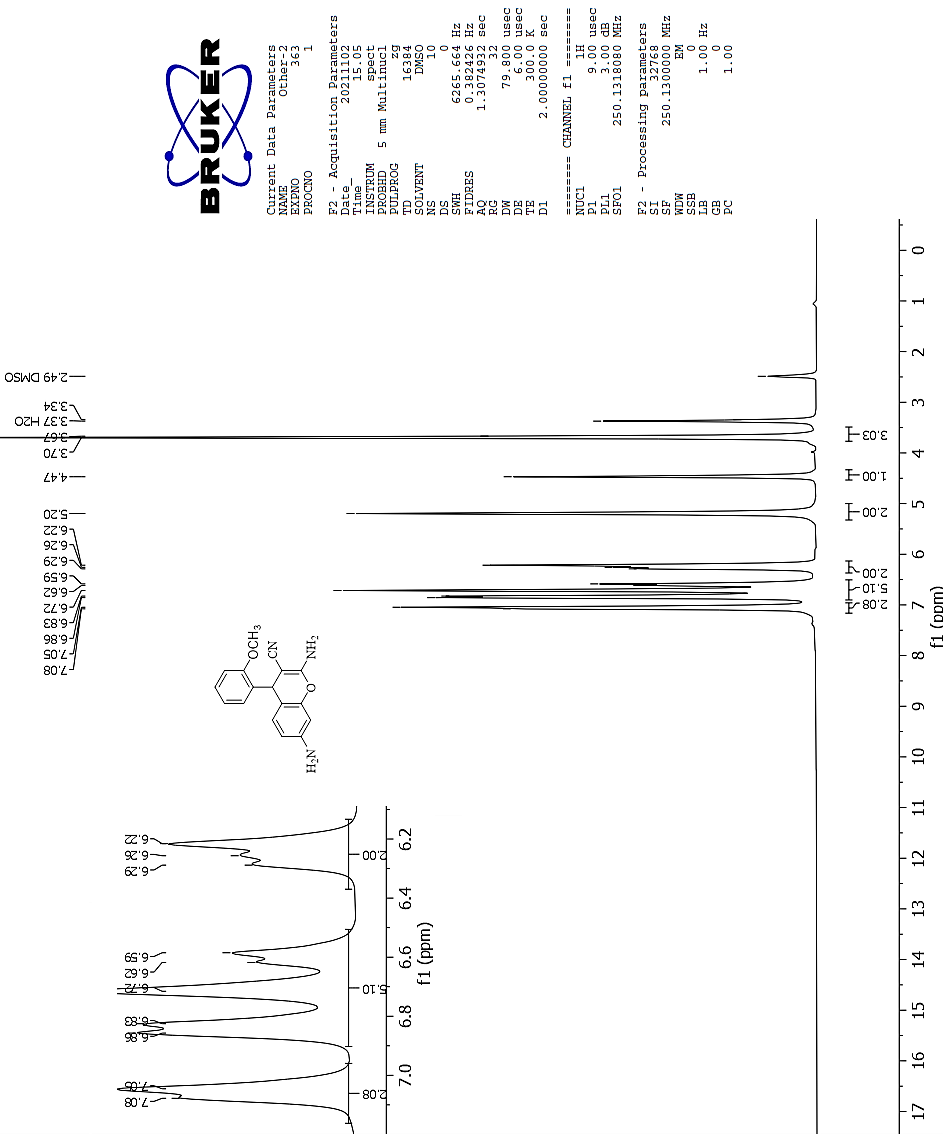** | **Figure S5. ^1^H NMR of spectra of 2,7-diamino-4-(2-methoxyphenyl)-4H-chromene-3-carbonitrile** |
| --- | --- |

| **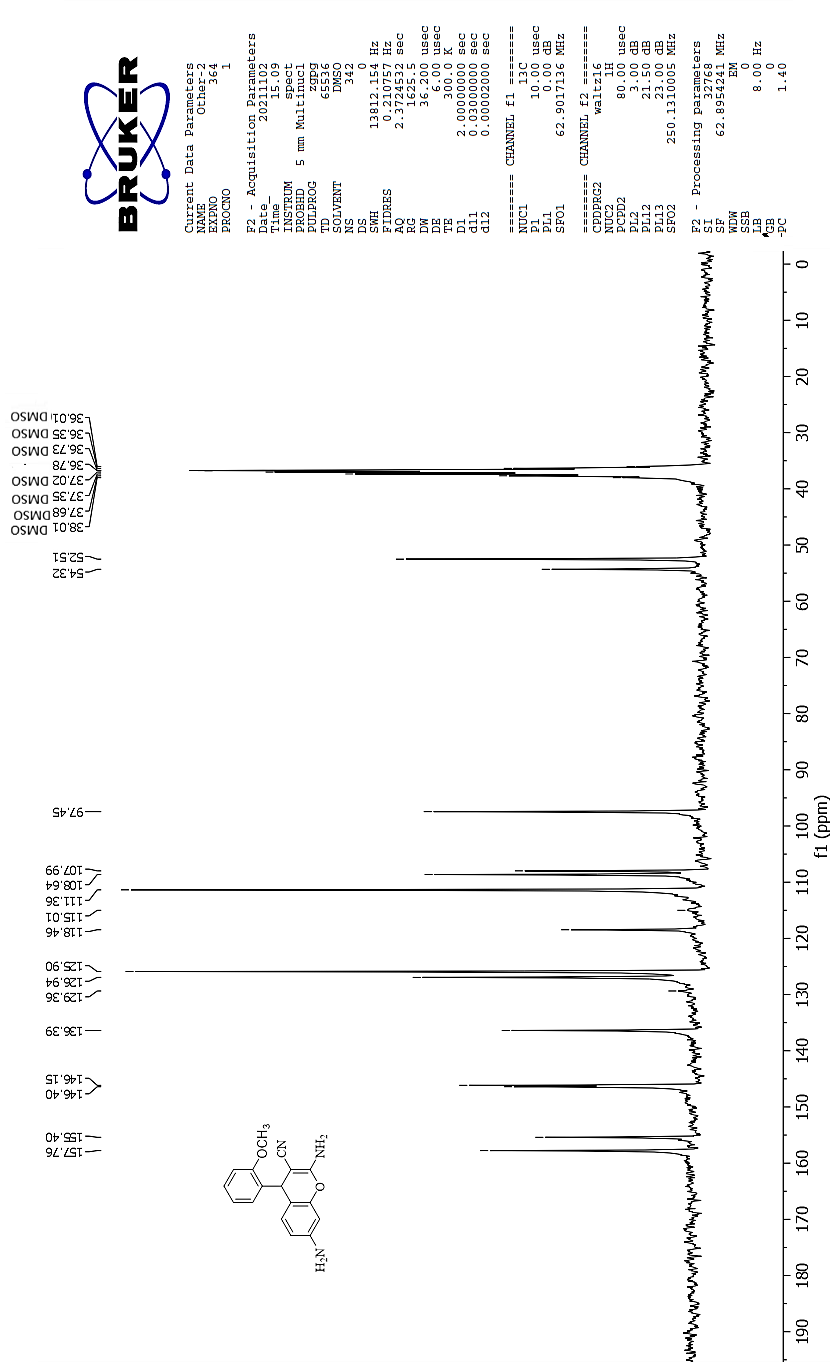** | **Figure S6. ^13^C NMR of spectra of 2,7-diamino-4-(2-methoxyphenyl)-4H-chromene-3-carbonitrile** |
| --- | --- |

| 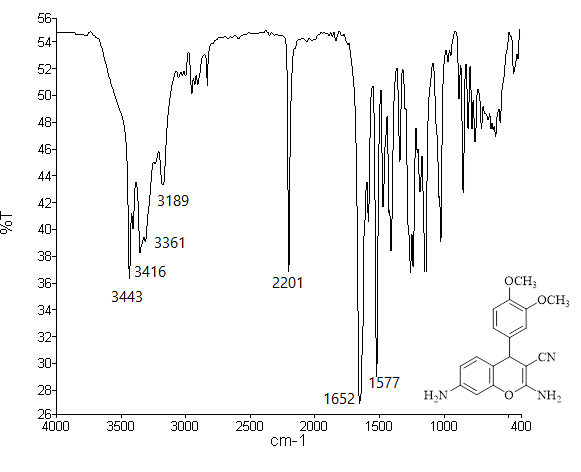 |
| --- |
| **Figure S7. IR spectra of 2,7-diamino-4-(3,4-dimethoxyphenyl)-4H-chromene-3-carbonitrile** |

| **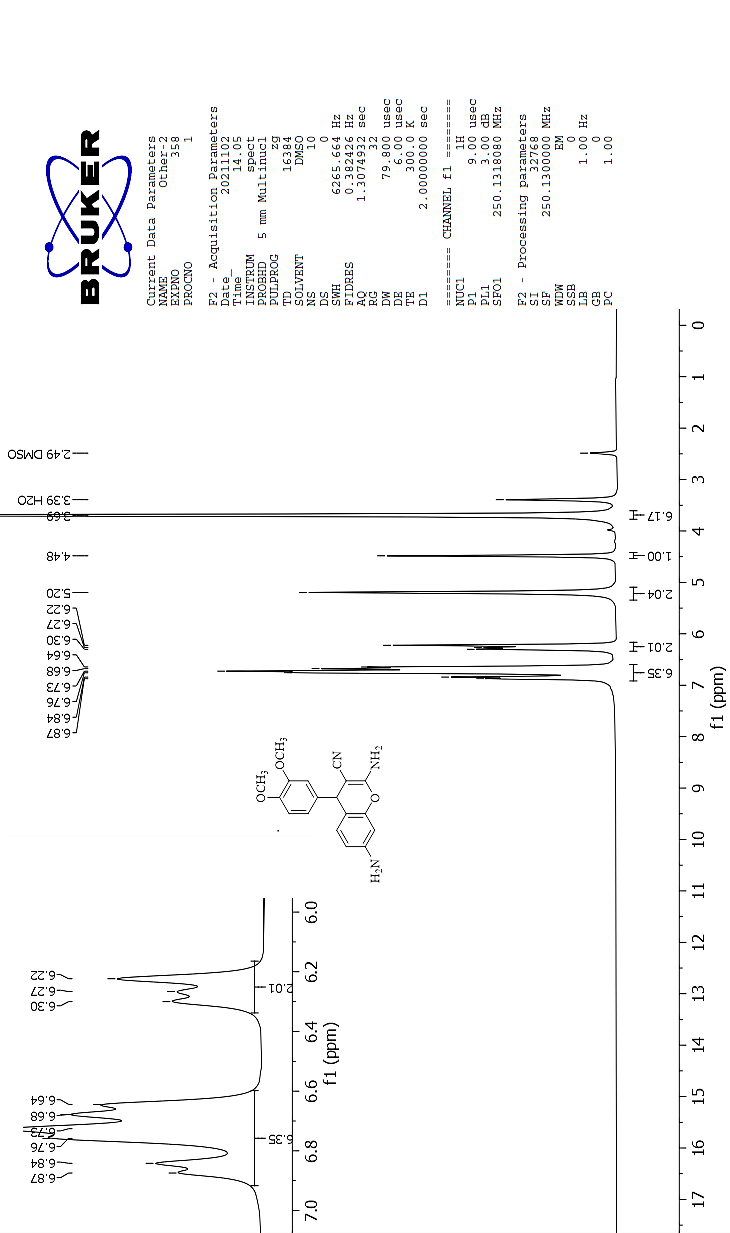** | | | **Figure S8. ^1^H NMR of spectra of 2,7-diamino-4-(3,4-dimethoxyphenyl)-4H-chromene-3-carbonitrile** |
| --- | --- | --- | --- |
| **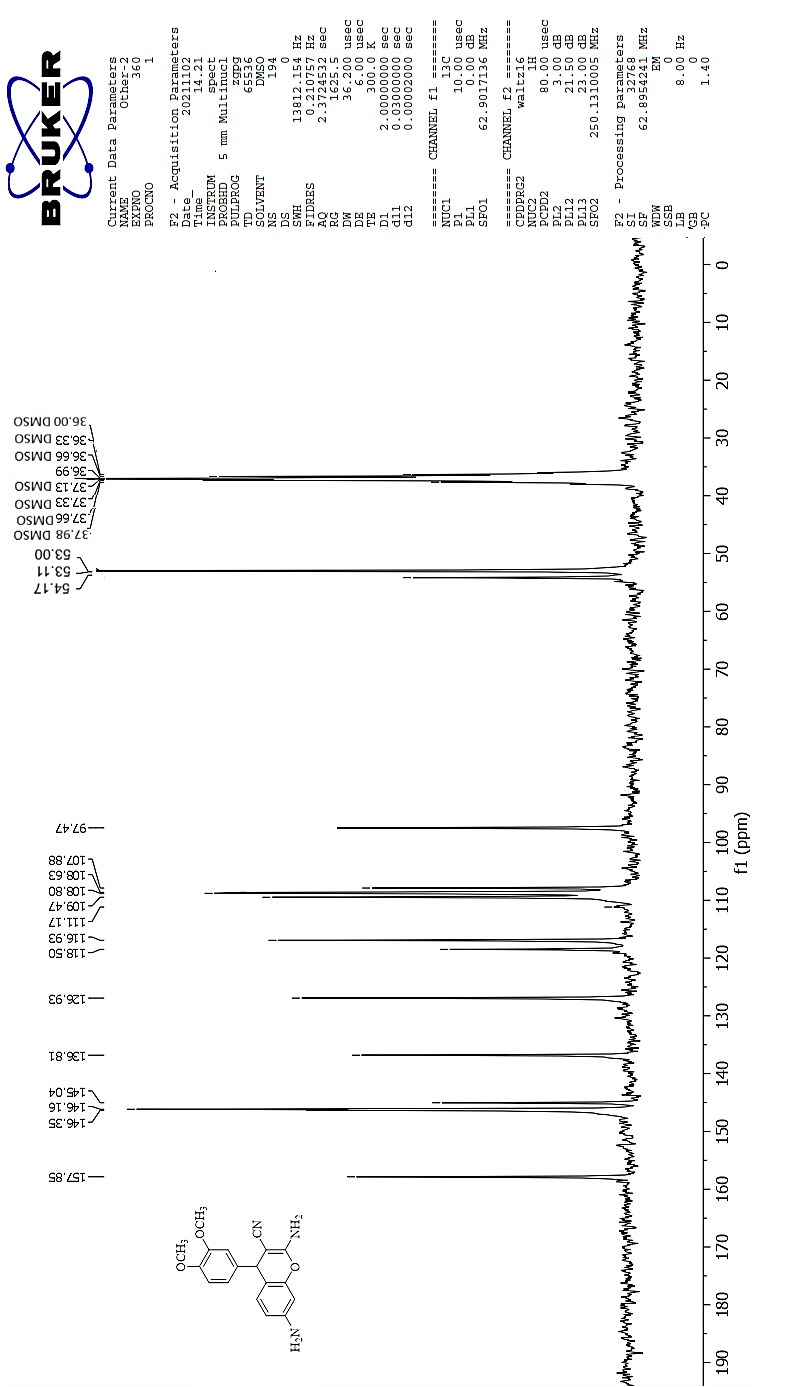** | **Figure S9. ^13^CNMR of spectra of 2,7-diamino-4-(3,4-dimethoxyphenyl)-4H-chromene-3-carbonitrile** |  |  |

| 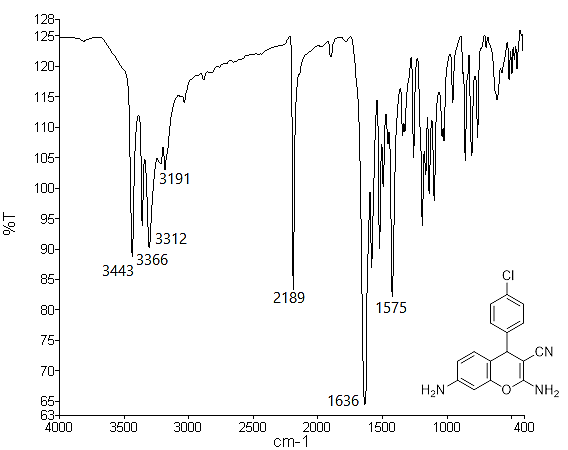 |
| --- |
| **Figure S10. IR spectra of 2,7-diamino-4-(4-chlorophenyl)-4H-chromene-3-carbonitrile** |

| 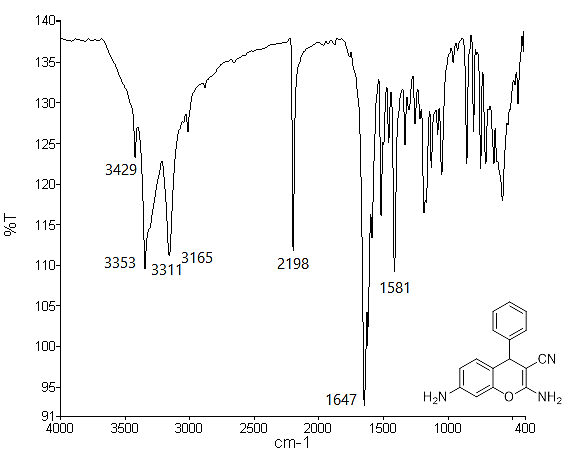 |
| --- |
| **Figure S11. IR spectra of 2,7-diamino-4-phenyl-4H-chromene-3-carbonitrile** |

| 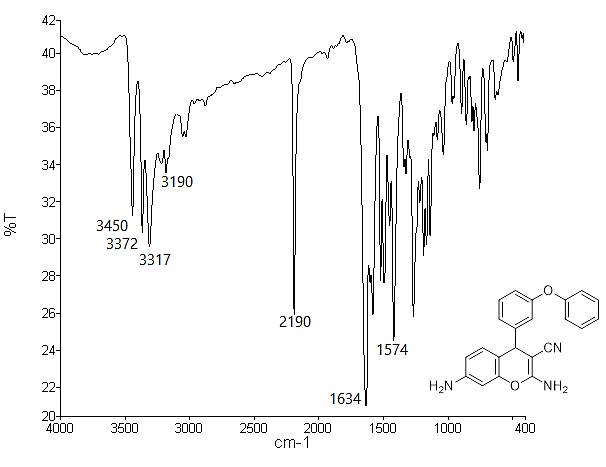 |
| --- |
| **Figure S12. IR spectra of 2,7-diamino-4-(3-phenoxyphenyl)-4H-chromene-3-carbonitrile** |

| 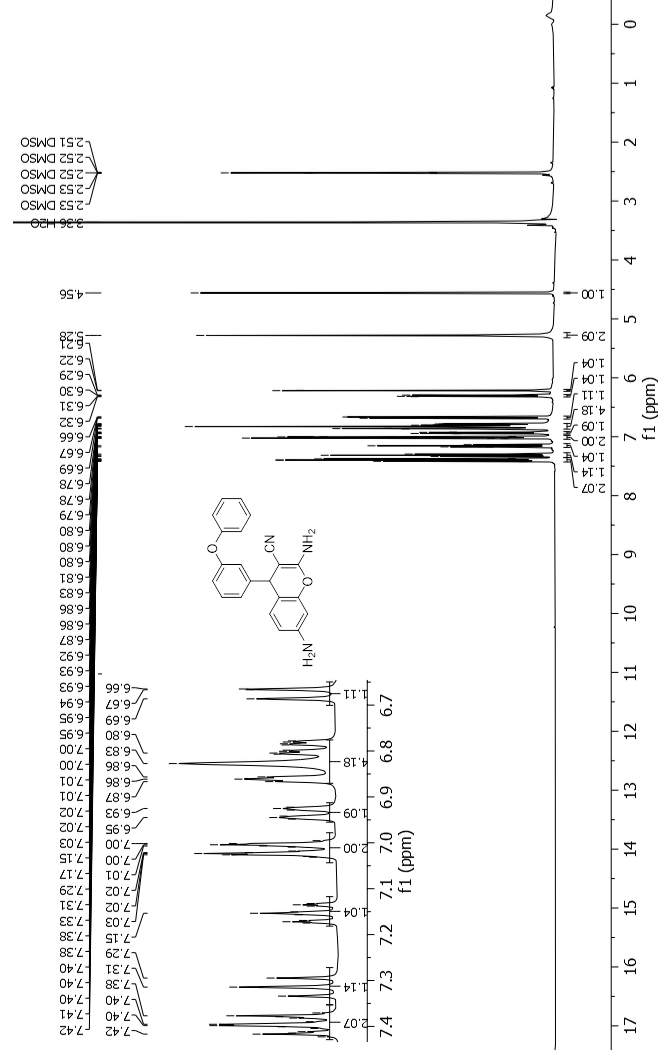 | **Figure S13. ^1^H NMR of spectra of 2,7-diamino-4-(3-phenoxyphenyl)-4H-chromene-3-carbonitrile** |  |  |
| --- | --- | --- | --- |
| 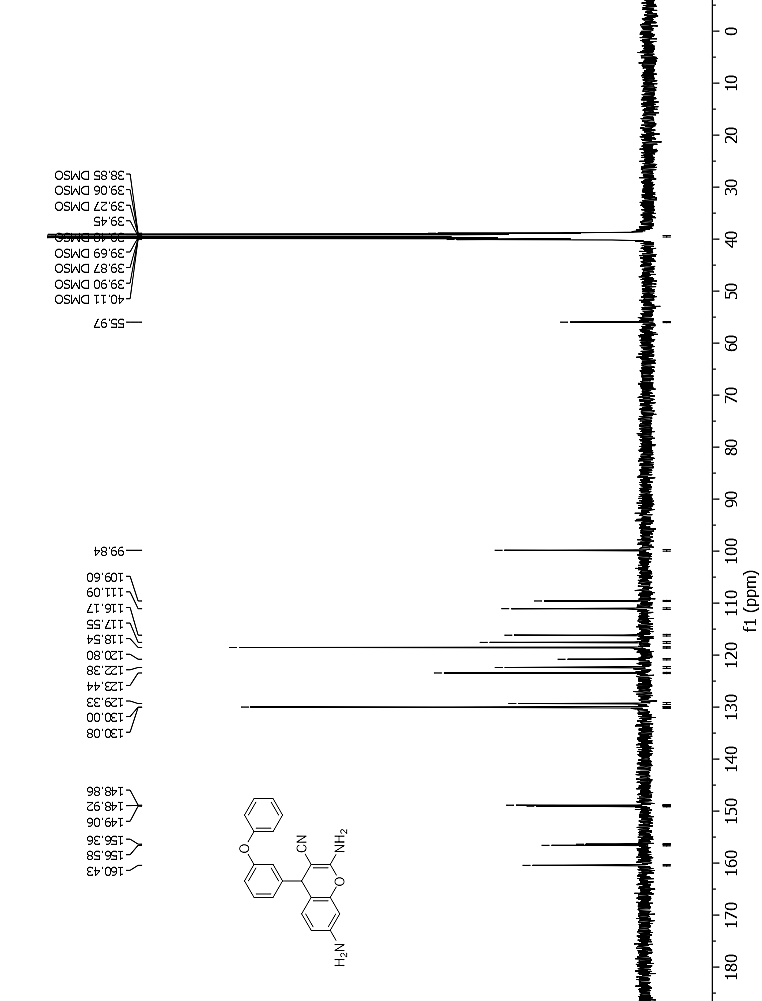 | | | **Figure S14 ^13^C NMR of spectra of 2,7-diamino-4-(3-phenoxyphenyl)-4H-chromene-3-carbonitrile** |

| 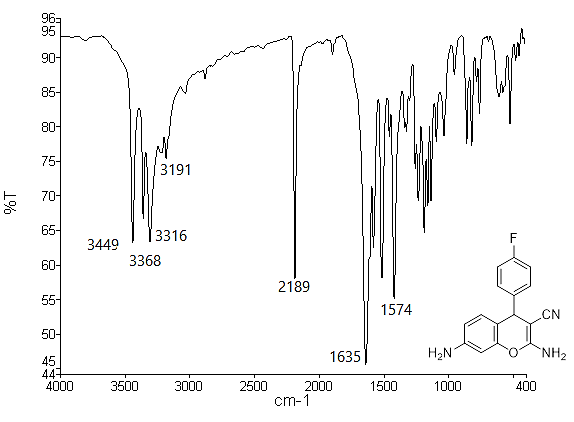 |
| --- |
| **Figure S15. IR spectra of 2,7-diamino-4-(4-fluorophenyl)-4H-chromene-3-carbonitrile** |

| 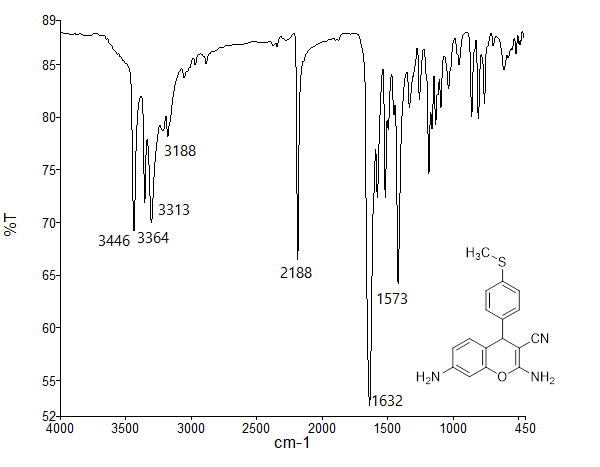 |
| --- |
| **Figure S16. IR spectra of 2,7-diamino-4-(4-(methylthio)phenyl)-4H-chromene-3-carbonitrile** |

| 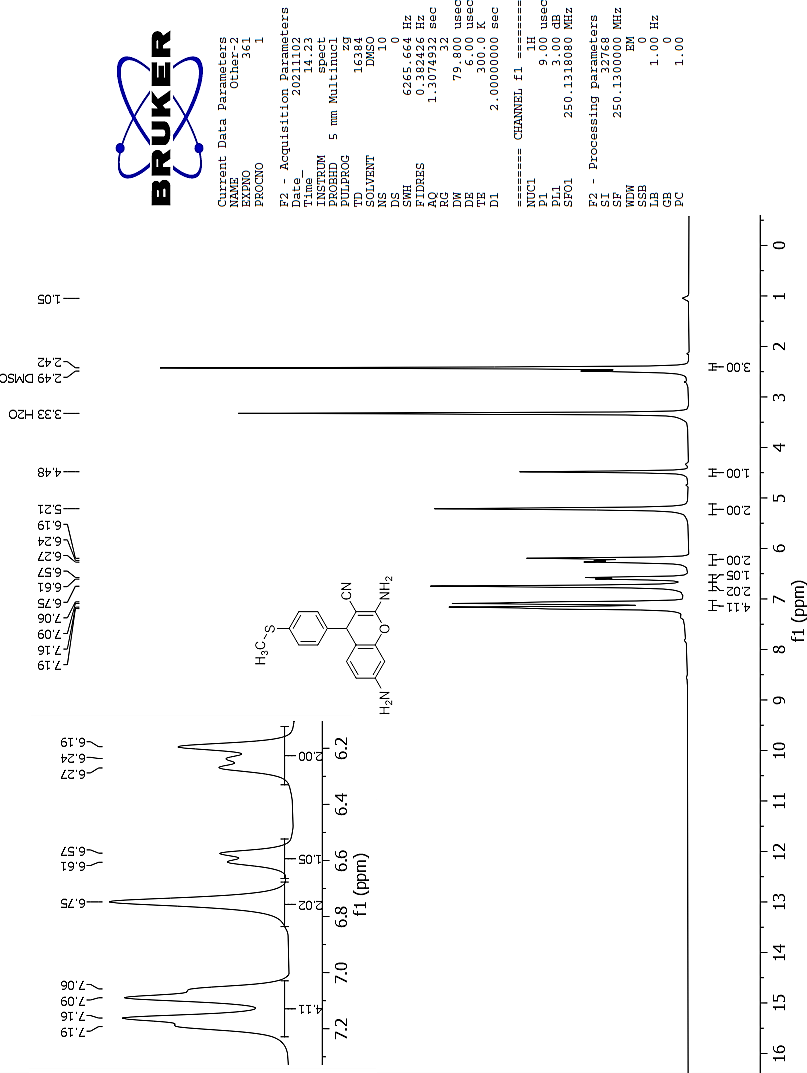 | **Figure S17. ^1^H NMR of spectra of 2,7-diamino-4-(4-(methylthio)phenyl)-4H-chromene-3-carbonitrile** |
| --- | --- |

| 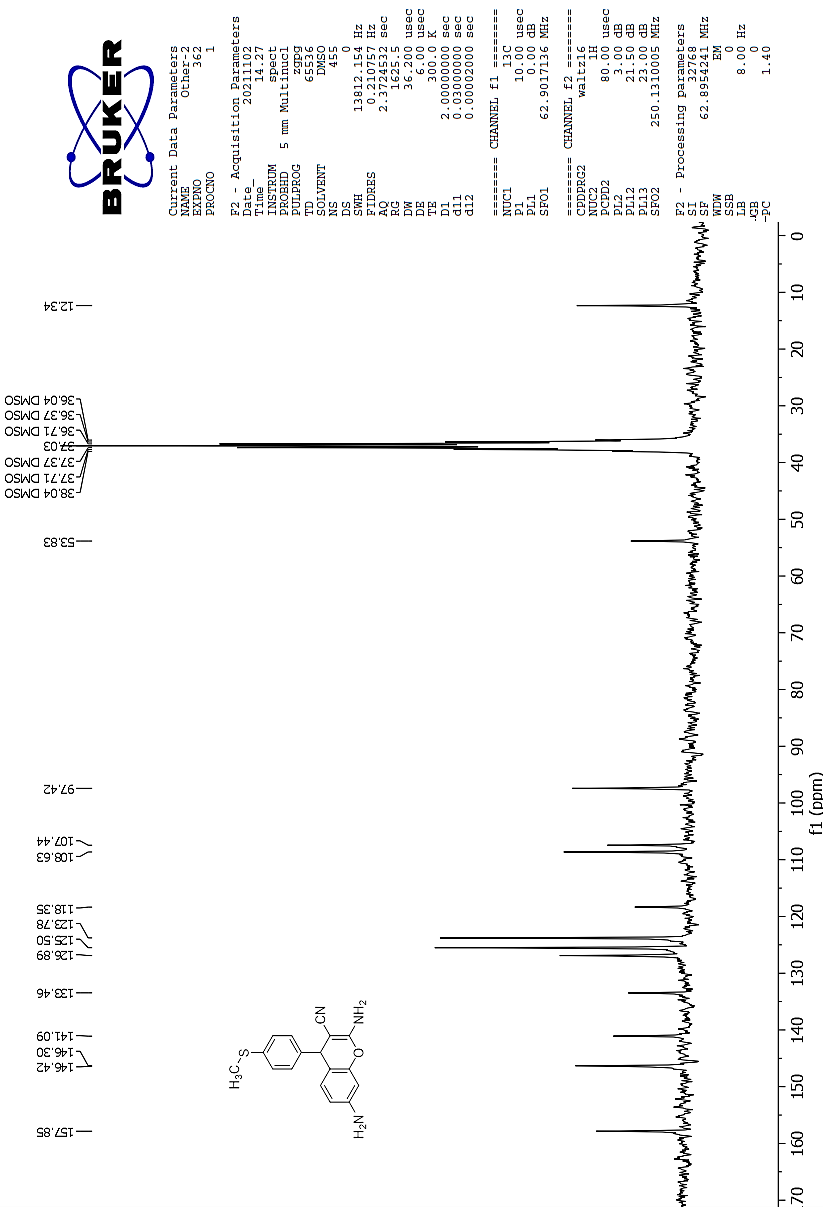 | **Figure S18. ^13^CNMR of spectra of 2,7-diamino-4-(4-(methylthio)phenyl)-4H-chromene-3-carbonitrile** |
| --- | --- |

| 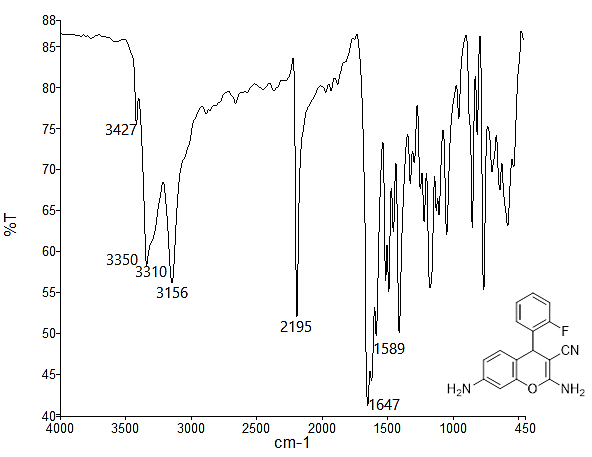 |
| --- |
| **Figure S19. IR spectra of 2,7-diamino-4-(2-fluorophenyl)-4H-chromene-3-carbonitrile** |

| 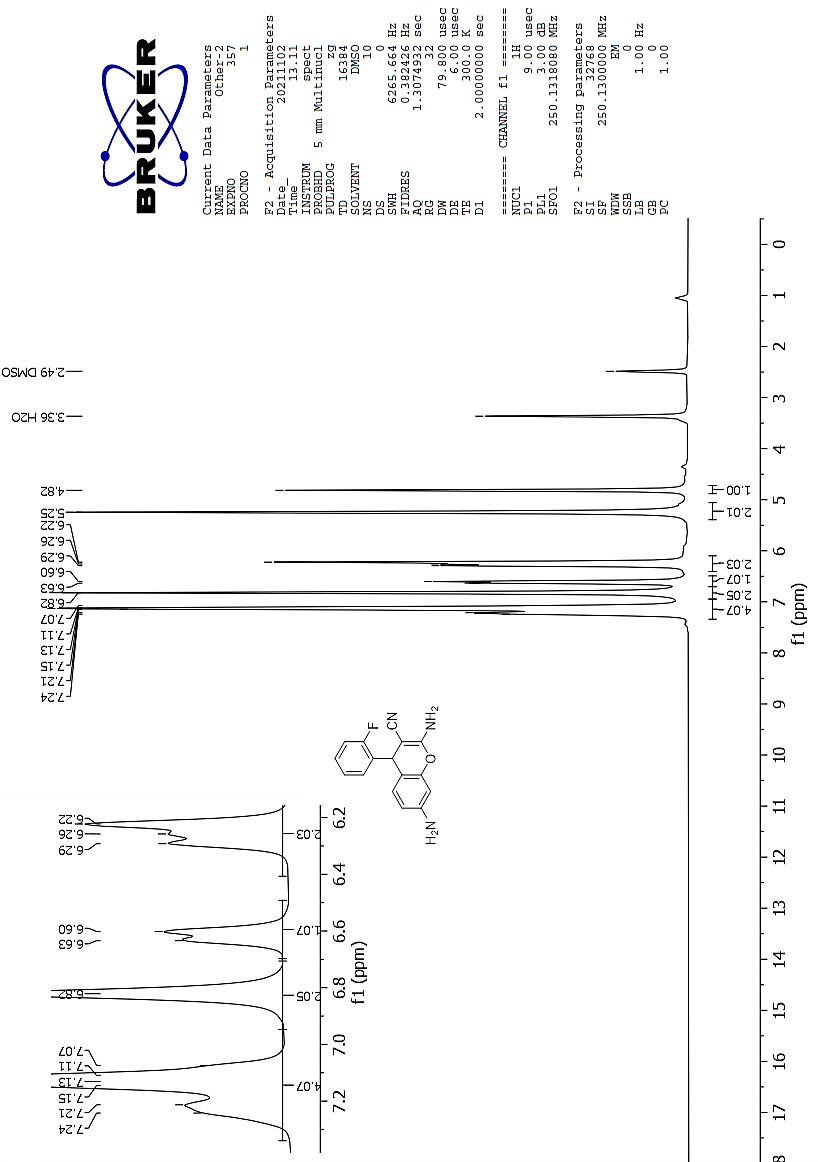 | | | **Figure S20. ^1^H NMR of spectra of 2,7-diamino-4-(2-fluorophenyl)-4H-chromene-3-carbonitrile** |
| --- | --- | --- | --- |
| 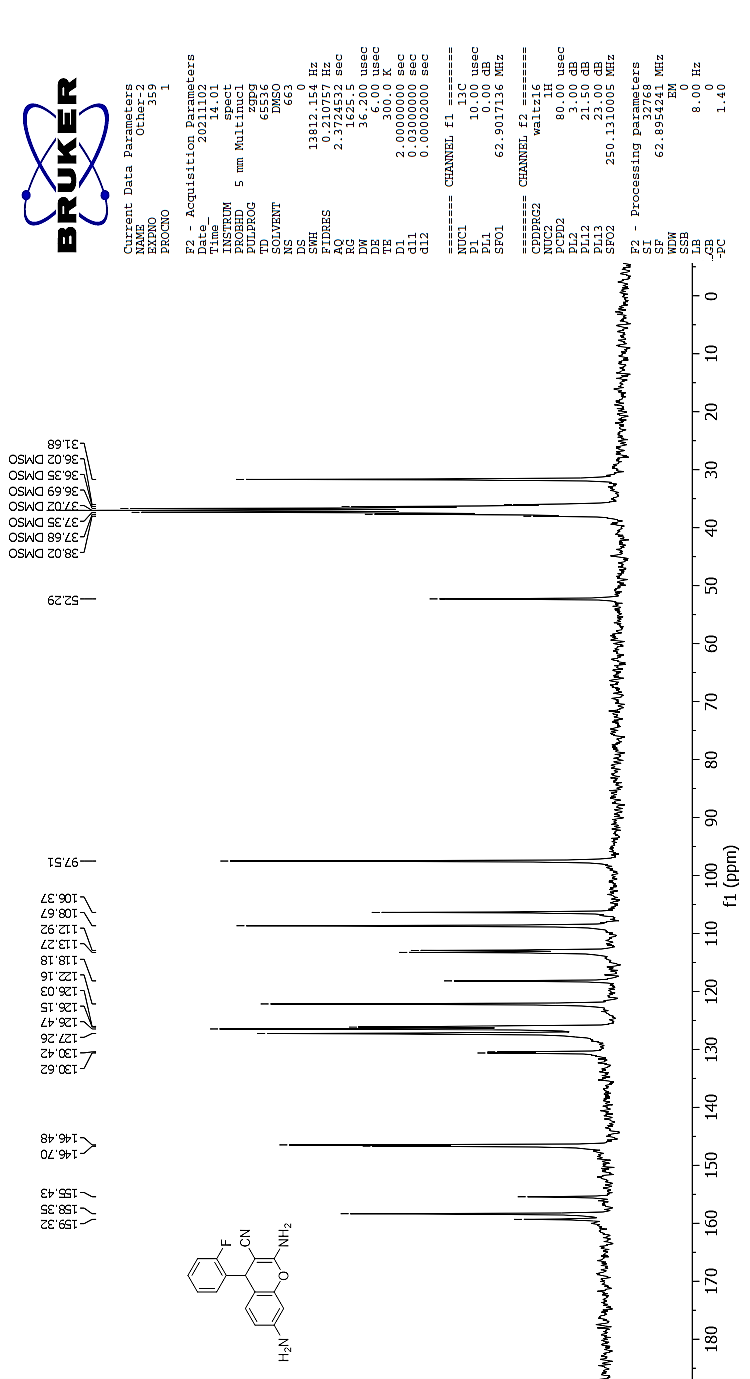 | **Figure S21. ^13^C NMR of spectra of 2,7-diamino-4-(2-fluorophenyl)-4H-chromene-3-carbonitrile** |  |  |

| 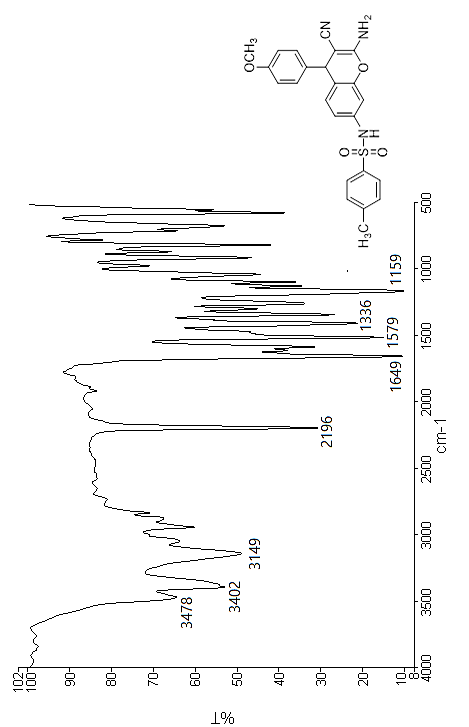 | **Figure S22. IR spectra of N-(2-amino-3-cyano-4-(4-methoxyphenyl)-4H-chromen-7-yl)-4-methylbenzenesulfonamide** |
| --- | --- |

| 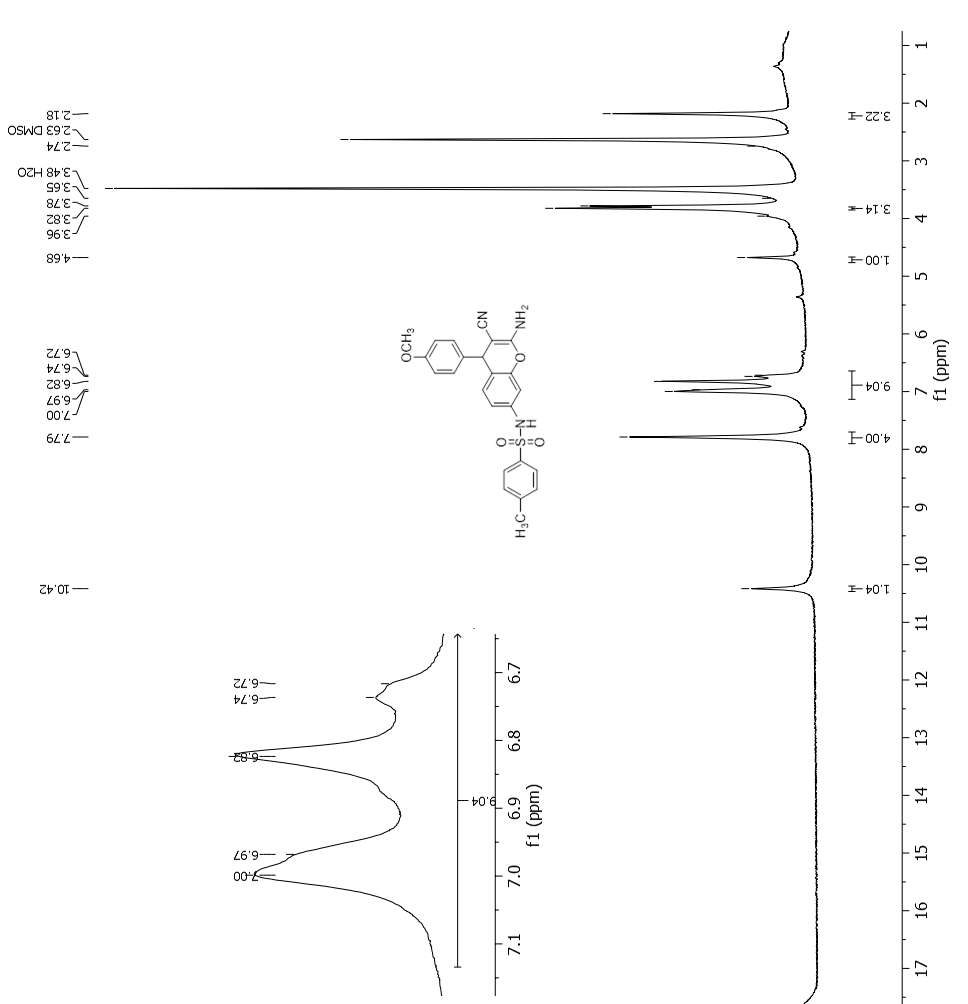 | **Figure S23. ^1^H NMR spectra of N-(2-amino-3-cyano-4-(4-methoxyphenyl)-4H-chromen-7-yl)-4-methylbenzenesulfonamide** |
| --- | --- |

| 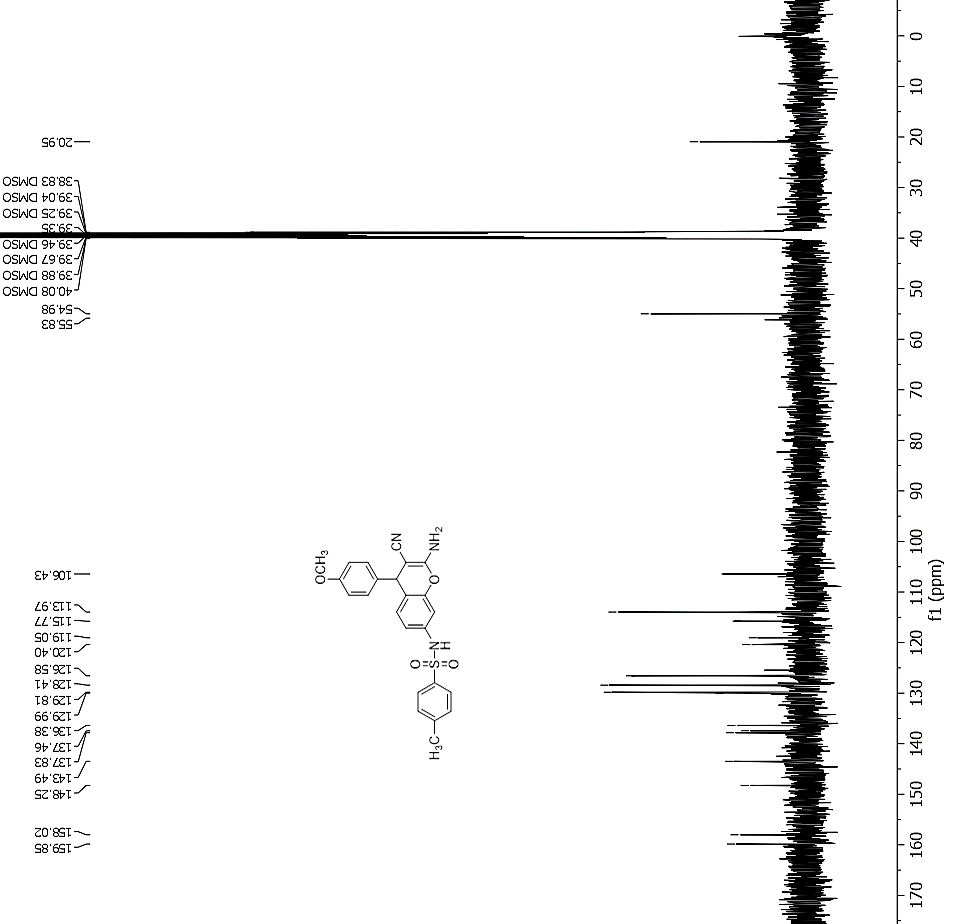 | **Figure S24. ^13^C NMR spectra of N-(2-amino-3-cyano-4-(4-methoxyphenyl)-4H-chromen-7-yl)-4-methylbenzenesulfonamide** |
| --- | --- |

| 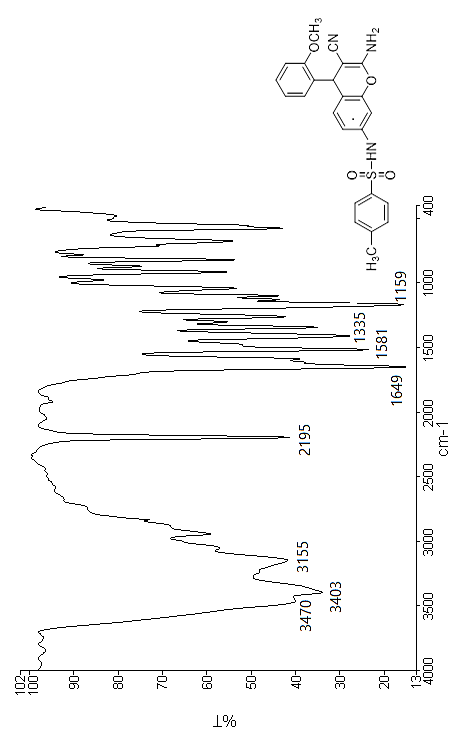 | **Figure25. IR spectra of N-(2-amino-3-cyano-4-(2-methoxyphenyl)-4H-chromen-7-yl)-4-methylbenzenesulfonamide** |
| --- | --- |

| 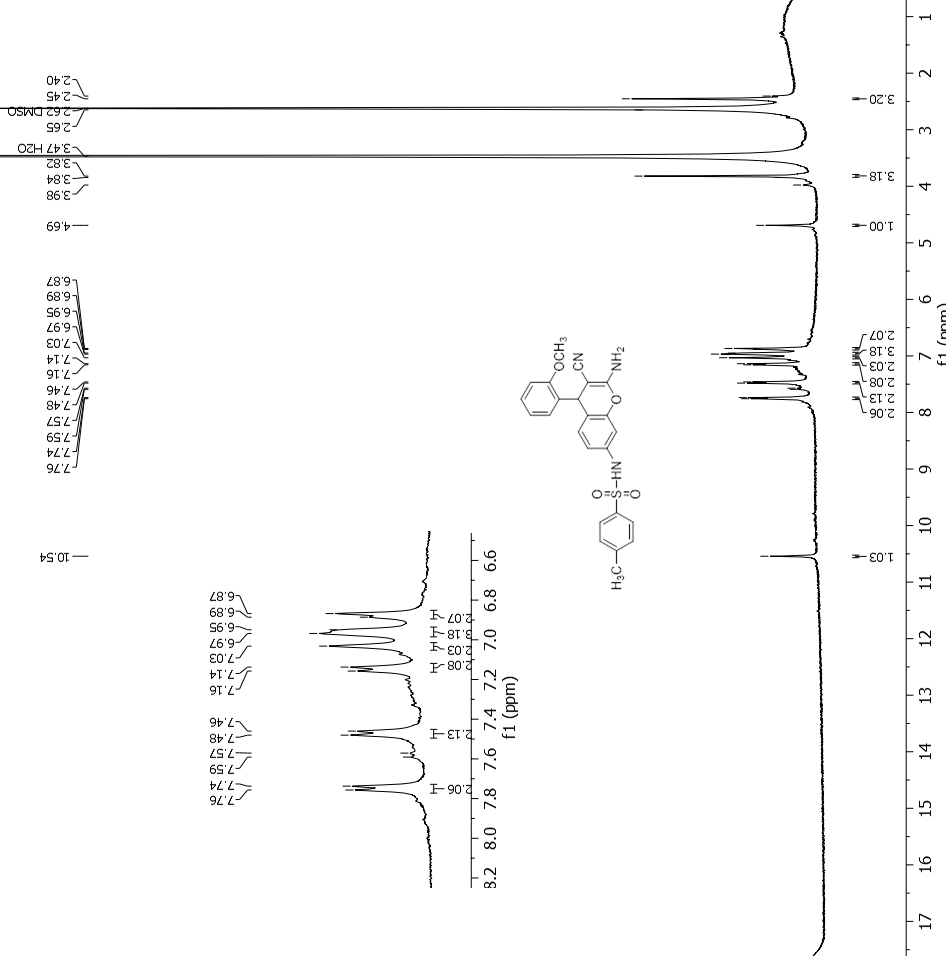 | **Figure S26.  ^1^H NMR of spectra of N-(2-amino-3-cyano-4-(2-methoxyphenyl)-4H-chromen-7-yl)-4-methylbenzenesulfonamide** |
| --- | --- |

| 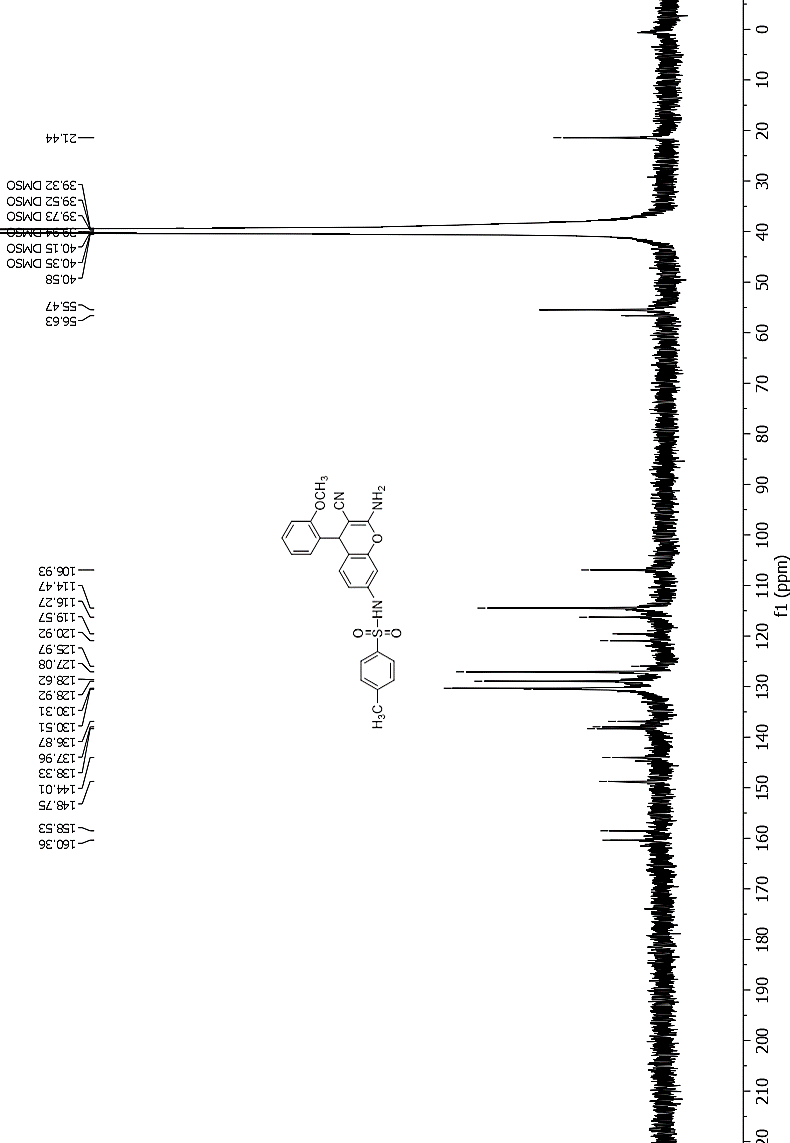 | **Figure S27. ^13^C NMR spectra of N-(2-amino-3-cyano-4-(2-methoxyphenyl)-4H-chromen-7-yl)-4-methylbenzenesulfonamide** |
| --- | --- |

| 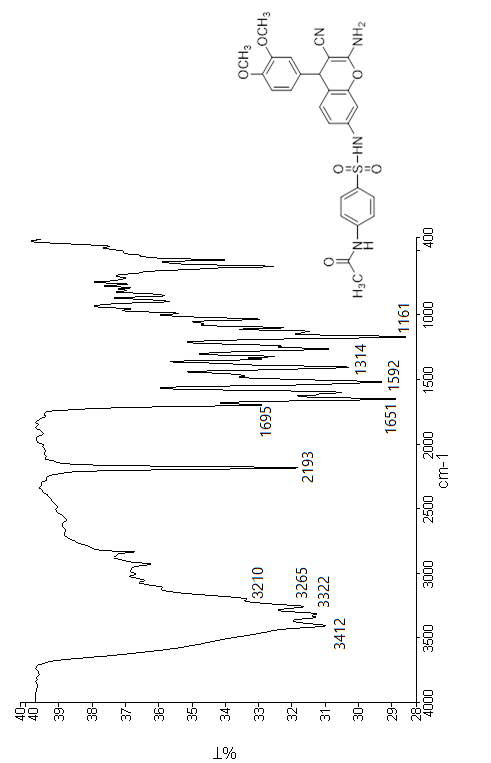 | **Figure 28. IR spectra of N-(2-amino-3-cyano-4-(3,4-dimethoxyphenyl)-4H-chromen-7-yl)-4-methylbenzenesulfonamide** |
| --- | --- |

| 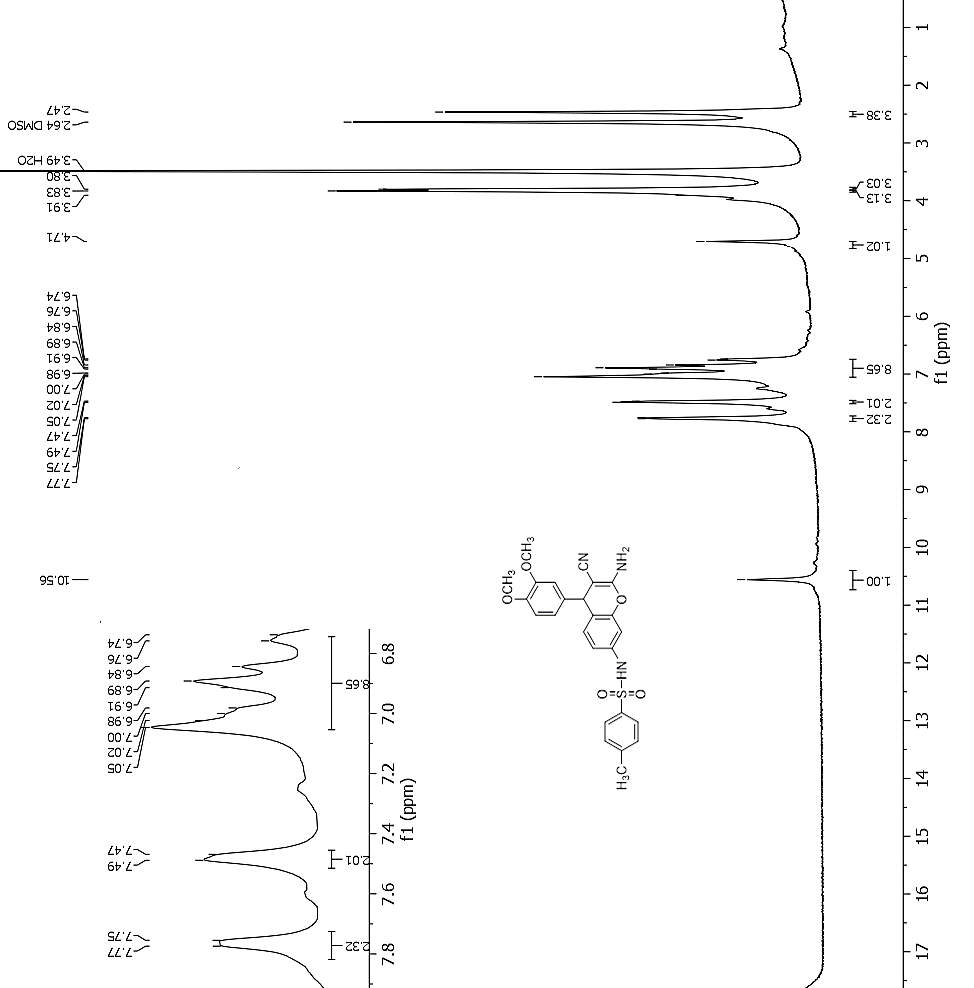 | **Figure S29. ^1^H NMR spectra of N-(2-amino-3-cyano-4-(3,4-dimethoxyphenyl)-4H-chromen-7-yl)-4-methylbenzenesulfonamide** |
| --- | --- |

| 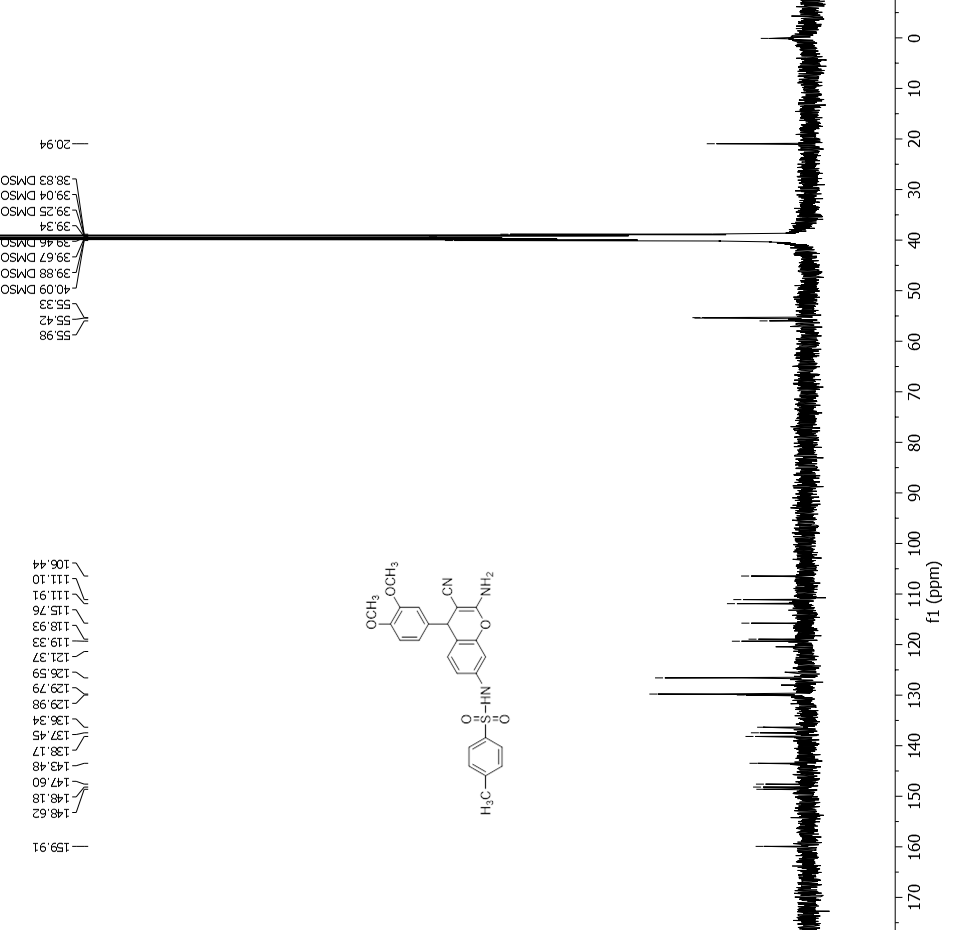 | **Figure S30. ^13^C NMR spectra of N-(2-amino-3-cyano-4-(3,4-dimethoxyphenyl)-4H-chromen-7-yl)-4-methylbenzenesulfonamide** |
| --- | --- |

| 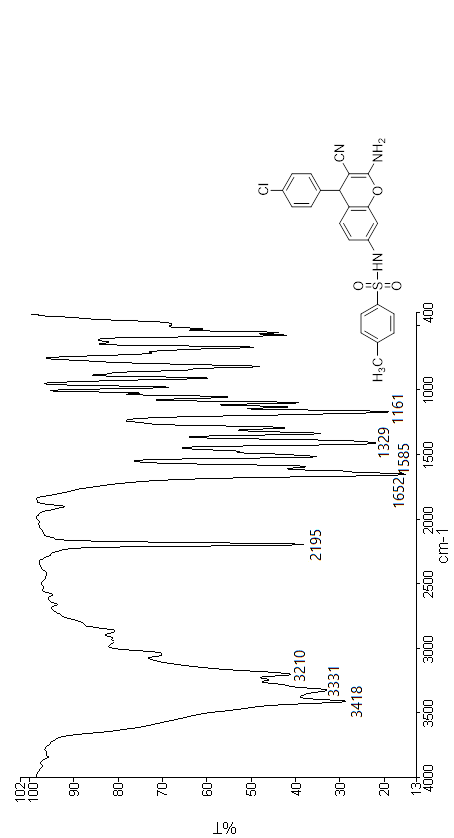 | **Figure 31. IR spectra of N-(2-amino-4-(4-chlorophenyl)-3-cyano-4H-chromen-7-yl)-4-methylbenzenesulfonamide** |
| --- | --- |

| 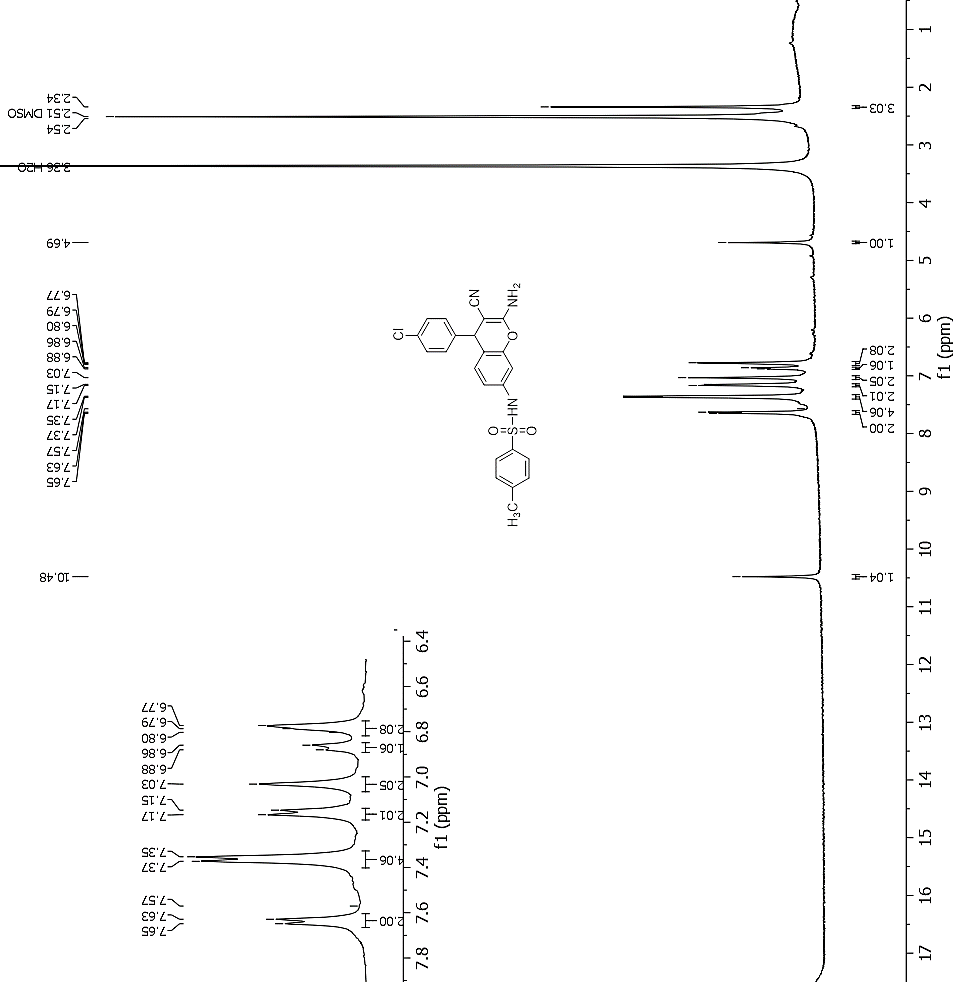 | **Figure S32. ^1^H NMR spectra of N-(2-amino-4-(4-chlorophenyl)-3-cyano-4H-chromen-7-yl)-4-methylbenzenesulfonamide** |
| --- | --- |

| 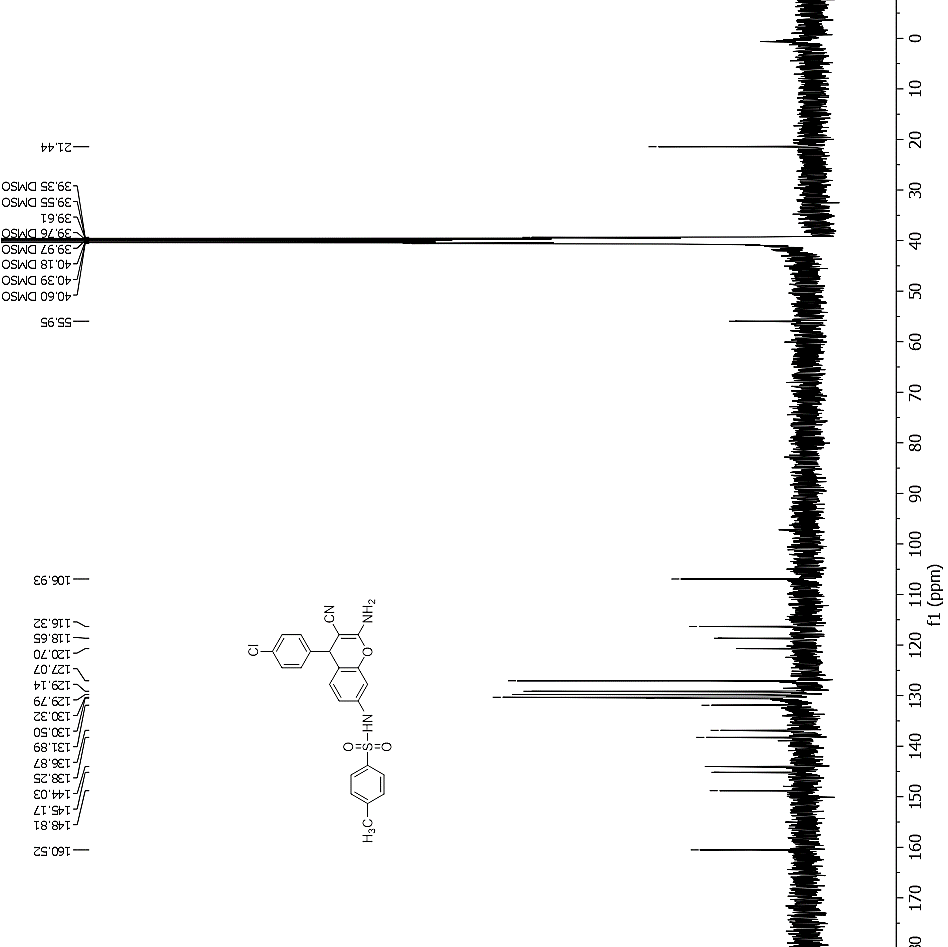 | | | **Figure S33. ^13^C NMR spectra of N-(2-amino-4-(4-chlorophenyl)-3-cyano-4H-chromen-7-yl)-4-methylbenzenesulfonamide** |
| --- | --- | --- | --- |
| 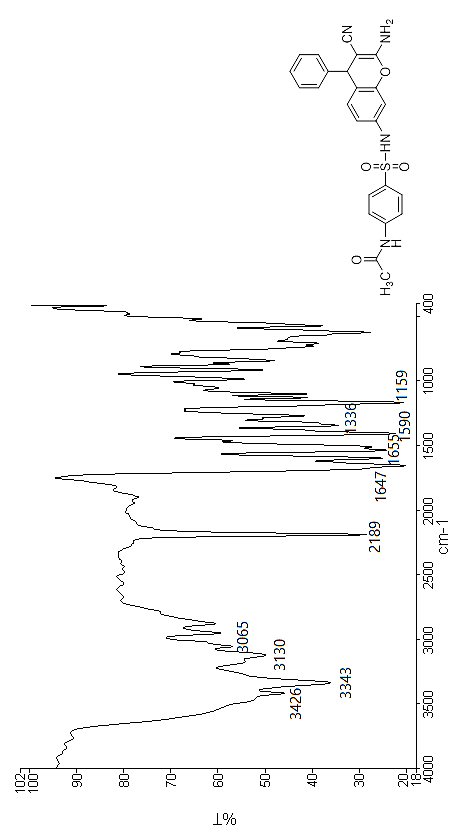 | **Figure 34. IR spectra of N-(4-(N-(2-amino-3-cyano-4-phenyl-4H-chromen-7-yl) sulfamoyl) phenyl) acetamide** |  |  |

| 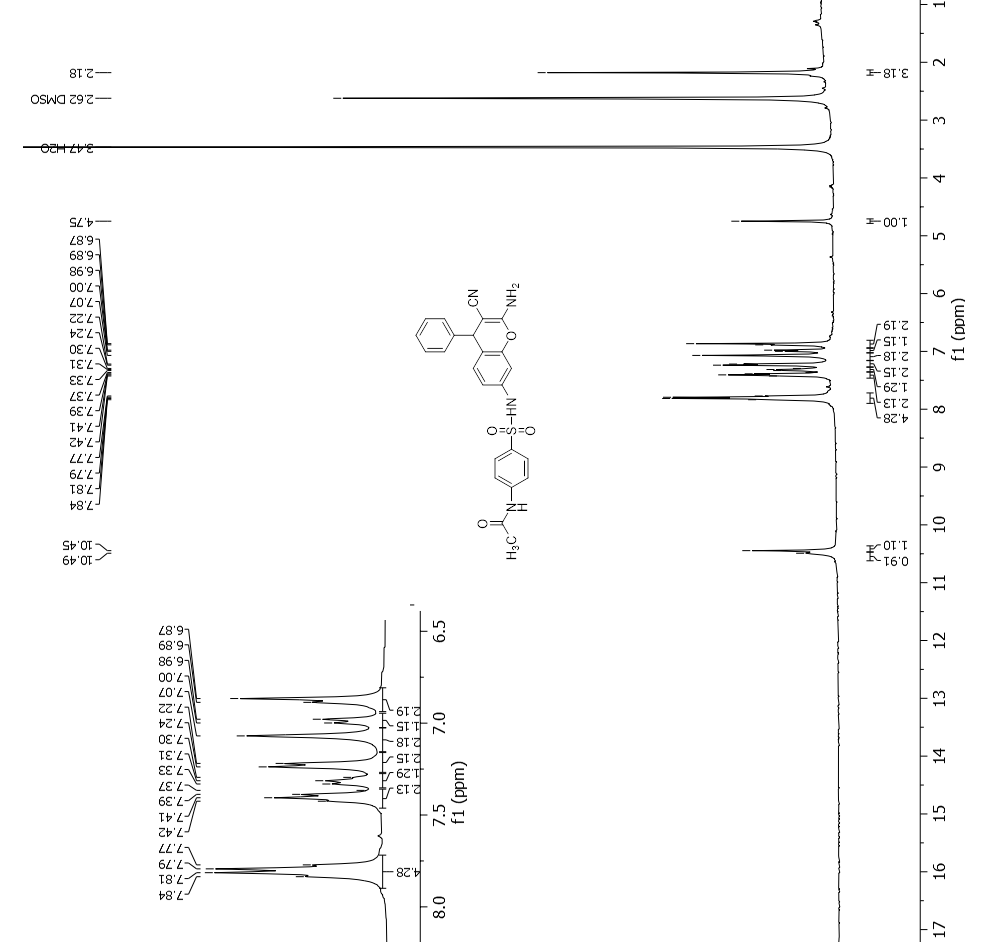 | **Figure S35. ^1^H NMR spectra of N-(4-(N-(2-amino-3-cyano-4-phenyl-4H-chromen-7-yl)sulfamoyl)phenyl)acetamide** |
| --- | --- |

| 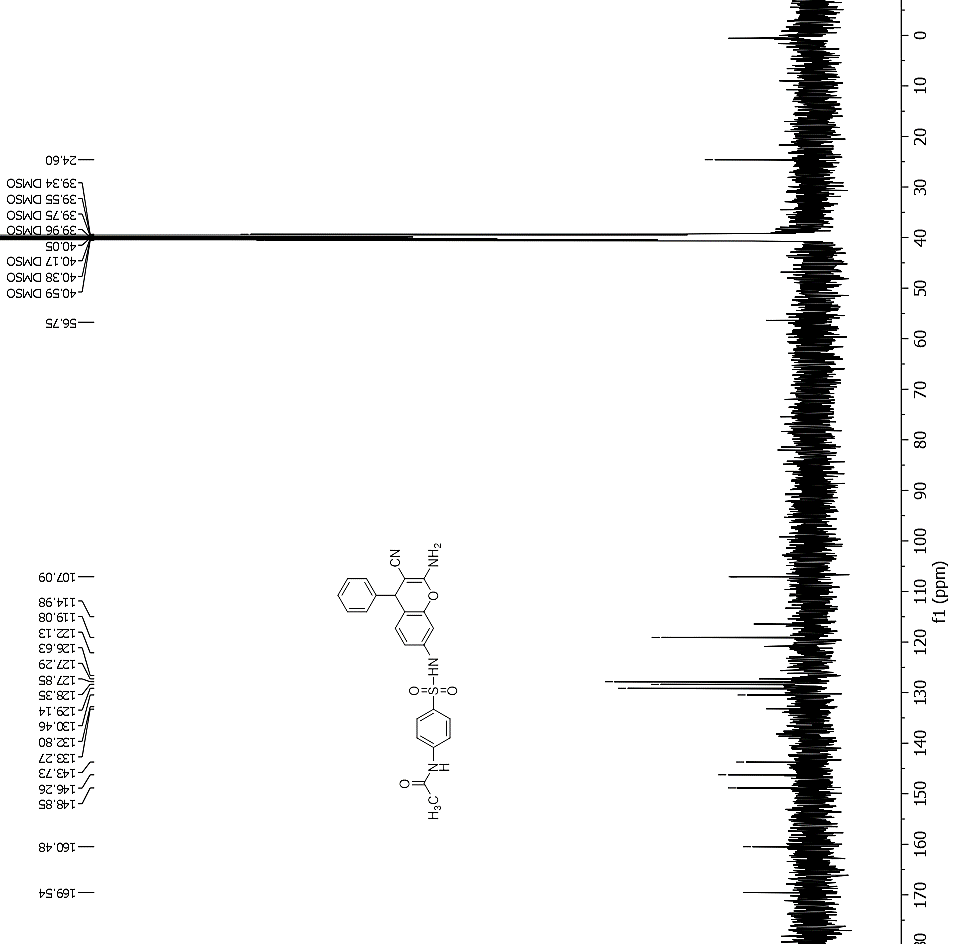 | **Figure S36. ^13^C NMR spectra of N-(4-(N-(2-amino-3-cyano-4-phenyl-4H-chromen-7-yl)sulfamoyl)phenyl)acetamide** |
| --- | --- |

| 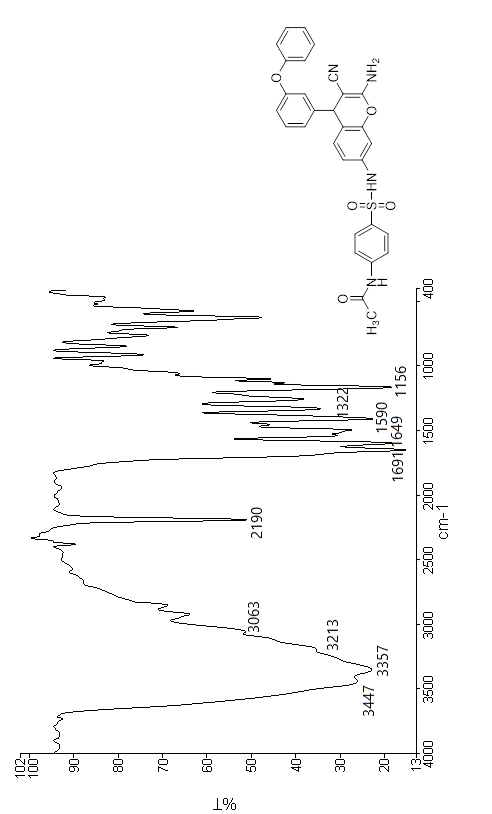 | **Figure 37. IR spectra of N-(4-(N-(2-amino-3-cyano-4-(3-phenoxyphenyl)-4H-chromen-7-yl) sulfamoyl) phenyl) acetamide** |
| --- | --- |

| 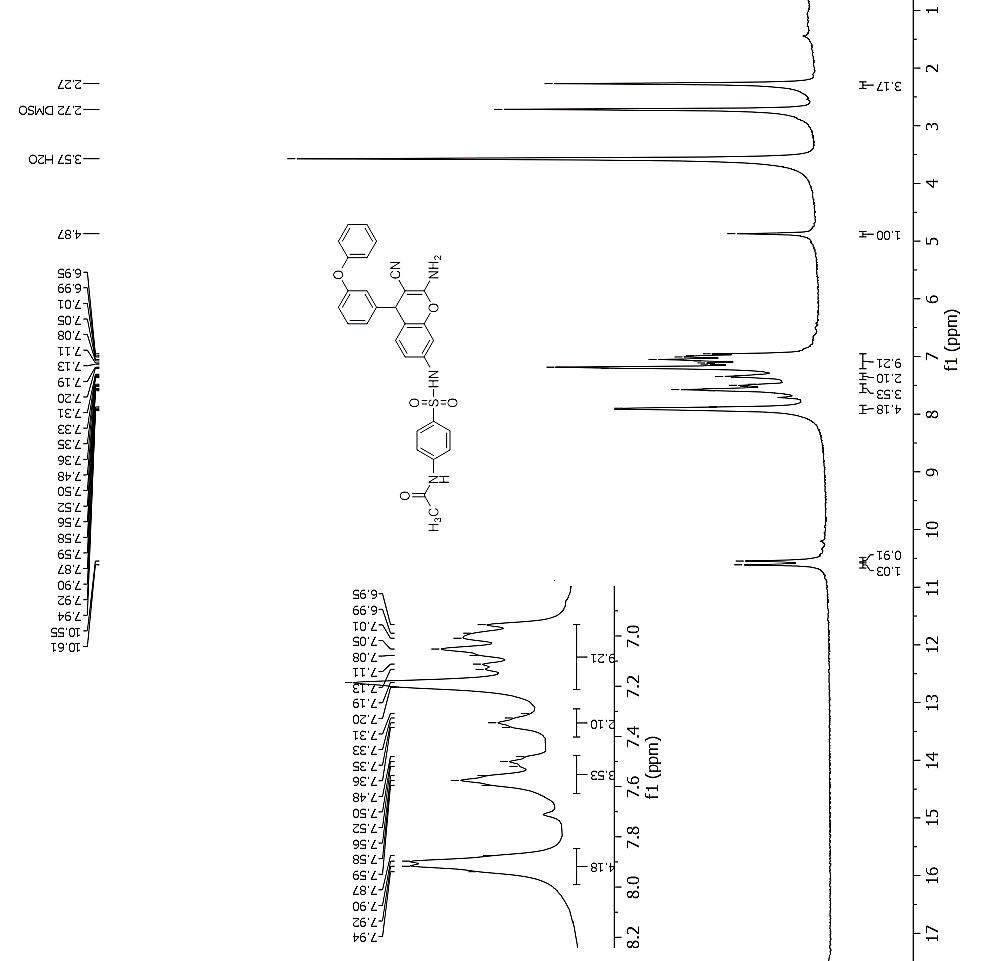 | **Figure S38. ^1^H NMR spectra of N-(4-(N-(2-amino-3-cyano-4-(3-phenoxyphenyl)-4H-chromen-7-yl) sulfamoyl) phenyl) acetamide** |
| --- | --- |

| 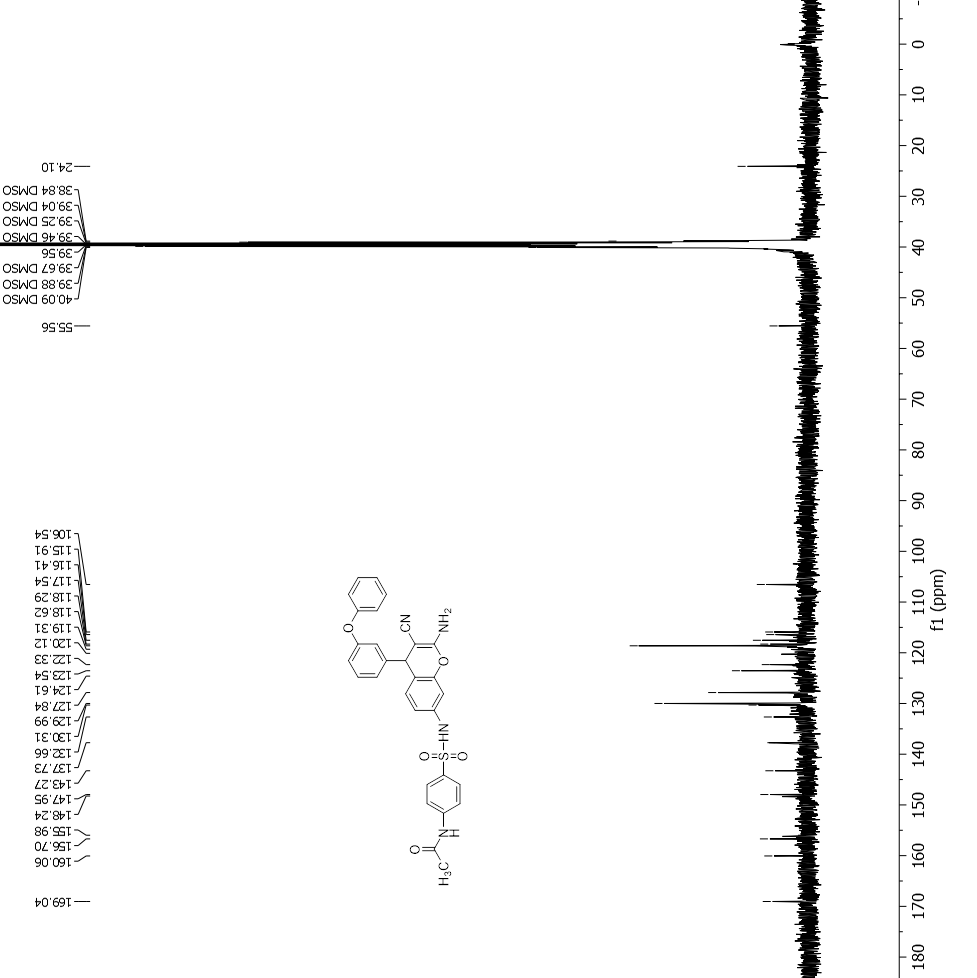 | **Figure S39. ^13^C NMR spectra of N-(4-(N-(2-amino-3-cyano-4-(3-phenoxyphenyl)-4H-chromen-7-yl) sulfamoyl) phenyl) acetamide** |
| --- | --- |

| 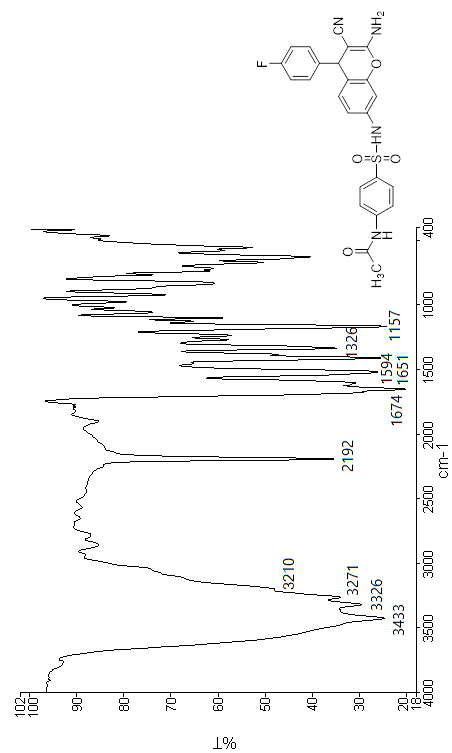 | **Figure 40. IR spectra of N-(4-(N-(2-amino-3-cyano-4-(4-fluorophenyl)-4H-chromen-7-yl) sulfamoyl) phenyl) acetamide** |
| --- | --- |

| 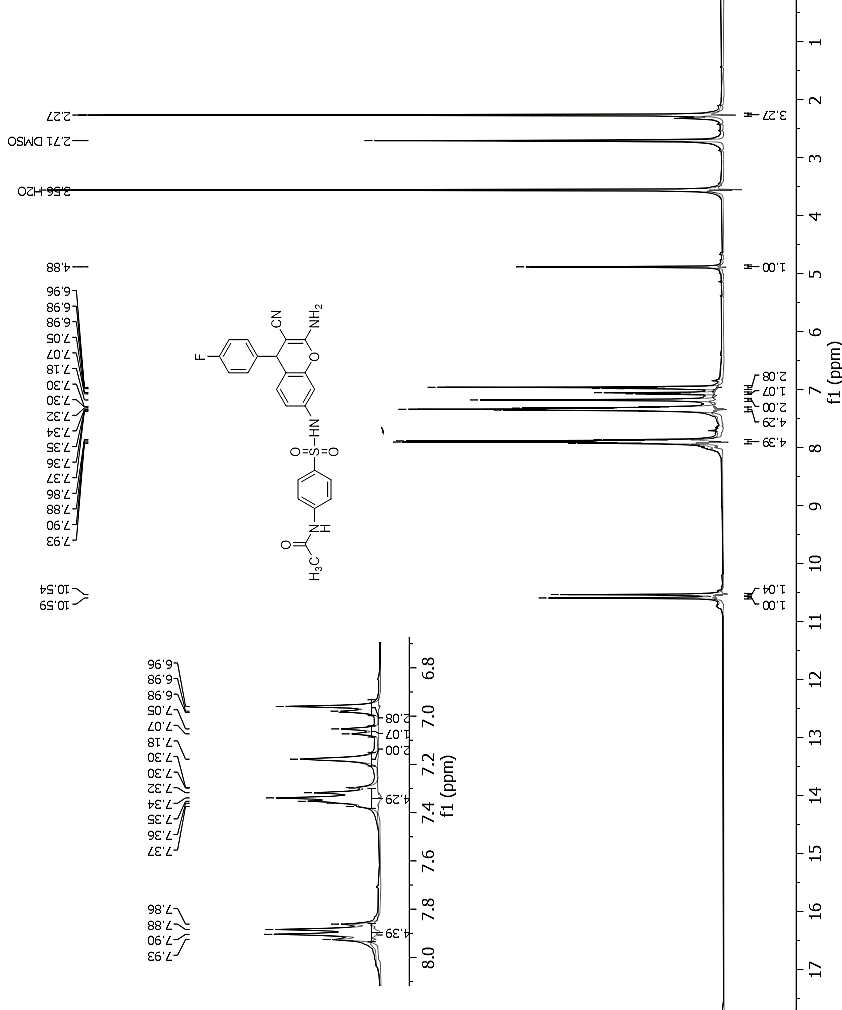 | **Figure S41. ^1^H NMRspectra of N-(4-(N-(2-amino-3-cyano-4-(4-fluorophenyl)-4H-chromen-7-yl) sulfamoyl) phenyl) acetamide** |
| --- | --- |

| 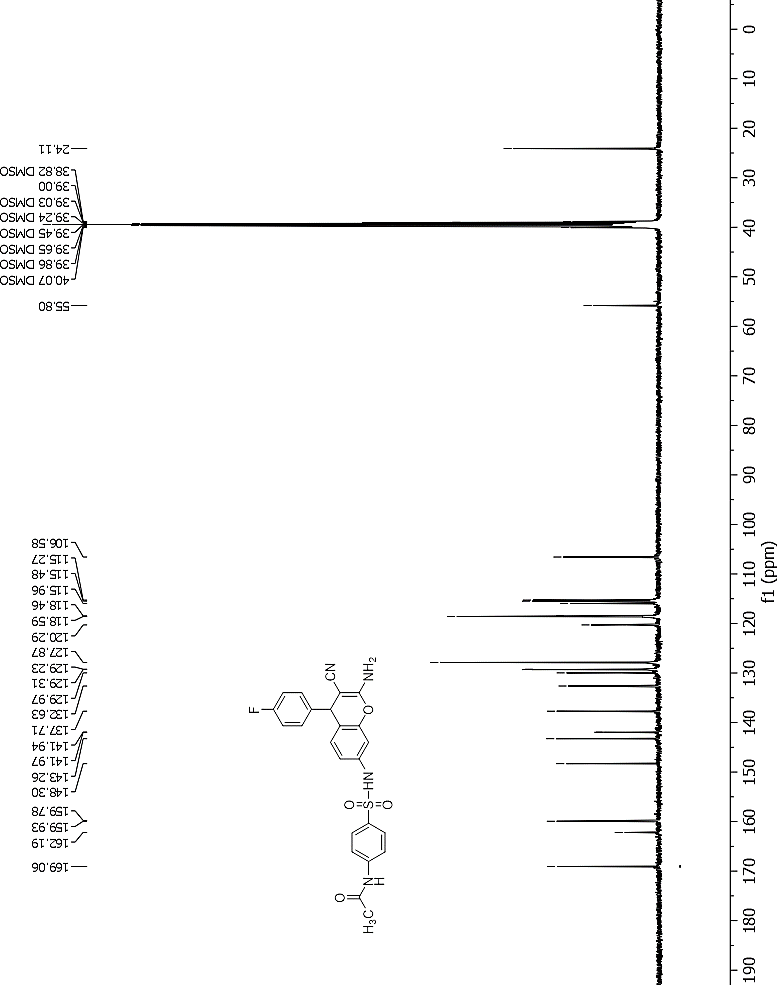 | **Figure S42. ^13^C NMR spectra of N-(4-(N-(2-amino-3-cyano-4-(4-fluorophenyl)-4H-chromen-7-yl) sulfamoyl) phenyl) acetamide** |
| --- | --- |

| 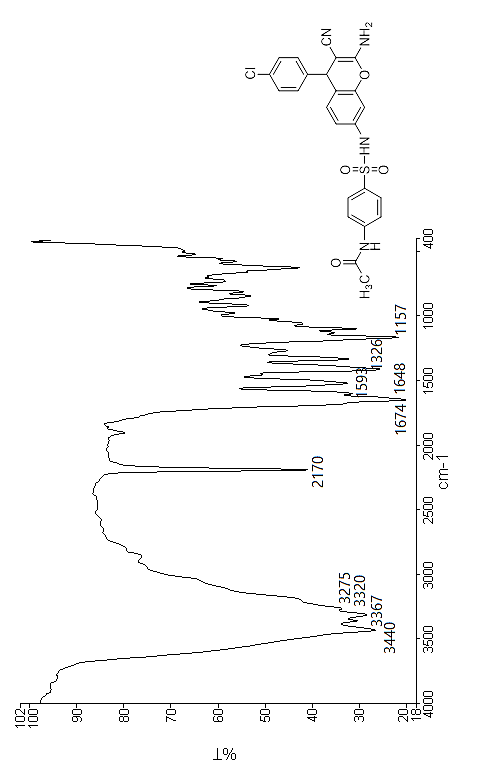 | **Figure 43. IR spectra of N-(4-(N-(2-amino-4-(4-chlorophenyl)-3-cyano-4H-chromen-7-yl) sulfamoyl) phenyl) acetamide** |
| --- | --- |

| 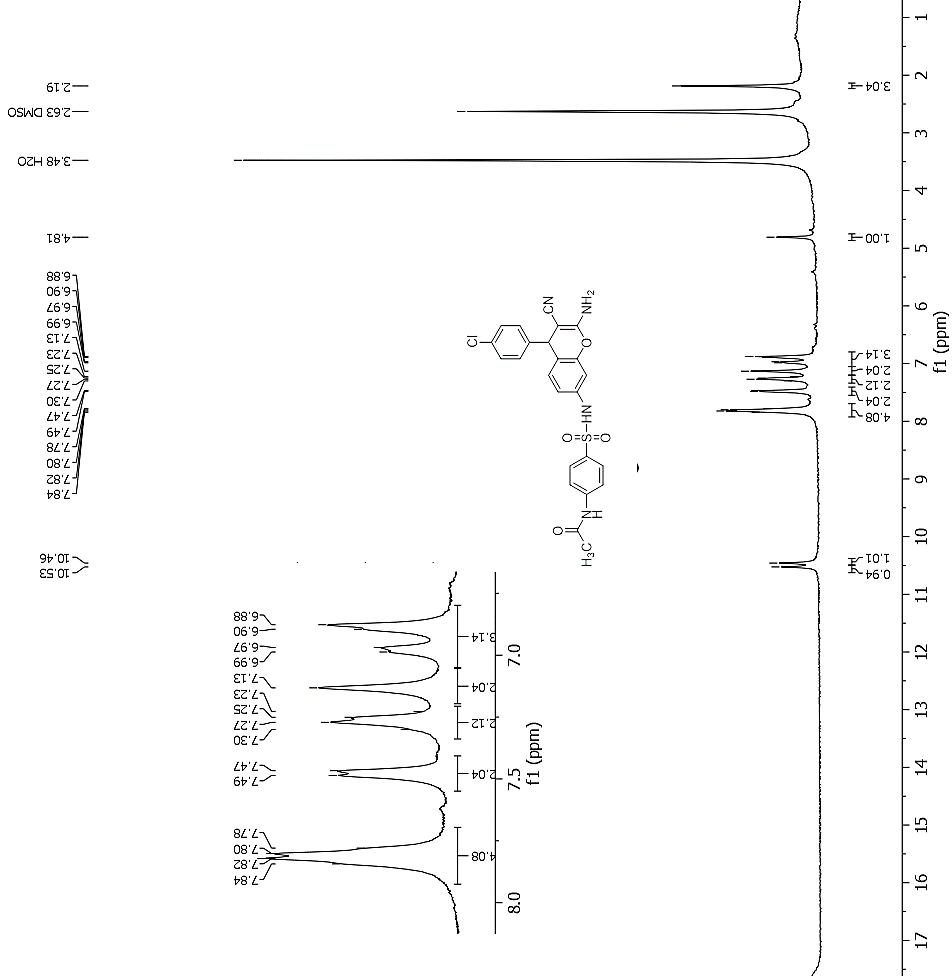 | **Figure S44. ^1^H NMR spectra of N-(4-(N-(2-amino-4-(4-chlorophenyl)-3-cyano-4H-chromen-7-yl) sulfamoyl) phenyl) acetamide** |
| --- | --- |

| 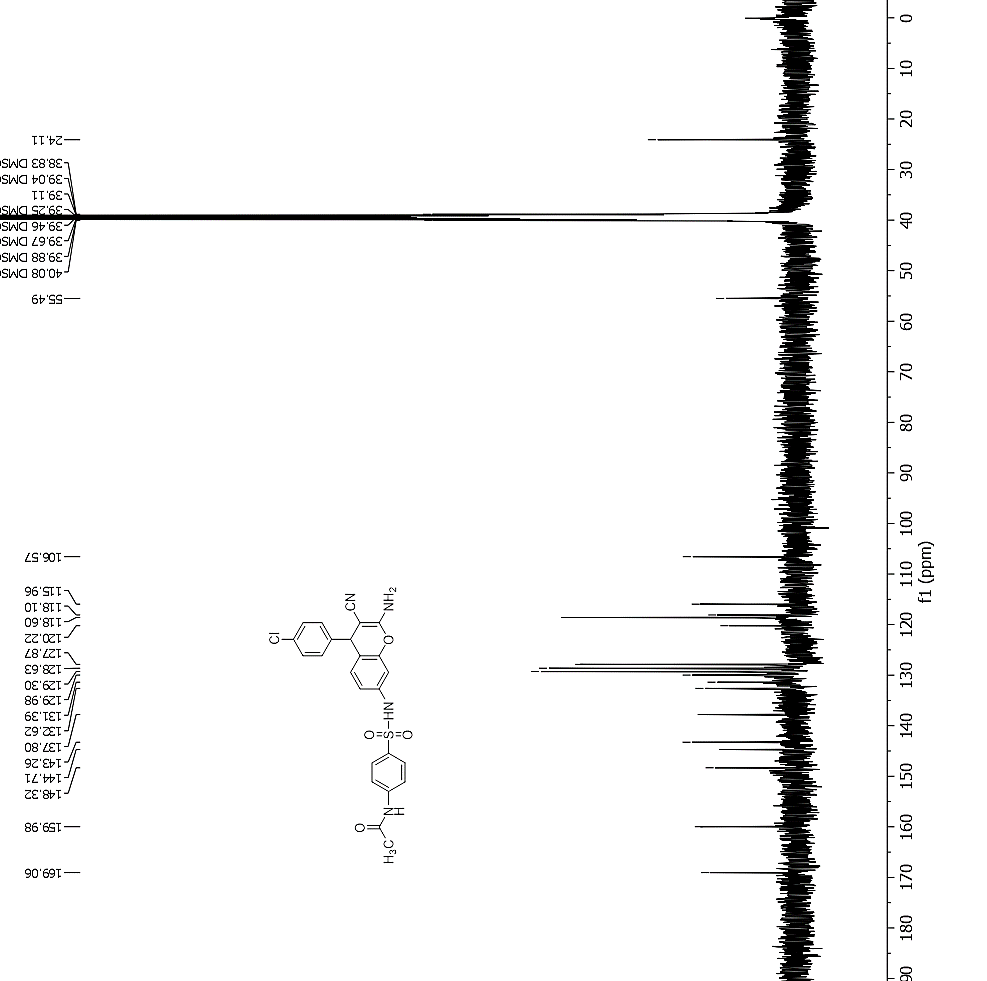 | **Figure S45. ^13^C NMR spectra of N-(4-(N-(2-amino-4-(4-chlorophenyl)-3-cyano-4H-chromen-7-yl) sulfamoyl) phenyl) acetamide** |
| --- | --- |

| 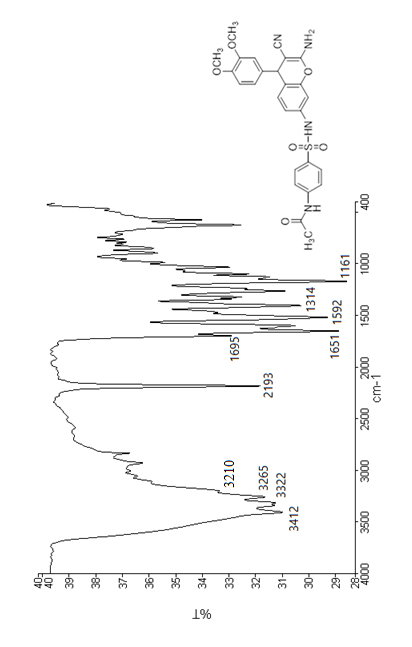 | **Figure 46. IR spectra of N-(4-(N-(2-amino-3-cyano-4-(3,4-dimethoxyphenyl)-4H-chromen-7-yl) sulfamoyl) phenyl) acetamide** |
| --- | --- |

| 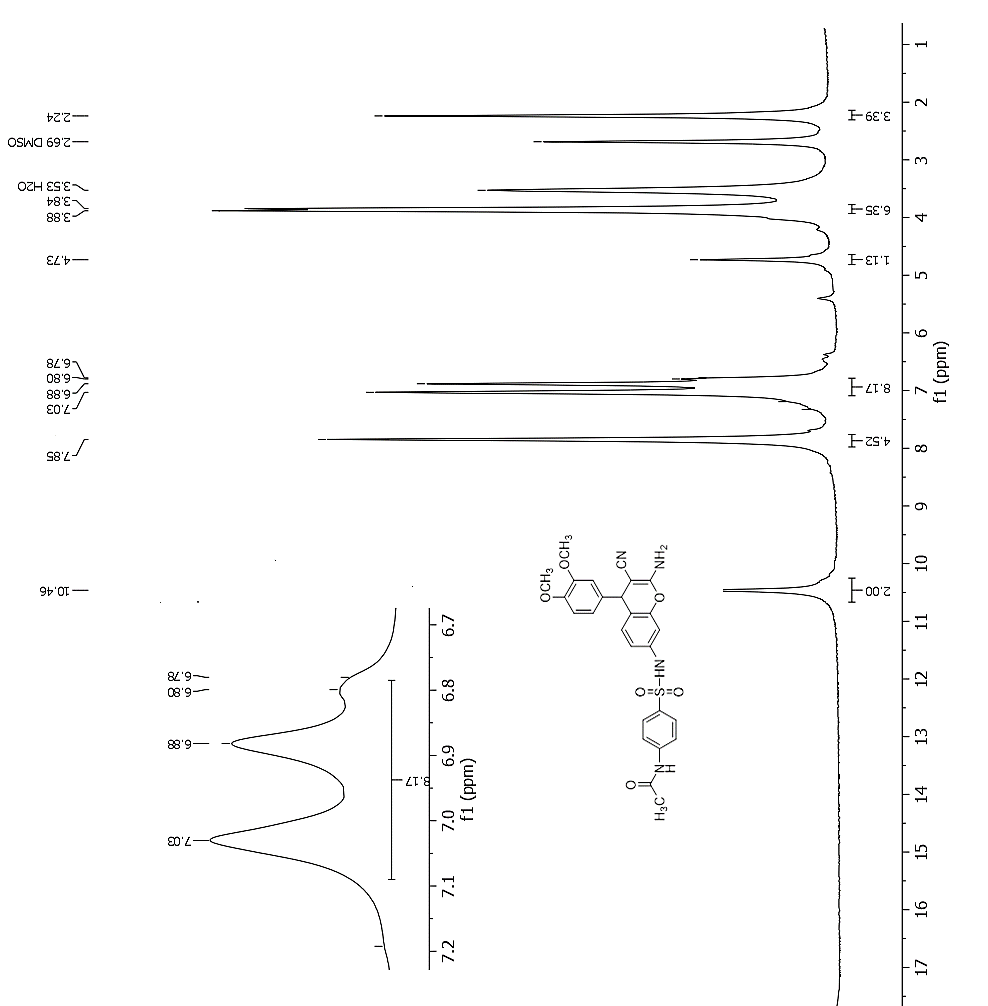 | **Figure S47. ^1^H NMR spectra of N-(4-(N-(2-amino-3-cyano-4-(3,4-dimethoxyphenyl)-4H-chromen-7-^1^H NMRyl)sulfamoyl)phenyl)acetamide** |
| --- | --- |

| 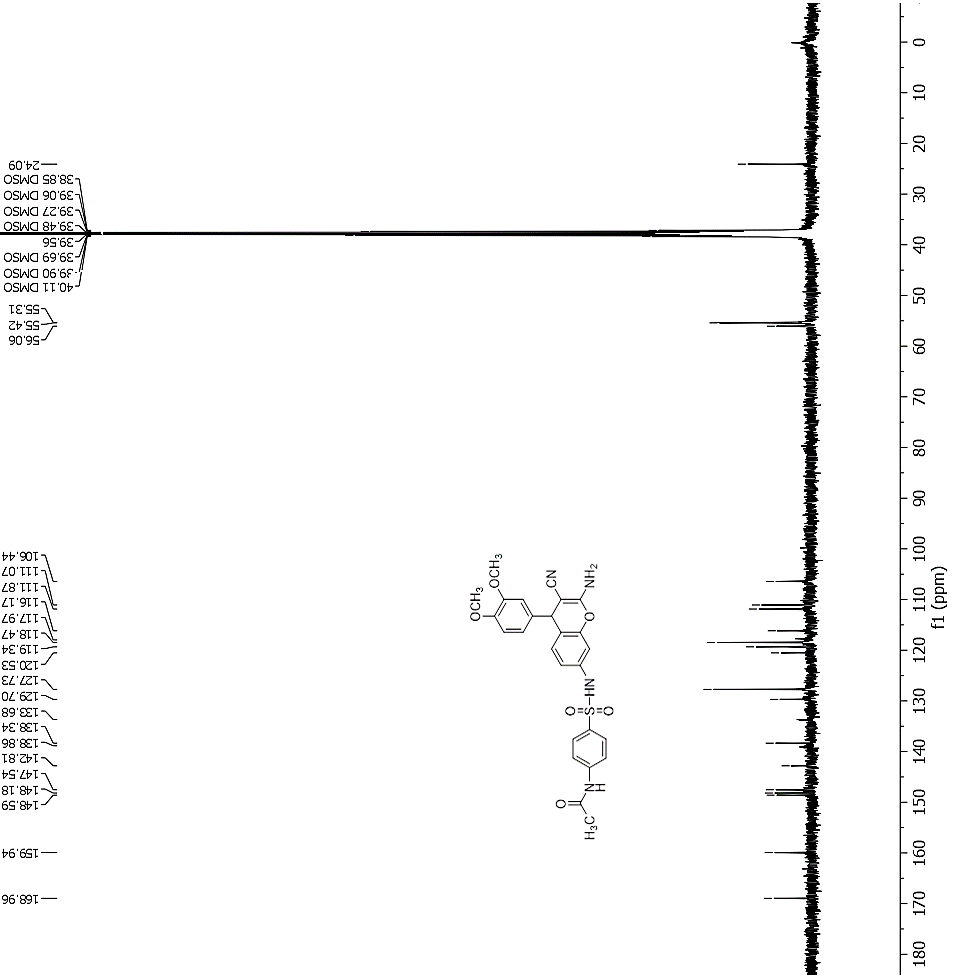 | **Figure S48. ^13^C NMR spectra of N-(4-(N-(2-amino-3-cyano-4-(3,4-dimethoxyphenyl)-4H-chromen-7-yl) sulfamoyl)phenyl)acetamide** |
| --- | --- |

| 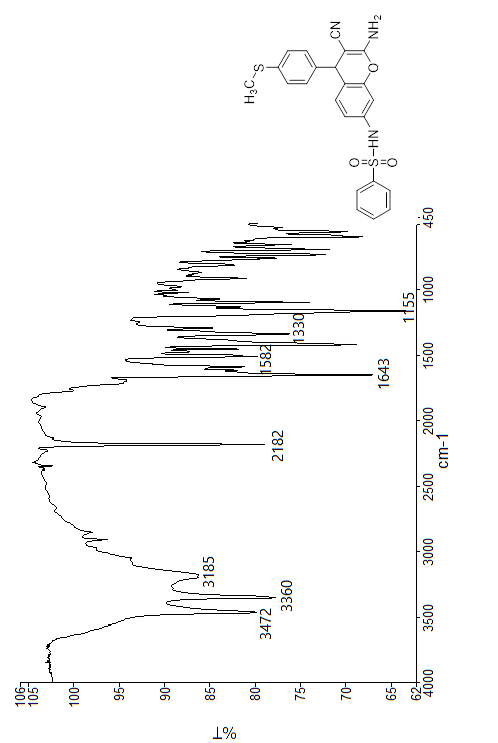 | **Figure 49. IR N-(2-amino-3-cyano-4-(4-(methylthio) phenyl)-4H-chromen-7-yl) benzenesulfonamide** |
| --- | --- |

| 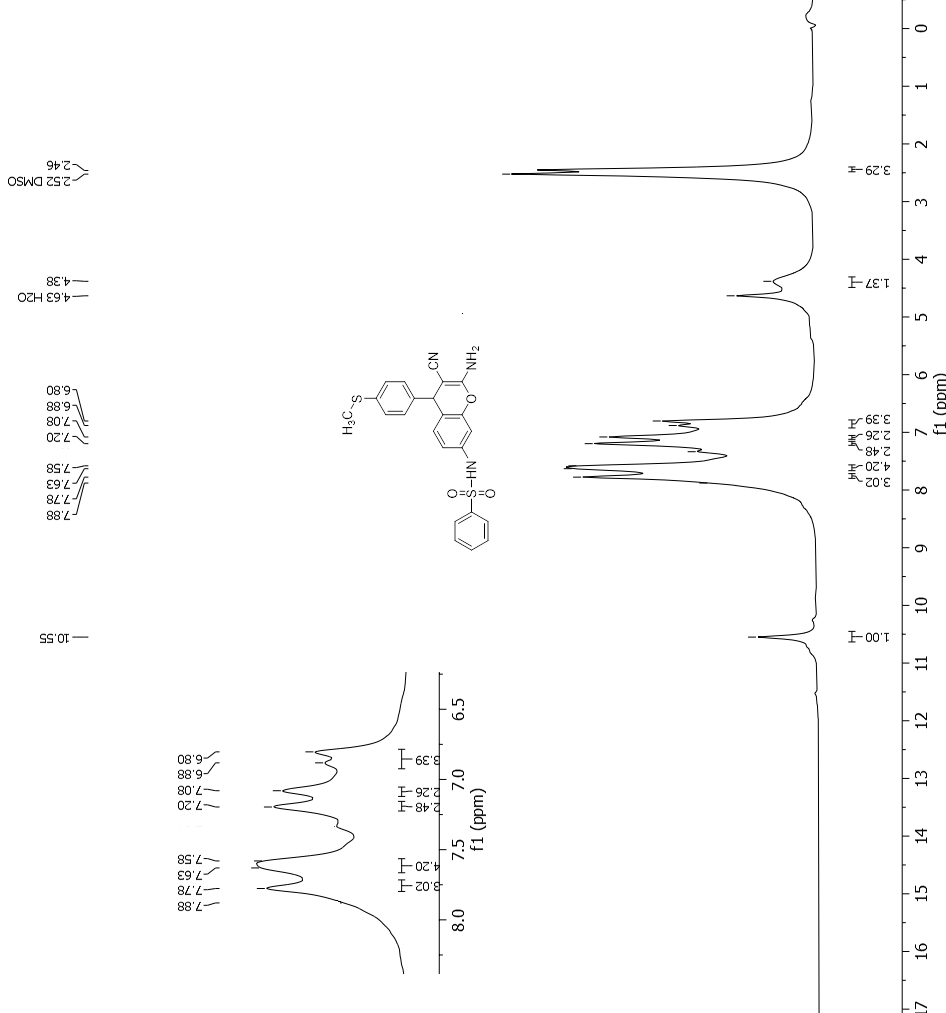 | **Figure S50. ^1^H NMR spectra of N-(2-amino-3-cyano-4-(4-(methylthio) phenyl)-4H-chromen-7-yl) benzenesulfonamide** |
| --- | --- |

| 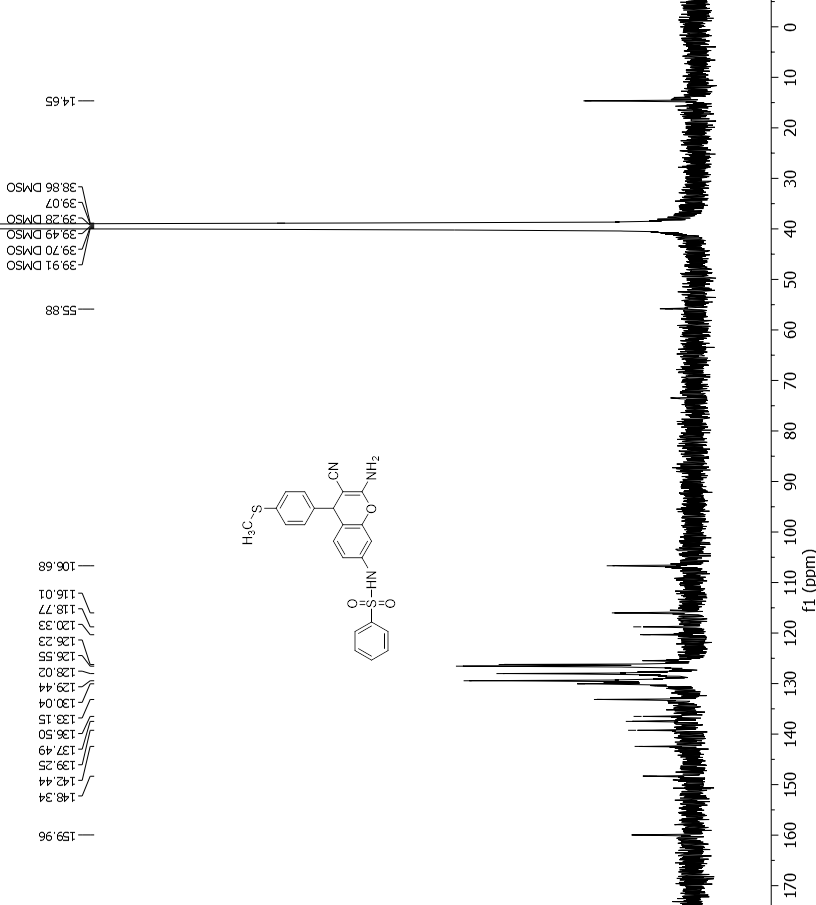 | **Figure S51. ^13^C NMR spectra of N-(2-amino-3-cyano-4-(4-(methylthio) phenyl)-4H-chromen-7-yl) benzenesulfonamide** |
| --- | --- |

| 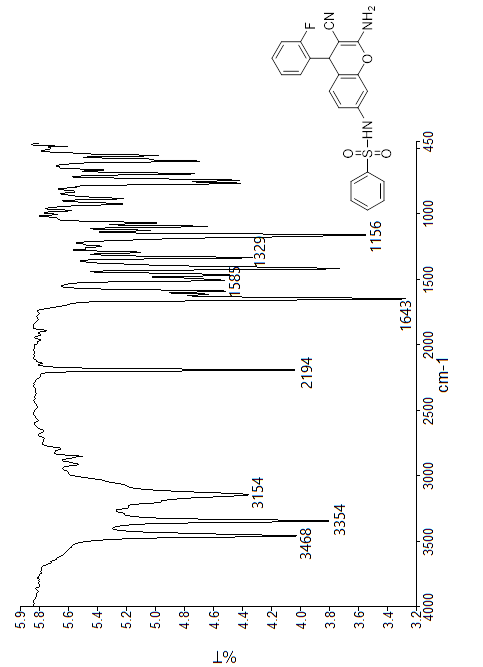 | **Figure 52. IR spectra of N-(2-amino-3-cyano-4-(2-fluorophenyl)-4H-chromen-7-yl) benzenesulfonamide** |
| --- | --- |

| 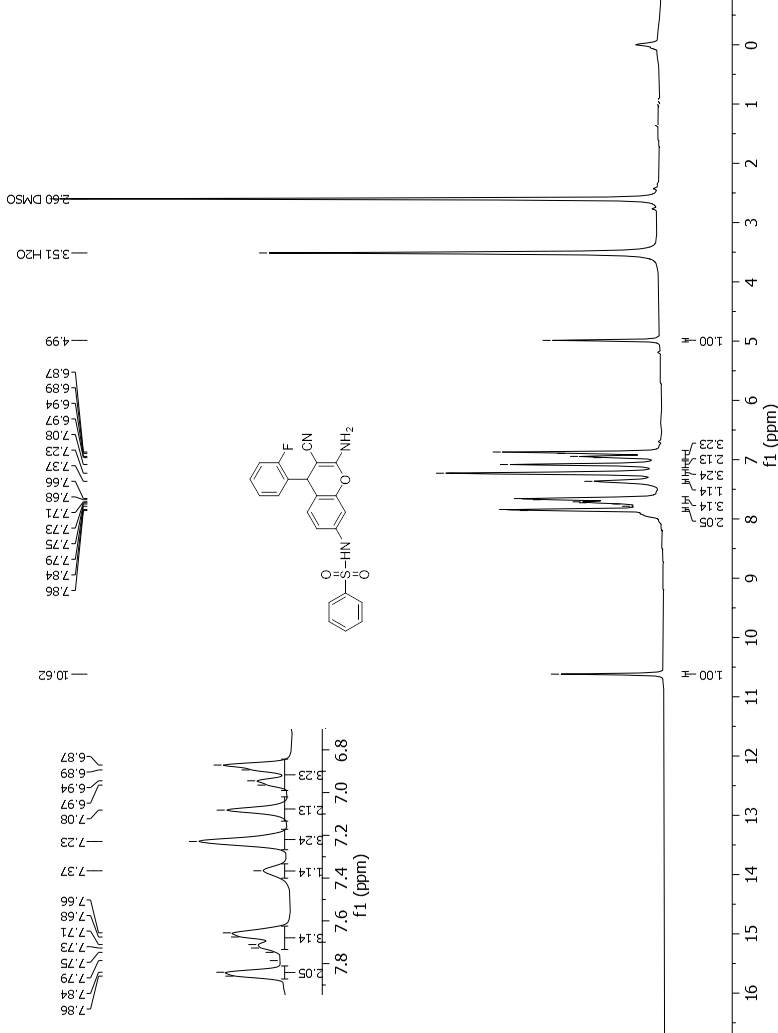 | **Figure S53. ^1^H NMR spectra of N-(2-amino-3-cyano-4-(2-fluorophenyl)-4H-chromen-7-yl) benzenesulfonamide** |
| --- | --- |
| 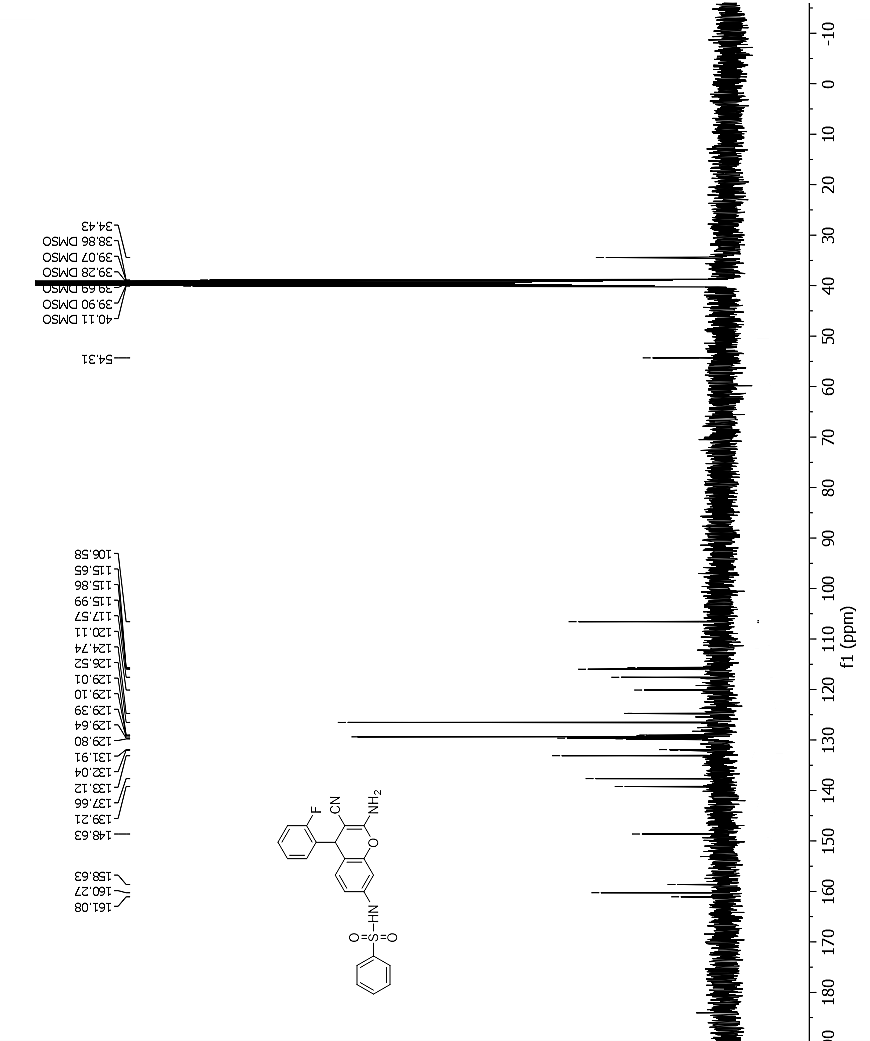 | **Figure S54. ^13^C NMR spectra of N-(2-amino-3-cyano-4-(2-fluorophenyl)-4H-chromen-7-yl) benzenesulfonamide** |
